# Gene expression study and pathway analysis of histological subtypes of intestinal metaplasia that progress to gastric cancer

**DOI:** 10.1371/journal.pone.0176043

**Published:** 2017-04-25

**Authors:** Osmel Companioni, José Miguel Sanz-Anquela, María Luisa Pardo, Eulàlia Puigdecanet, Lara Nonell, Nadia García, Verónica Parra Blanco, Consuelo López, Victoria Andreu, Miriam Cuatrecasas, Maddi Garmendia, Javier P. Gisbert, Carlos A. Gonzalez, Núria Sala

**Affiliations:** 1 Unit of Nutrition and Cancer, Cancer Epidemiology Research Program, Institut Català d’Oncologia, Barcelona, Spain; 2 Cancer Registry and Pathology Department, Hospital Universitario Príncipe de Asturias and Department of Medicine and Medical Specialties, Faculty of Medicine, University of Alcalá, Alcalá de Henares, Madrid, Spain; 3 Pathology Department, Complejo Hospitalario de Soria, Spain; 4 Microarray Analysis Service, IMIM (Hospital del Mar Medical Research Institute), Barcelona, Spain; 5 Department of Histopathology, Hospital Universitario Gregorio Marañón, Madrid, Spain; 6 Department of Pathology, Hospital Universitario de la Princesa, Madrid, Spain; 7 Department of Gastroenterology, Hospital de Viladecans, Spain; 8 Department of Pathology, Hospital Clínic de Barcelona, Universitat de Barcelona, Biobanc Clinic IDIBAPS, Barcelona, Spain; 9 Department of Pathology, and Department of Gastroenterology, Hospital Donostia/Instituto Biodonostia, Universidad del País Vasco (UPV/EHU), CIBEREHD, San Sebastián, Spain; 10 Gastroenterology Unit, Hospital Universitario de La Princesa and Instituto de Investigación Sanitaria Princesa (IIS-IP), Centro de Investigación Biomédica en Red de Enfermedades Hepáticas y Digestivas (CIBEREHD), Madrid, Spain; Queen's University Belfast, UNITED KINGDOM

## Abstract

**Background:**

Intestinal metaplasia (IM) is a precursor lesion that precedes gastric cancer (GC). There are two IM histological subtypes, complete (CIM) and incomplete (IIM), the latter having higher progression rates to GC. This study was aimed at analysing gene expression and molecular processes involved in the progression from normal mucosa to IM, and also from IM subtypes to GC.

**Methodology:**

We used expression data to compare the transcriptome of healthy gastric mucosa to that of IM not progressing to GC, and the transcriptome of IM subtypes that had progressed to GC to those that did not progress. Some deregulated genes were validated and pathway analyses were performed.

**Results:**

Comparison of IM subtypes that had progressed to GC with those that did not progress showed smaller differences in the expression profiles than the comparison of IM that did not progress with healthy mucosa. New transcripts identified in IM not progressing to GC included TRIM, TMEM, homeobox and transporter genes and SNORD116. Comparison to normal mucosa identified non tumoral Warburg effect and melatonin degradation as previously unreported processes involved in IM. Overexpressed antigen processing is common to both IM-subtypes progressing to GC, but IIM showed more over-expressed oncogenic genes and molecular processes than CIM.

**Conclusions:**

There are greater differences in gene expression and molecular processes involved in the progression from normal healthy mucosa to IM than from IM to gastric cancer. While antigen processing is common in both IM-subtypes progressing to GC, more oncogenic processes are observed in the progression of IIM.

## Introduction

Gastric carcinogenesis proceeds through a series of precursor lesions in the gastric mucosa named the Correa´s cascade, constituted by multi-atrophic gastritis, intestinal metaplasia (IM) and dysplasia conducting to gastric cancer (GC) [1]. In this process, IM is a crucial lesion, due to its high progression rate to GC (3.77/1000 person-years, in the province of Soria, Spain) [2]. IM is a trans-differentiation process of the gastric epithelium to an intestinal type, mostly induced by *H*.*pylori* infection and expression of the homeobox genes *CDX1* and *CDX2*. It is a protective response against inflammation but it also increases the risk of neoplastic transformation [3].

Intestinal metaplasia has been histologically divided in two types. The complete (CIM, type I, small intestine) is characterized by goblet, absorptive enterocytes and Paneth cells. The incomplete (IIM, types II and III, colonic) is characterized by the presence of goblet and hybrid columnar intermediate cells, absence of enterocytes and rare presence of Paneth cells [2]. Follow-up epidemiologic studies show a higher progression rate to GC of incomplete compared to the complete type of IM [4]. Clinical management guidelines recommend gastroscopy every 3 years when extensive IM is present [5]. However, only a minority of patients with this lesion develop GC [4] and the genes and pathways responsible for this progression are unknown. Therefore, the identification of deregulated genes and molecular processes responsible for this transition is relevant because it could reveal driver genes for tumor progression as well as potential new biomarkers and therapeutic targets.

Gene expression profiling of IM has identified an up-regulation of intestinal differentiation [6,7] and lipid metabolism [6,7,8] genes. However, to our knowledge, there are no published studies comparing samples of IM that progress to gastric cancer along time with those that do not progress. Our hypothesis is that some genes and molecular processes are deregulated in complete and incomplete IM that progress to gastric cancer (CIM-GC and IIM-GC). To test this, we performed expression profiling to compare mRNA from histological subtypes of IM that after a follow-up study had progressed to GC (CIM-GC and IIM-GC) with the mRNA from those IM subtypes that did not progress to GC (CIM-NoGC, IIM-NoGC). Normal gastric mucosa was also compared to IM subtypes that did not progress to GC (IM-NoGC).

## Materials and methods

### Patients and samples

Samples were obtained from two Spanish follow-up studies of gastric carcinogenesis. One was performed in the province of Soria [2] and the other was a multicenter study including 9 Spanish hospitals [9]. Furthermore, samples from the Gregorio Marañón´s hospital, in Madrid, were also included. Patients diagnosed at recruitment with CIM and IIM were subjected to a new gastroscopy after a mean follow-up of 12 ±3.4 years.

As previously published, formalin-fixed paraffin-embedded (FFPE) samples at recruitment and at the end of follow-up were diagnosed by histology [2,9]. Briefly, 3–4 biopsies from the antrum, incisura or corpus, were stained with hematoxylin–eosin, Alcian blue–periodic acid Shiff (AB-PAS, pH 2.5) and Giemsa. Additionally, for some IIM samples, some sections were stained with high-iron diamine–Alcian blue to detect sulfated mucins [2]. Complete IM was classified by the presence of brush border cells and goblet cells, but without non-goblet and hybrid columnar ‘‘intermediate” cells. Incomplete IM, which also contains goblet cells, was diagnosed by the presence of its predominant cell type, the hybrid ‘‘intermediate” non-goblet mucous columnar cells. This hybrid mucous cell is easily identified because they show, with the mucino histochemical AB-PAS, a mixed gastric and intestinal phenotype pattern: red for neutral gastric mucins and blue for a combination of intestinal mucins [2].Two pathologists (MLP and JMSA) reviewed the biopsies of all the included cases. Furthermore, when FFPE blocks were cut for RNA extraction (see below) additional hematoxylin-eosin staining was performed at initial and final cuts to confirm that the tissue in between was from the corresponding IM subtype.

All samples used in this study were obtained from projects approved by the Ethics committee of the Biomedical Research Institute of Bellvitge (CEIC HUB-ICO-IDIBELL), as well as those of the hospitals involved in the projects FIS Exp PI030077 (Hospital de Soria), FIS ExpPI10/01089, PI10/01031 and PI10/01203 and the Gregorio Marañón hospital.

### RNA extraction and microarray analysis

Total RNA was extracted from FFPE cuts of gastric biopsies at recruitment. An initial and a final slide for Hematoxylin-Eosin (HE) staining was obtained and evaluated before RNA extraction in order to further confirm the lesion-type and to select those samples that contained at least 50% of IM in all cuts. RNA was obtained from 8 CIM-GC, 6 IIM-GC, 9 CIM-NoGC, 7 IIM-NoGC, and 15 normal gastric mucosae. We selected FFPE blocks of IM (IIM or CIM)-GC patients of similar sex and age as IM (IIM or CIM)-NoCG patients. The characteristics of the patients’ samples are shown in S1 Table.

Total RNA was extracted with Recover All Nucleic Acid for FFPE kit (Ambion, USA). RNA quality was evaluated using NanoDrop 2000 (Thermo Scientific, USA) for quantification and purity check, and using the Bioanalyzer 2100 (Agilent Technologies, USA) for RNA integrity analysis. For microarray analysis, 100ng of input RNA from each sample was processed using the SensationPlus FFPE Amplification and 3'IVT Labeling Kit (Affymetrix, USA) and hybridized to the Almac Xcel array (Affymetrix, USA), specifically optimized for use with degraded FFPE samples.

### Quality control, statistical and bioinformatics analyses

After quality assessment, data normalization was performed by the Robust Multichip Analysis method (Affymetrix^®^ Expression Console^™^ Software) [10] to correct background, apply quantile normalization and summarize all the probes of a transcript in logarithmic base 2. Batch effect was corrected by method Combat [11]. Of the initial 81.804 probe sets, we selected 57.263 above the 30 percentile to eliminate noise produced by probes that do not express enough intensity/signal. A dendrogram representation of the sample hierarchical clustering was constructed using the Euclidean distance and method Complete.

Differentially expressed genes were obtained by moderated t test for microarray data (Limma) [12] and adjusted by False Discovery Rate (FDR) [13]. Three types of comparisons were performed: IIM-GC vs IIM-NoGC, CIM-GC vs CIM-NoGC and IM-NoGC vs Healthy mucosa. In order to exclude potential tumoral processes, in the comparison of IM with healthy mucosa we did not include IM samples that had progressed to GC. Genes were considered differentially expressed if the t-test *p-*value was <0.05 and the fold change (FC)≥2 (up-regulated) or ≤0.5 (down-regulated). Differences between the analyzed groups in clinical and morphological variables of the study patients (age, sex, *H*.*pylori* infection status and anatomical location of the lesion in the stomach) were analyzed by means of the t-test for continuous variables and the Fisher exact test for categorical variables. Significant differences (p<0.05) were only obtained for age between IM-NoGC group and healthy mucosa. In agreement with the age and sex matched selection of FFPE blocks from IM-GC and IM-NoGC patients, no age differences were observed between the different groups of intestinal metaplasia (IIM or CIM) patients. There were also no differences between groups for any of the other variables analyzed. Thereafter, p-values for moderated t test were only age adjusted in the comparisons IIM-GC vs Healthy, IIM-NoGC vs Healthy, CIM-GC vs Healthy, CIM-NoGC vs Healthy, IM-NoGC vs Healthy and IM-GC vs Healthy. Analyses were performed with R (v3.1.1) and Bioconductor [14] packages. Data are accessible in GEO (GSE78523).

We constructed Venn diagrams (http://bioinformatics.psb.ugent.be/webtools/Venn/) to identify the Differentially Expressed Genes (DEGs) that were unique or common to the different IIM and CIM analysis groups (IIM-GC vs IIM-NoGC, IM-GC vs Healthy mucosa and IM-NoGC vs Healthy mucosa; and the same for CIM) and to select DEGs specific for IIM-GC vs IIM-NoGC and IIM-GC vs Healthy comparisons, but absent in IIM-NoGC vs Healthy.

We used the GEO2R tool of Gene expression Omnibus (https://www.ncbi.nlm.nih.gov/geo/) to analyze the deposited GEO data from a similar study (GSE69146) [15] and to compare with our results.

### Gene Set Enrichment Analysis, GSEA

Normalized expression values were loaded to GSEA [16] and the gene set collections c2.all.v5.0 and c3.tft.v5.0 of MSigDB v5.0 [16] were interrogated. c2.all.v5.0 includes gene sets from signaling pathways and genetic or chemical perturbations, while c3.tft.v5.0 contains genes that share a transcription factor binding site in TRANSFAC v7.4. The default parameters were applied with the exception of IIM-GC vs IIM-NoGC where permutations were performed on *Gene set* following methodology recommendations [17]. A gene set was considered significant if the p-value was <0.05 and q-value was <0.25 for CIM-GC vs CIM-NoGC and IM-NoGC vs Healthy. However, for IIM-GC vs IIM-NoGC a gene set was considered significant if the nominal p-value was <0.01 and the q-value was <0.05 to avoid the higher rate of false positives typically encountered when gene sets are used for permutation [18].

We categorized gene sets to molecular processes according to their functions (e.g. COLLER_MYC_TARGETS_UP (oncogenes)). Furthermore, we looked at the intersections between: A) gene sets with enrichment scores (ES) higher than cutoffs (IIM-GC: ES>0.5, IM-NoGC: ES>0.65), B) extreme values of “Rank at max” parameter and C) leading edge genes that are also DEGs [17].

An expression dataset of IM and healthy gastric mucosa [19] deposited in Gene Expression Omnibus (GSE47797) was downloaded and analyzed by GSEA following the previous methodology.

### Ingenuity Pathway Analysis (IPA)

The default Core Analysis of IPA [20] was used to identify biological processes, functions, canonical pathways and molecular networks from differentially expressed genes in the three specified comparisons. This software compares DEGs from an experiment against genes collected in the Ingenuity^®^ Knowledge base. In this way, canonical pathways and molecular networks are identified, providing insight of how DEGs interact [20]. Results with p-value<0.05 were considered significant.

### Validation by RT-qPCR of differentially expressed genes

Validation of 19 DEGs from the microarray was performed by RT-qPCR using the dynamic expression arrays Biomark HD 96x96 (Fluidigm, USA) and UPL probes (Roche, Switzerland). The RT-qPCR protocols (PN 68000116 B3, PN-100-6472 A1, PN 100–5876 B1 and PN 68000130 E1) of Fluidigm were followed. We selected some top up and down-regulated significant genes of interest from Tables 1–3 (S2 Table) and performed duplicated qPCR in CIM-GC (N = 24), CIM-NoGC (N = 33), IIM-GC (N = 11), IIM-NoGC (N = 8) and Healthy (N = 16) samples. Some of these samples were the same as those used for the microarray analysis (S1 Table); the remaining samples were from an independent series (S3 Table).

**Table 1:** Differentially expressed genes with oncogenic functions in the IIM progressing to GC (IIM-GC).

Gene Symbol^a^	Function	Fold Change	Nominal p-value	Associated with gastric carcinogenesis^b^
*IK*	**Antigen Processing**	0.384	0.002	New
*HLA-A*		2.059	0.029	Yes [23]
*HLA-DQB1*		2.17	0.001	Yes [24]
*CD24*		2.249	0.008	Yes [25]
*HLA-DRB4*		2.25	0.024	Yes [24]
*HLA-DQA1*		3.182	0.024	Yes [24]
*HLA-DRB1/3/5*		3.368	0.048	Yes [24]
*HLA-C*		4.417	0.011	Yes [26]
*CXCL14*	**Inflammation**	2.007	0.028	Yes, GC prognosis [27]
*IL1R2*		2.31	0.019	New
*BPIFB1*		2.866	0.009	New
*C1R*	**Complement system**	2.048	0.005	New
*C1QBP*		2.076	0.002	New
*C3*		2.193	0.020	New
*PPIA*	**Chaperones**	0.393	0.027	Yes [28]
*CCT6A*		2.053	0.002	New
*CANX*		2.117	0.027	New
*HSP90AA1*		2.412	0.0001	Yes, GC prognosis [29]
*HSP90AB1*		2.479	0.003	Yes, GC prognosis [29]
*RBBP7*	**Tumor suppression**	0.449	0.001	New
*EIF5B*		2.059	0.004	New
*CAV1*		2.144	0.0005	YES, GC metastasis [30]
*EIF3D*		2.211	0.003	New
*CDKL3*	**Cell cycle regulator**	0.403	0.0001	New
*CP*	**Metabolism of iron**	0.412	0.040	Yes, decreased in GC [31]
*MYOF*	**Angiogenesis**	2.035	0.006	New
*ANP32B*	**Anti-apoptotic factor**	2.155	0.006	New
*NHP2*	**Telomerase**	2.187	0.002	Yes, GC prognosis [32]
*GNL3*	**Stem cell proliferation**	2.218	0.0003	New
*RAN*	**Oncogene**	2.41	0.004	Yes, SNPs associated [33]

^a^ Genes are ordered increasingly according to Fold Changes inside the functional groups.

^b^ It refers to genes previously associated with gastric carcinogenesis by genetic association, expression, proteomic or functional studies. IIM, Incomplete Intestinal Metaplasia. GC, Gastric cancer

**Table 2:** Differentially expressed genes with oncogenic functions in the CIM progressing to GC (CIM-GC).

Gene Symbol^a^	Function	Fold Change	Nominal p-value	Association with gastric carcinogenesis^b^
*IGHG1/IGHM/ IGHV4-31*	**Antigen Processing**	0.493	0.014	YES
*HLA-DRB4*		2.049	0.022	YES [24]
*HLA-DRB1/3/5*		3.475	0.021	YES [24]
*ANAPC5*	**Mitotic factor**	0.461	0.031	New
*IL1R2*	**Inflammation**	0.475	0.017	New
*PRSS1*	**Protein degradation**	0.495	0.033	New
*GP2*	**Innate immunity response**	2.063	0.018	New
*HOXA13*	**Intestinal differentiation**	2.076	0.028	YES [34]
*IGFBP5*	**Cellular proliferation**	2.127	0.004	New
*OLFM4*	**Antiapoptotic factor**	3.332	0.0002	YES [35]

^a^ Genes are ordered increasingly according to Fold Changes inside the functional groups.

^b^ It refers to genes previously associated with gastric carcinogenesis by genetic association, expression, proteomic or functional studies. CIM, Complete Intestinal Metaplasia. GC, Gastric cancer

**Table 3:** Differentially expressed genes representative of molecular processes in the IM not progressing to GC (IM-NoGC).

Gene Symbol^a^	Function^b^	Fold Change	p-value	Adj p-value
*CDX2*	**Intestinal differentiation**	3.133	4.050E-11	2.343E-08
*CDX1*		4.147	3.523E-09	1.187E-06
*HOXB13*	**Intestinal differentiation (New DEGs)**	2.099	2.508E-04	1.187E-02
*HOXB6*		2.884	2.313E-07	4.386E-05
*HOXA13*		3.294	7.134E-05	4.632E-03
*MUC3A*	**Mucins**	13.277	1.677E-16	6.001E-13
*MUC12*		16.382	4.336E-09	1.395E-06
*MUC17*		15.579	1.550E-15	3.773E-12
*MUC2*		24.748	5.216E-25	1.857E-20
*APOA1*	**Lipid Metabolism**	7.146	4.060E-07	6.902E-05
*APOA4*		7.331	2.059E-08	5.385E-06
*MTTP*		23.334	1.176E-14	1.871E-11
*APOB*		35.139	5.209E-15	9.479E-12
*FABP1*		67.381	7.752E-25	1.857E-20
*CYP3A4*	**Xenobiotic Metabolism**	7.304	4.204E-08	1.003E-05
*UGT2A3*		2.741	9.798E-06	9.690E-04
*GSTA1*		3.285	2.794E-04	1.287E-02
*GAST*	**Hipoclorhidria**	6.249	4.603E-10	2.075E-07
*SST*		0.183	1.815E-08	4.789E-06
*SLC46A3*	**Transporters (New process)**	5.553	1.458E-10	7.258E-08
*SLC7A9*		6.007	7.421E-11	4.047E-08
*SLC17A4*		7.240	3.905E-12	3.105E-09
*SLC5A1*		8.238	2.024E-09	7.492E-07
*SLC13A2*		8.412	2.326E-14	3.330E-11
*SLC6A19*		9.009	1.581E-15	3.773E-12
*SLC26A3*		16.877	3.539E-11	2.136E-08
*TRIM15*	**NFKB activation(New DEGs)**	2.256	4.063E-07	6.902E-05
*TRIM31*		2.372	8.101E-06	8.298E-04
*TRIM36*		2.548	3.530E-07	6.125E-05
*TRIM40*		2.677	1.287E-04	7.379E-03
*SNORD116s*	**Expression regulators (New process)**	0.443	1.378E-04	7.675E-03
*TMEM25*	**TMEM function (New process)**	2.768	1.617E-04	8.622E-03
*TMEM139*		3.386	2.229E-10	1.064E-07
*TMEM45B*		2.709	1.380E-09	5.450E-07
*DMBT1*	**Tumor suppressor**	71.951	9.727E-25	1.857E-20

^a^ Genes are ordered increasingly according to Fold Changes inside the functional groups.

^b^ New means that novel DEGs or molecular processes were found. IIM, Incomplete Intestinal Metaplasia. GC, Gastric cancer

*ACTB*, *GAPDH*, *G6PD*, *RPL29 and B2M* genes were considered as potential reference genes due to their lack of significance in the microarray analysis. The selected reference gene was *ACTB*, which was the one that exhibited the lowest significance by ANOVA (p-value = 0.395), when comparing Ct values of IM-GC vs IM-Not CG vs Healthy controls, using the software GraphPad Prism v5.01 [21]. The expression levels of the target genes and their statistical significance were calculated by use of the Bootsratio software [22] after introduction of 2^-ΔCt^ values for cases and controls, where ΔCt = Ct mean(target gene)-Ct mean (*ACTB*). In all cases a p-value<0.05 was considered significant.

## Results

**Fig 1:**
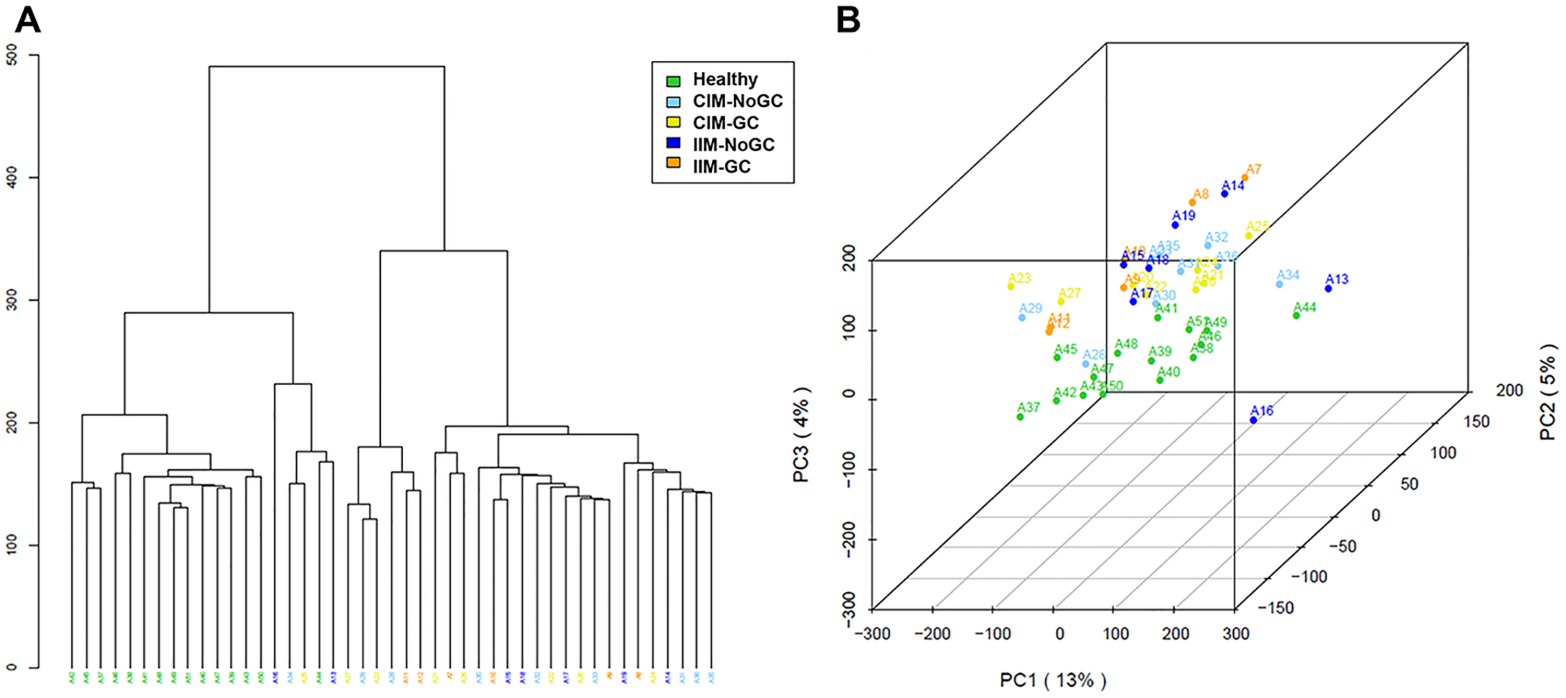
Dendrogram and principal component analysis of the analysed samples. A) Dendrogram showing the hierarchical clustering of the analysed samples. Clustering was based on the overall gene expression values of the studied groups. B) Principal Components Analysis.

**Fig 2:**
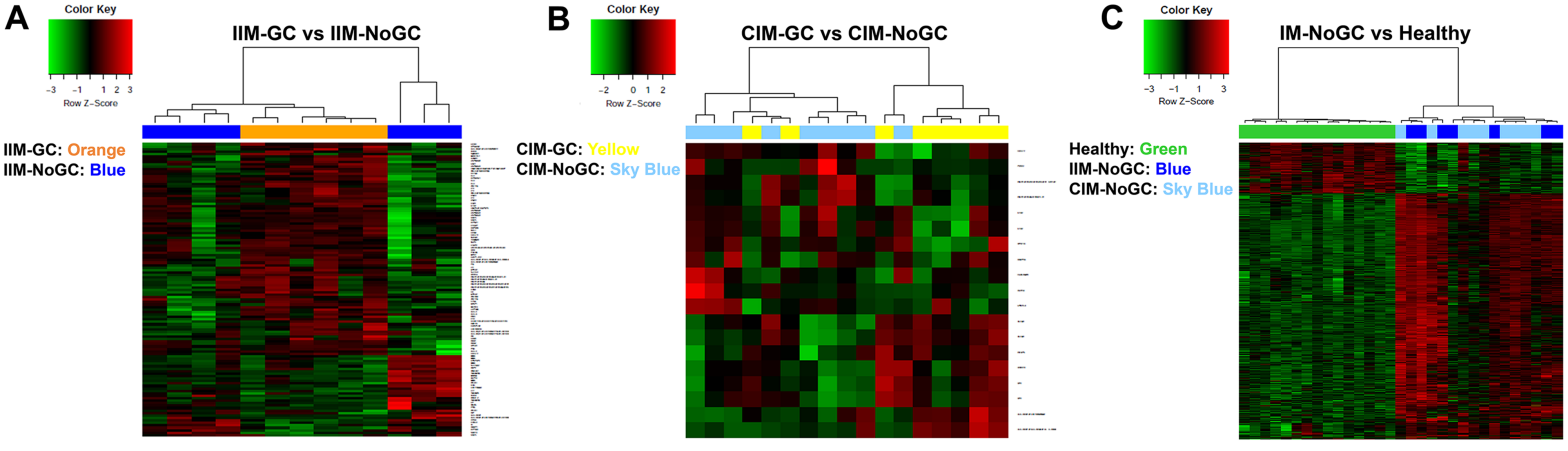
Heat maps of the analyzed groups. A) IIM-GC vs IIM-NoGC, B) CIM-GC vs CIM-NoGC, C) IM-NoGC vs Healthy.

Hierarchical clustering and principal component analysis of the analysed samples based on the gene expression profiles obtained after microarray analysis showed a clear separation between healthy controls and all the IM samples; however, little differences between IM samples that had progressed to GC with respect to those that did not progress were observed (Fig 1). A similar representation of the data was observed in the heatmaps of the compared groups (Fig 2). Differentially expressed genes were observed from the three comparisons, but the differences between IM (either IIM or CIM)-GC and (IIM or CIM)-NoGC were not significant after applying the FDR test.

**IIM-GC vs IIM-NoGC:** 106 genes were identified as differentially expressed, of which 75.4% were over-expressed and 24.6% under-expressed in the IIM-GC (S4 Table).

**CIM-GC vs CIM-NoGC:** 19 genes were differentially expressed, being the majority (N = 11) under-expressed in the CIM-GC (S5 Table).

**Fig 3:**
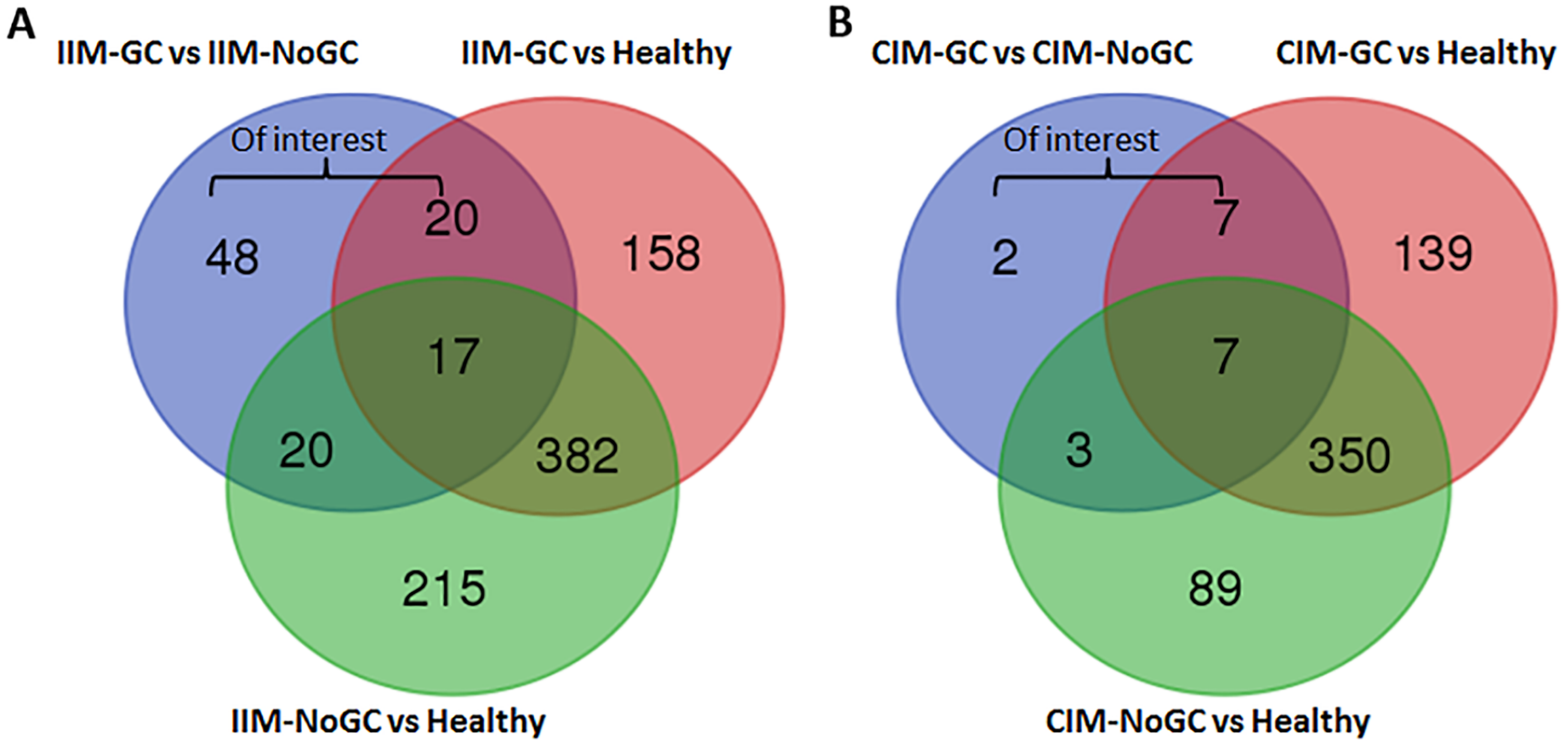
Venn diagrams of the differentially expressed genes in three different comparisons. A, Incomplete intestinal metaplasia. B, Complete intestinal metaplasia. IIM-GC or CIM-GC, incomplete or complete intestinal metaplasia progressing to gastric cancer. IIM-NoGC or CIM-NoGC, incomplete or complete intestinal metaplasia not progressing to gastric cancer. Healthy, healthy gastric mucosa.

To select tumour progression genes specific for the IMs-GC, we compared the DEG of IM (IIM and CIM) that progressed to GC versus those that did not progress with the DEG in the IMs that did not progress versus healthy mucosa and those in the IMs that progressed to GC versus healthy mucosa (Fig 3). Among the genes specific of IIM or CIM progression to GC, there were more deregulated transcripts with oncogenic functions in the IIM-GC (Table 1) than in the CIM-GC (Table 2).

**IIM vs CIM:** Comparison of IIM-GC vs CIM-GC revealed 17 DEGs (p-val<0.05), of which 15 were upregulated and 2 were downregulated (S6 Table), but as in the comparisons of IIM-GC vs IIM-NoGC and CIM-GC vs CIM-NoGC, none of them was significant after FDR adjustment for multiple comparisons (FDR). Nevertheless, it is to note the increased expression in IIM-GC of immunoglobulin and inflammatory gene products, indicating an increased active immunoinflammatory response in IIM-GC. There was also an increased expression in IIM-GC of some key molecules in the gastric function such as pepsinogen, H+/K+ channel generator of the H+ ion for HCl synthesis and gastric intrinsic factor.

Comparison of IIM-NoGC vs CIM-NoGC revealed 16 DEGs (p-val<0.05), of which four DEGs were upregulated and 11 were downregulated (S7 Table); again, none of them was significant after FDR adjustment. Five of these DEGs were the same as in IIM-GC vs CIM-GC but four of them (*CXCL17*, *PGC* and two corresponding to *IGH* genes) were upregulated in IIM-GC and downregulated in IIM-NoGC, while *EBF1* was downregulated in both comparisons.

**IM-NoGC vs Healthy:** We identified 482 DEGs (394 upregulated and 88 downregulated), all of which significant after FDR test. With respect to previous comparisons it is relevant to note the higher number of both DEGs and expression levels (FC>4 N = 92) and that they are all significant after FDR (S8 Table). DEGs contributing to molecular processes previously reported in IM were confirmed and new genes contributing to these processes as well as to new processes were also identified (Table 3).

### Gene Set Enrichment Analysis (GSEA)

**Fig 4:**
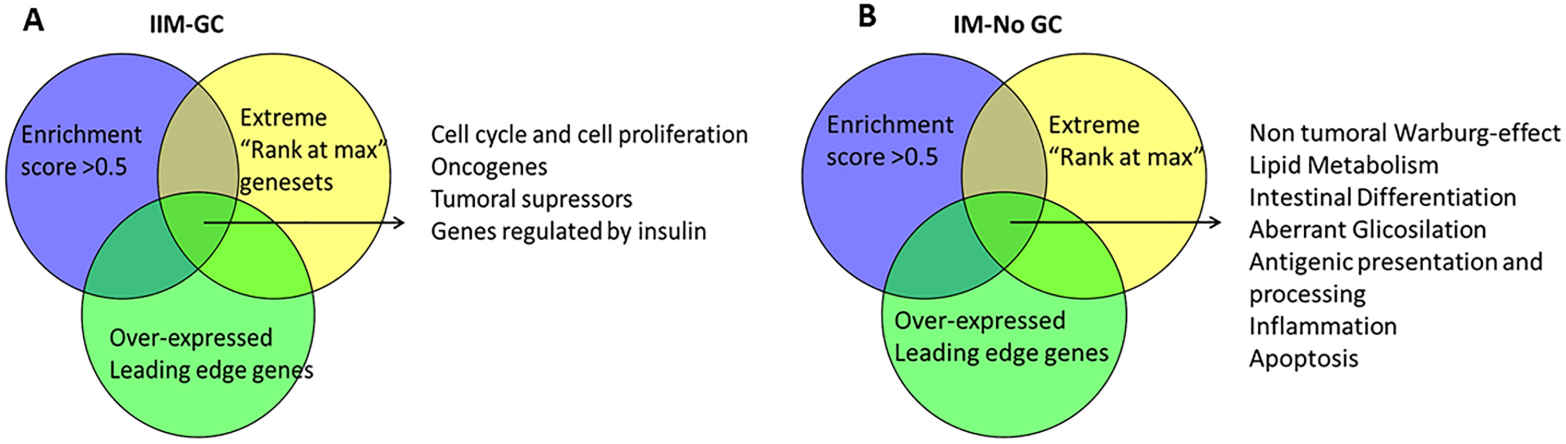
Venn´s diagram to select the most relevant molecular processes after GSEA analyses. A, Incomplete intestinal metaplasia progressing to gastric cancer (IIM-GC). B, Both types of intestinal metaplasia not progressing to gastric cancer (IM-NoGC).

From the c2all.v5 gene set collection we obtained 144 gene sets significantly over-expressed in the IIM-GC. From the c3tft.v5 collection we obtained 19 overexpressed gene sets, all composed by E2F translation initiation factors. The S9 Table indicates the number of significant gene sets per molecular process after categorization according to their function. To select the most relevant molecular processes represented by these gene sets, cut-offs to *Rank at max* and *ES* parameters were applied and up-regulated leading edge genes were identified (S11 and S12 Tables). Comparison of these results with those of the gene sets with enrichment scores above 0.5 indicated that activation of cell cycle and cell proliferation, oncogenes, tumor suppressors and insulin regulated genes were important processes in the progression of IIM to GC (Fig 4A).

For the CIM-GC group we only obtained two significant gene sets (GAZDA_DIAMOND_BLACKFAN_ANEMIA_ERYTHROID_UP, REACTOME NETRIN1_SIGNALING) without any apparent relationship with gastric carcinogenesis.

When both IM subtypes that do no progress to GC were grouped and compared with healthy mucosa, 120 gene sets grouped in different molecular processes were over-expressed in IM-NoGC (S10 Table). There were 12 over-expressed gene sets from the c3.tft.v5.0 catalogue composed by transcription factors HNF1/4 and GATA1/6, which cooperate to induce intestinal genes [3].

When we compared the relevant molecular processes in IM-NoGC, following identical methodology as indicated for IIM-GC (S13 and S14 Tables), the most important processes were found to be Warburg effect, lipid metabolism, intestinal differentiation, aberrant glycosylation, antigen processing, inflammation and apoptosis (Fig 4B).

### Ingenuity Pathway Analysis (IPA)

The IPA analysis showed that, unlike to the GSEA, there were several up-regulated pathways common to the IIM-GC and CIM-GC such as antigen presentation, signaling by TNFRSF4, communication between innate and adaptive cells, maturation of dendritic cells and the possible role of other homeobox genes such as *HOXC11* in the development of the intestinal differentiation. Due to the small number of differentially expressed genes in CIM-GC (Table 2), pathways over-expressed in this group were composed by a small number of genes and of lower statistical significance, when compared with the IIM-GC group (Table 4). Regarding IM-NoGC, IPA confirmed the GSEA results for metabolism of lipids and xenobiotics and a previously observed deregulation of the thyroid hormone metabolism. A new process of melatonin degradation was also identified (Table 4).

**Table 4:** Over-expressed canonical pathways and other information from Ingenuity Pathway Analysis.

Group	Canonical pathway	p-value	*Upstream* Regulators	Diseases	Cellular and molecular functions
IIM-GC	Antigen presentation	3.15E-10	HOXC11 RAD21 EBI3 NLRC5 SMC3	-Immunological -Connective Tissue -Inflammatory -Muscular and Skeletal -Development	-Cell proliferation -Cell death and survival -Cellular movement -Cellular development -Cell-cell interaction and signaling
IIM-GC	TNFRSF4 (OX40) signaling	3.27E-09			
IIM-GC	Thyroid autoimmune disease	1.66E-08			
IIM-GC	Development of B cells	1.51E-07			
IIM-GC	Maturation of dendritic cells	1.26E-06			
IIM-GC	Phagosome maturation	1.59E-06			
IIM-GC	Communication between innate and adaptive cells	1.62E-06			
CIM-GC	Communication between innate and adaptive cells	3.14E-05	LGALS3 LHCGR MYBL2 mir-296 CHI3L1	-Endocrine -Gastrointestinal -Immunological -Inflammatory -Development	-Cell-cell interaction and signaling -Cellular movement -Cell death and survival -Cell cycle - Cellular assembly and organization
CIM-GC	Maturation of dendritic cells	3.22E-04			
CIM-GC	Antigen presentation	3.98E-04			
CIM-GC	TNFRSF4 (OX40) signaling	7.49E-04			
IM-NoGC	Activation of FXR/RXRG	1.42E-08	HNF4A HNRNPA2B1 HNF1A CTNBB1 SREBF1	-Endocrine -Metabolism -Gastrointestinal -Hepatic -Cardiovascular	-Cellular movement -Death and cell survival -Lipid metabolism -Molecular transport -Biochemistry of small molecules, drugs and amino acids
IM-NoGC	Activation of PXR/RXRG	1.34E-07			
IM-NoGC	Inhibition of RXRG by LPS/IL1	2.24E-05			
IM-NoGC	Development of B cells	7.76E-05			
IM-NoGC	Degradation of sucrose	4,30E-05			
IM-NoGC	Metabolism of thyroid hormone	0.0004			
IM-NoGC	Hematopoiesis of stem cells	0.0005			
IM-NoGC	Activation of LXR/RXR	0.0006			
IM-NoGC	Degradation of melatonin (New)	0.0013			
IM-NoGC	Glycolysis I	0.0039			
IM-NoGC	Xenobiotic Metabolism Signaling	0.0052			

The molecular network with highest score in the IIM-GC was found to be composed of effectors of the immune response such as HLA class I (HLA-A, HLA-C) and II (DQB1, DRB1) molecules, the cytokine receptor IL1R2, immunoglobulins, and oncogenic molecules such as the chaperones CANX and HSP90AA1 and the tumor suppressor CAV1 (S1 Fig). There was not a high score molecular network in CIM-GC. The molecular network with the highest score in the IM-NoGC includes the processes of lipid metabolism, molecular transport and biochemistry of small molecules (S2 Fig).

### Validation of DEGS from microarray results by RT-qPCR

**Fig 5:**
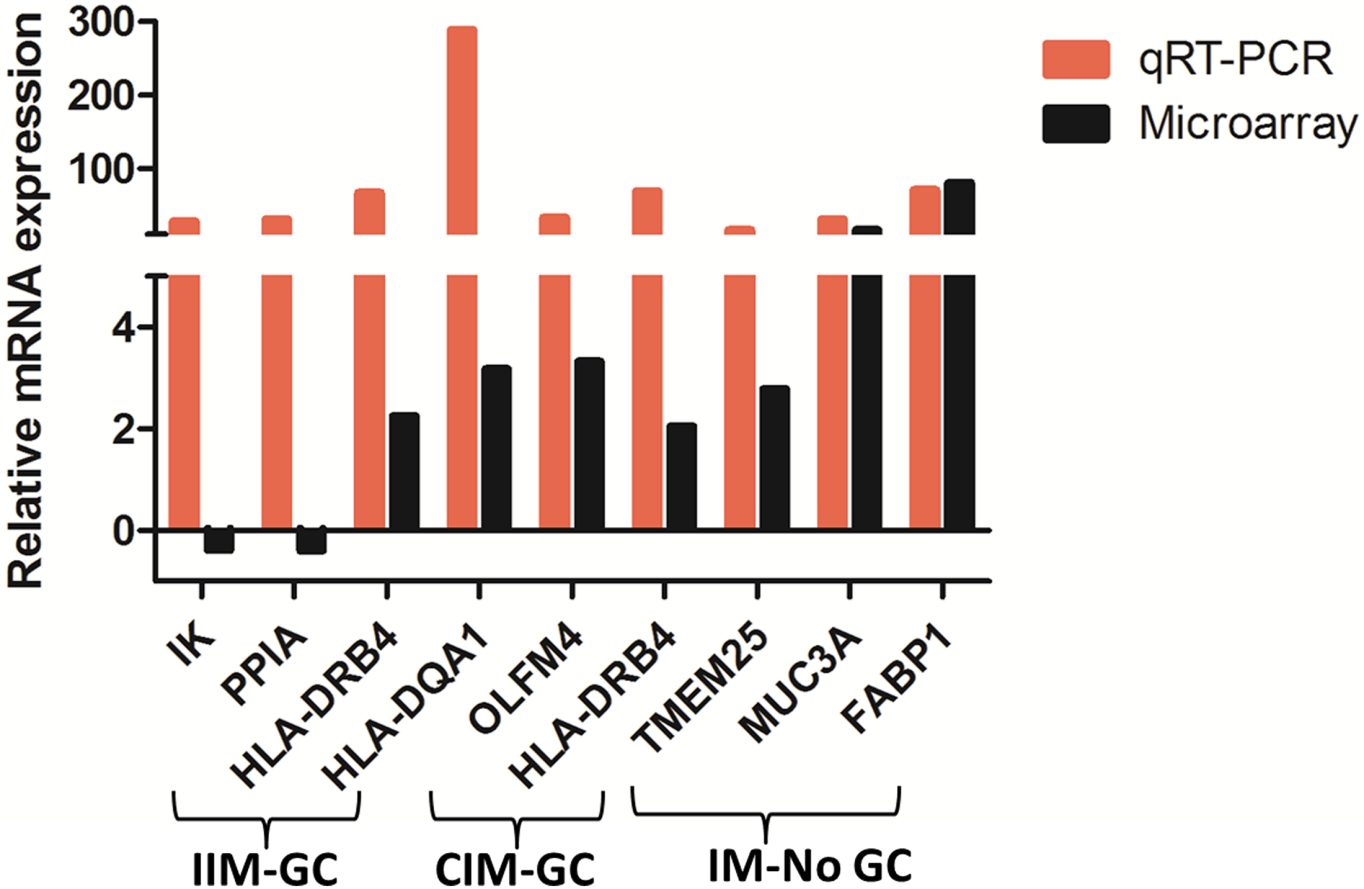
Validation by RT-qPCR of some differentially expressed genes obtained in the microarray analysis. Comparison of the expression level (fold change) by microarray analysis and by RT-qPCR of significant differentially expressed genes in the IIM-CG versus IIM-NoGC, CIM-GC versus CIM-NoGC and IM-NoGC versus healthy gastric mucosa.

RT-qPCR was used to validate selected DEGs obtained in the IIM-GC (N = 7), CIM-GC (N = 4) and IM-NoGC (N = 11) groups (S2 Table). Only 51% of reactions passed the quality control defined by the Fluidigm software, probably reflecting RNA degradation. Of the 19 DEGs selected from the microarray, 9 were also differentially expressed in the qPCR, but only in 7 of them (36.84% of total DEGs) the difference in expression had the same direction (Fig 5). One of the validated genes both in CIM-GC and IMM-GC is *HLA-DRB4*, which is in agreement with the fact that antigen presentation is a molecular process common to the progression of both IM subtypes in the GSEA and IPA analyses. Also, the highest up-regulated gene in CIM-GC, *OLFM4*, was validated.

## Discussion

As far as we know, this is the first study on differential gene expression between IM subtypes that have progressed to gastric cancer and those who have not progressed, after a several years follow-up period. Our results from gene expression and pathway analysis indicate that IM-NoGC is very different from healthy gastric mucosa but the differences between IM subtypes that progress to GC and those that do not progress are smaller and of lower magnitude. Despite this general finding, we have identified DEGs and molecular processes involved in the progression from IM subtypes to gastric cancer. Furthermore, we have confirmed previously reported molecular processes that distinguish IM from healthy gastric mucosa and have identified new transcripts contributing to these processes.

Although expression differences were not found significant after multiple corrections when IIM-GC was compared to IIM-NoGC, some of the DEGs have been previously associated with gastric carcinogenesis. There are several up-regulated transcripts reflecting the inflammatory process induced by *H*. *pylori* infection, such as *HLA-A*, *HLA-C* [26], *HLA-DRB1/3/5*, *HLA-DQA1/DQB1* [24, 36]. It is interesting to note that *HLA-DRB* is up-regulated in both IIM-GC and CIM-GC, that *HLA-DRB4* overexpression was confirmed by RT-qPCR and that antigen presentation also is a common pathway in both IM-subtypes progressing to GC, altogether being in agreement with the immune response as an important process in the progression of precursor lesions to GC [37]. There are several up-regulated inflammatory transcripts (*CXCL14*, *IL1R2*, *BPIFB1)* and also from the complement system (*C1R*, *C1QBP*, *C3*) which increase inflammation and phagocytosis of bacteria, respectively. Regarding this, other complement components such as *C1S*, *C1QR1* and *CD55* have been found over-expressed in GC [38]. Several chaperones responsible for proper oncoproteins folding were found overexpressed, such as *CCT6A*, *CANX*, *HSP90AA1* and *HSP90AB1*. Interestingly, over-expression of *HSP90AA1* and *HSP90AB1* has been associated with poor prognosis in GC [29]. The regulators of the tumor suppressor *RB1*, *EIF3D* and *EIF5B* were also found over-expressed, as well as *NHP2*, a telomerase whose high expression in GC is correlated with poor clinical prognosis [32]. A member of RAS oncogenes called RAN, with genetic polymorphisms associated with of GC risk [33], was also found overexpressed. Novel over-expressed genes not previously associated with gastric carcinogenesis are *MYOF*, an angiogenesis regulator [39], and *GNL3*, that interacts with TP53 and may be involved in carcinogenesis because of its role in cancer and stem cells proliferation [40].

Apart from the above mentioned overexpression of *HLA-DRB*, in the CIM-GC group there were fewer significant transcripts than in IIM-GC. Furthermore, it is to note the increased expression in IIM-GC of immunoglobulin (*IGHG*, *IGHM*) and inflammatory (*CXCL17*) gene products, indicating an increased active immunoinflammatory response in IIM-GC when compared to CIM-GC. All these results are in agreement with the increased progression risk to GC of IIM compared with CIM [4]. It is likely that oncogenic pathways only become activated after CIM progresses to IIM along the Correa´s cascade. Among the over-expressed genes with oncogenic functions in CIM-GC there are *OLFM4* (olfactomedin 4), whose overexpression was validated by RT-qPCR, and *IGFBP5*, an anti-apoptotic and a cell proliferation factor, respectively. OLFM4 is increased in early stages of gastric carcinogenesis and a prognostic marker for advanced GC [35]. OLFM4 has also been found to promote tumor growth in pancreatic cancer [41], altogether suggesting that it could be an early factor of gastric tumor progression from CIM.

Confirmation of our main gene expression results through immunohistochemistry would be the best proof of their validity. Unfortunately, we had no more FFPE sections for further immunohistochemical staining. Therefore, to further validate our results by comparison with other available datasets from similar studies, we have searched PubMed and the Gene Expression Omnibus (GEO) databases for microarray studies exploring the same or similar progression phenotype. We found a recent study (GEO accession: GSE69144) that used microarray analysis to determine gene expression changes in the progression along the gastric precancerous cascade of gastric lesions, in *H*.*pylori* infected subjects followed for a period of 6 years [15]. However, comparison of this study with ours is difficult because of clear differences in study design and microarray platform used. While this study was designed for the identification of changes in gene expression after lesion progression or regression, our study was designed for the identification of biomarkers of progression to GC at baseline samples. Furthermore, in our study we analyzed a high-density transcriptome based microarray containing 97,000 transcripts, while Garay et al. used a Cancer Panel Array restricted to 502 genes. Therefore, only two of the differentially expressed genes of interest in IM progressing to GC in our study (Tables 1 and 2) were also present in the GSE69144 dataset: *CAV1* and *IGFBP5*. Comparison with the GEO2R tool of those baseline samples of the Garay study that had progressed with those that did not change revealed that *IGFBP5* is found similarly overexpressed as in the comparison of CIM-GC vs CIM-NoGC in our study. This gene has also been reported to be up-regulated in gastric cancer [42]. *CAV1* was not significant in Garay’s study.

To characterize the transcriptional profile of IM in comparison to healthy gastric mucosa, we analyzed samples of both histological subtypes that do not progress to GC because these are more common and also to exclude tumorigenic processes from the analysis. Unlike previous comparisons, DEGs in IM were significant after multiple comparisons and exhibited greater expression differences. There are many DEGs previously found up-regulated in IM, such as *CDX2*, *KRT20*, *MUC13*, *OLFM4*, *REG4* [43], *FABP1*, *MEP1B*, *SI*, *SLC6A19* [44], *CDX1*, *MTTP*, *CEACAM6* [6], *APOA1*, *APOA4*, *APOB*, *CDH17*, *CLDN3*, *HNF4A*, *VIL1* [7]. Their functions show that intestinal differentiation, metabolism of lipids and xenobiotics, and hypoclorhidria, characterize the IM. New DEGs identified in the IM-NoGC group reveal the role of other homeobox genes (HOXA13/B6/C) in the intestinal differentiation, transporters of the solute carrier family (SLC), NFKB activators (TRIM), small nucleolar RNAs (SNORD116) and TMEM proteins of unknown function. The highest over-expressed gene is DMBT1 (S6 Table), a GC tumor suppressor and immunohistochemical marker of IM [45]. It may be speculated that its high expression could be an early defense mechanism against a potential oncogenic deregulation along time.

The results from GSEA also showed a difference between IIM-GC and CIM-GC; in the first group there were many over-expressed gene sets with oncogenic functions, while none was identified in the CIM-GC. The most relevant molecular processes in IIM-GC are antigen processing, inflammation, activation of cell cycle and cell proliferation, oncogenes and tumor suppressors. Other published molecular processes support these differences between the IM subtypes, indicating that IIM is a more advanced state than CIM along the Correa´s cascade [1]. Thus, when compared to CIM, IIM has decreased expression of *CDX2* [46], less activation of Sonic Hedgehog pathway [47], higher microsatellite instability [48], increased telomerase activity [49] and increased intracellular localization of *H*.*pylori* [50].

Regarding the IM-NoGC group, among the most relevant processes we found the Warburg effect, which consists that in tumoral processes glycolysis is increased with respect to oxidative phosphorylation [51] [52]. In IM-NoGC there is an over-expression of glucose transporters SLC2A5, SLC5A9, the glycolytic enzyme hexokinase and glucagon, a promoter of gluconeogenesis. The final product of glycolysis, lactate, activates the hypoxia inducible factors HIF1A and HIF2A, which induce glycolytic genes [52], and in this regard there are two over-expressed HIF1A gene sets. Warburg effect is universal in normal cells under active proliferation to protect them from genomic damage [52], and this could be its role in IM-NoGC. As already indicated by the DEGs analysis, other relevant processes in IM-NoGC were found to be lipid metabolism and the O-glycosylation of mucins. During active cell proliferation, biological membranes with high lipid contents, are synthesized for new cells [53]. Besides, since lipids are used as a gluconeogenic energy source, increased lipid metabolism could be at least in part a consequence of Warburg effect. Mucins are major components of gastric mucus, with high O-glycosylation contributing to mucosa protection from bacterial infections. *H*.*pylori* modulates glycosylation to create a micro-environment favorable to infection. In the IM, aberrant glycosylation of MUC5AC and MUC6 increase their hydrosolubility [54], maybe allowing penetration of *H*.*pylori* in the gastric mucosa and hence increasing inflammation [50].

The consistency of our results is also indicated by the general agreement obtained when we compared by GSEA the expression profile of IM-NoGC with the raw data of a microarray expression dataset of IM and healthy gastric mucosa performed by Hanada *et al* [19] and deposited in GEO. As in our study, major biological processes over-expressed in that study were non tumoral Warburg effect, lipid metabolism, intestinal differentiation, transcription factors HNF1A/4A, inflammation, aberrant protein glycosylation, apoptosis, xenobiotic metabolism and response to genomic damage.

The results from IPA confirmed the findings of previous works and from GSEA about an up-regulation of antigen presentation in both IM subtypes that progress to GC, as well as metabolism of lipids, xenobiotics and dysregulation of thyroid hormone [55] in IM not progressing to GC. New results from this analysis indicate that other transcriptional factors different from CDX1/CDX2, such as HOXC11, could induce intestinal differentiation. HOXC11 is an upstream regulator and other members of this family such asHOXA13, HOXB6/7/13 are over-expressed in IM. Other new up-regulated molecular process in IM not progressing to GC obtained from IPA is melatonin degradation.

The novelties of this study are that we analyzed both metaplasia histological subtypes that progress or not to GC, with a high extension of the lesion (above 75% in most samples) and performed a functional enrichment analysis based on GSEA and IPA. However, the small sample size, particularly in the CIM-GC and IIM-GC groups, is the main limitation of our study since it reduces its power to obtain significant results after correction for multiple comparisons. Small sample size, together with the fact that most (75%) of the samples were reported to be negative for *H*.*pylori* infection, was also the main reason for not performing a stratified analysis by *H*.*pylori* infection. It is nevertheless to note that obtaining appropriate samples for expression analysis of IM subtypes whose progression or not to GC is known from follow-up studies is not an easy task. The results of this study are therefore a starting point for the design of new and larger studies aimed at their validation.

To conclude, our results are the first to indicate that the transcriptional profile of IM subtypes that progress to GC shows small differences in the gene expression levels in comparison with those IM subtypes that do not progress. Antigen presentation and inflammation are upregulated processes common to both IM histological subtypes that progress to GC but the IIM subtype shows a higher number of up-regulated oncogenic DEGs and molecular processes than CIM, which is in agreement with its higher risk of progression to GC.

In agreement with previous reports, the transcriptional profile of IM-NoGC with respect to healthy mucosa evidences a drastic difference in gene expression. Apart from already reported processes such as intestinal differentiation, metabolism of lipids and xenobiotics, new molecular processes observed in this study are non tumoral Warburg effect and melatonin degradation. Newly identified transcripts include TRIM, TMEM, homeobox genes from HOX family, transporter genes and the small nucleolar RNAs, SNORDs116.

## Supporting information

S1 Fig Molecular network with highest score in the IIM-GC comparison, after Ingenuity Pathway Analysis. (TIF)

S2 Fig Molecular network with highest score in the IM-NoGC comparison, after Ingenuity Pathway Analysis. (TIF)

S1 Table Main characteristics of the samples analyzed in the expression microarray. (DOC)

S2 Table Significant genes from the expression microarray selected for validation by qPCR. (DOC)

S3 Table Independent series of samples used in the validation by qPCR. (DOC)

S4 Table Differentially expressed genes in IIM-GC. (DOC)

S5 Table Differentially expressed genes in CIM-GC. (DOC)

S6 Table Differentially expressed genes between IIM-GC and CIM-GC. (DOC)

S7 Table Differentially expressed genes between IIM-NoGC and CIM-NoGC. (DOC)

S8 Table Differentially expressed genes in the IM-NoGC. (DOC)

S9 Table GSEA analysis in IIM-GC from c2all.v5 and c3tft.v5 catalogs. (DOC)

S10 Table GSEA analysis in IM-NoGC from c2all.v5 and c3tft.v5 catalogs. (DOC)

S11 Table Over-expressed gene sets in IIM-GC with extreme values of *Rank at max* parameter. (DOC)

S12 Table Gene sets composed by at least 3 leading edge genes up-regulated in the IIM-GC. (DOC)

S13 Table Over-expressed gene sets in IM-NoGC with extreme values of *Rank at max* parameter. (DOC)

S14 Table Gene sets composed by at least 3 leading edge genes overexpressed in the IM-NoGC. (DOC)
