# Supplementary figures and images for: Gene expression study and pathway analysis of histological subtypes of intestinal metaplasia that progress to gastric cancer
Source: PLoS One. 2017 Apr 25;12(4):e0176043. doi: 10.1371/journal.pone.0176043 (PMC5404762; doi:10.1371/journal.pone.0176043)

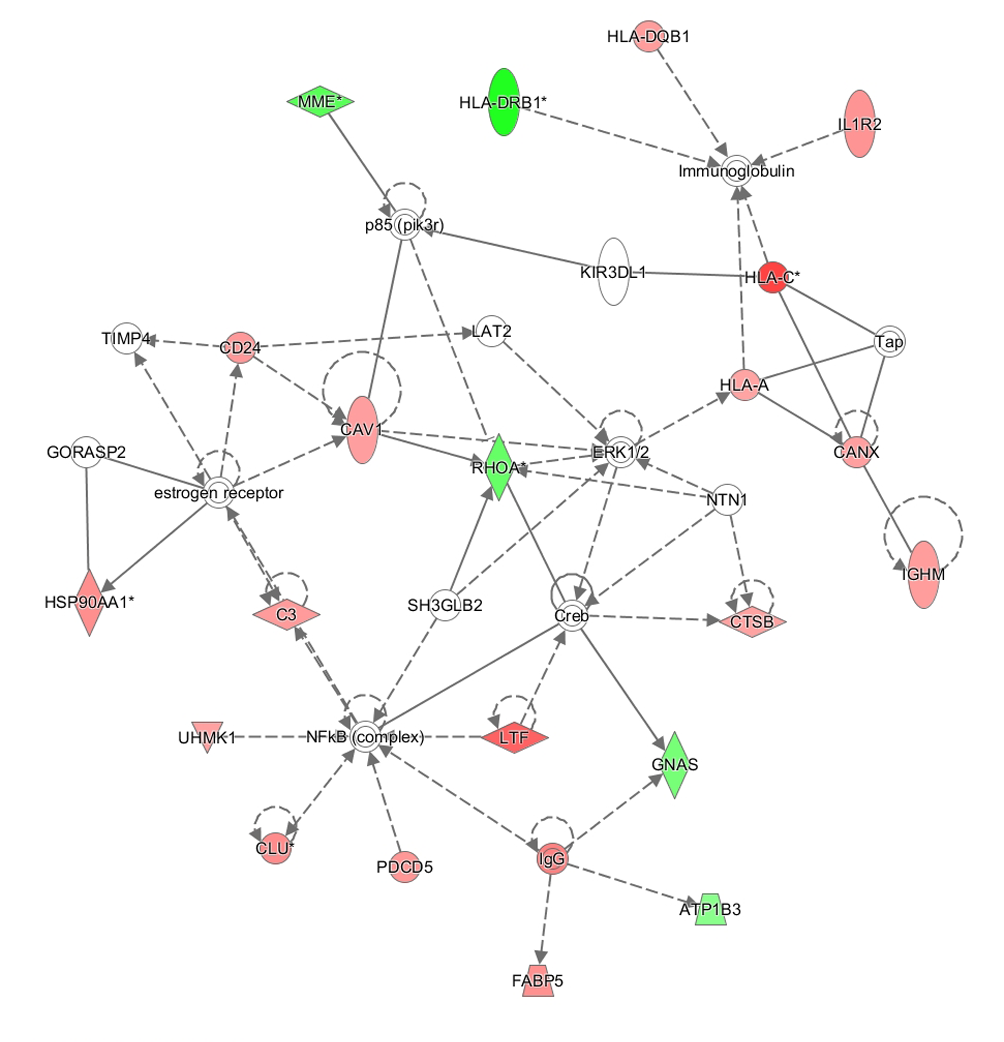

Supplement: S1 Fig — (TIF) [file pone.0176043.s001.tif]

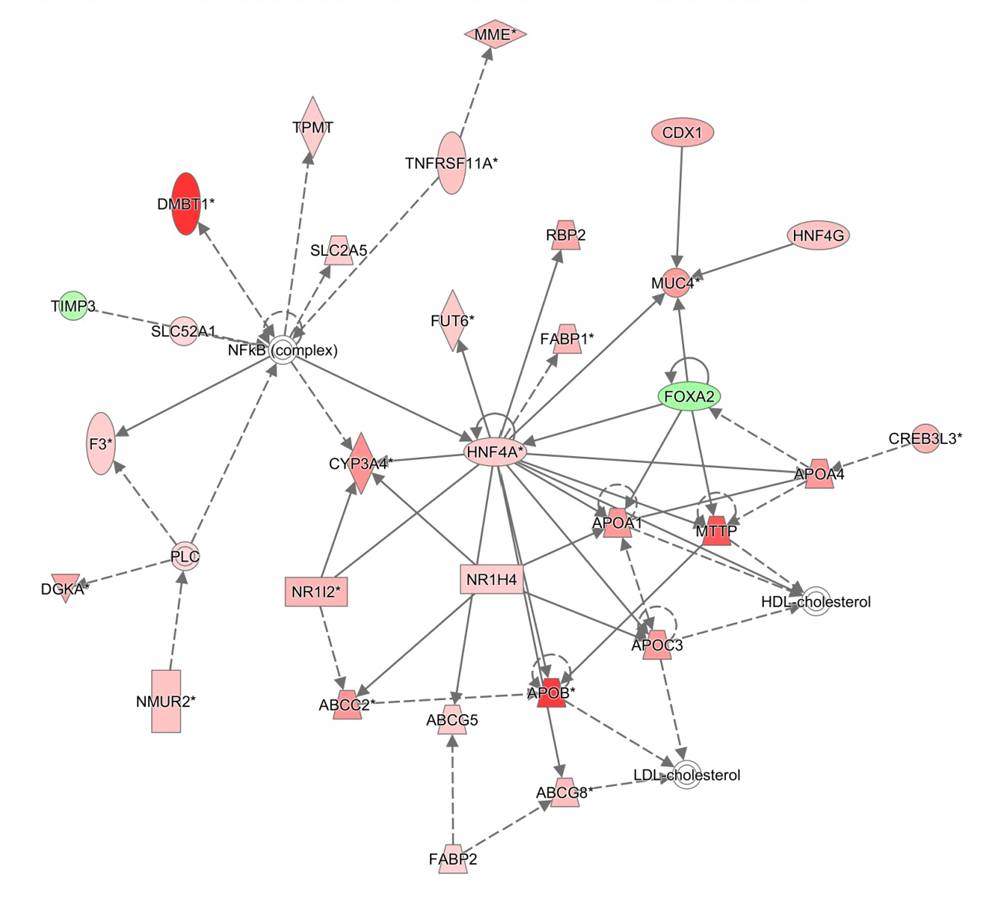

Supplement: S2 Fig — (TIF) [file pone.0176043.s002.tif]
