# Supplementary material for: Gene expression study and pathway analysis of histological subtypes of intestinal metaplasia that progress to gastric cancer
Source: PLoS One. 2017 Apr 25;12(4):e0176043. doi: 10.1371/journal.pone.0176043 (PMC5404762; doi:10.1371/journal.pone.0176043)
Supplement: S1 Table — (DOC) [file pone.0176043.s003.doc]

**S1 Table. Main characteristics of the samples analysed in the expression microarray.**

| **Code** | **Diagnosis at recruitment ^a^** | **Extension at recruitment ^b^** | **Anatomical localization** | **Diagnosis at the end of follow-up** | **Years of follow-up ^c^** | **Estatus ^d^** | ***H.pylori* infection** | **Sex ^e^** | **Age ^f^** | **Source ^g^** |
| --- | --- | --- | --- | --- | --- | --- | --- | --- | --- | --- |
| A7 | IIM | E4 | Antrum | GC intestinal | 10 | IIM-GC | NO | F | 52 | H. Soria |
| A8 | IIM | E4 | Antrum | GC intestinal | 8.4 | IIM-GC | YES | M | 69 | H. Soria |
| A9 | IIM | E3 | Antrum | GC Diffuse | 7.7 | IIM-GC | NO | M | 45 | H.Soria |
| A10 | IIM | E3 | Antrum | GC intestinal | 2 | IIM-GC | NO | M | 73 | H.Soria |
| A11 | IIM | E3 | Antrum | GC intestinal | 1 | IIM-GC | NO | M | 60 | H.Soria |
| A12 | IIM | E3 | Antrum | GC intestinal | 11.8 | IIM-GC | YES | F | 66 | H.Soria |
| A13 | IIM | E4 | Antrum | IIM | 13.4 | IIM-NoGC | NO | M | 53 | H.G. M |
| A14 | IIM | E4 | Antrum | IIM | 15 | IIM-NoGC | NO | M | 64 | H.Soria |
| A15 | IIM | E4 | Antrum | IIM | 11 | IIM-NoGC | NO | F | 64 | H.Soria |
| A16 | IIM | E4 | Antrum | IIM | NA | IIM-NoGC | NO | M | 64 | H.Soria |
| A17 | IIM | E4 | Antrum | IIM | 14 | IIM-NoGC | NO | M | 65 | H.Soria |
| A18 | IIM | E4 | Antrum | IIM | NA | IIM-NoGC | NO | F | 74 | H.Soria |
| A19 | IIM | E4 | Antrum | IIM | NA | IIM-NoGC | NO | M | 71 | H.Soria |
| A20 | CIM | E4 | Antrum | GC no cardias | 2 | CIM-GC | NA | M | 57 | Multicentre |
| A21 | CIM | E4 | Body | GC intestinal | 8 | CIM-GC | YES | M | 57 | Multicentre |
| A22 | CIM | E4 | Antrum | GC intestinal | 1 | CIM-GC | NA | M | 44 | Multicentre |
| A23 | CIM | E4 | Antrum | GC no cardias | 15.37 | CIM-GC | NO | M | 56 | H.G. M |
| A24 | CIM | E4 | Antrum | GC no cardias | 14 | CIM-GC | YES | F | 60 | Multicentre |
| A25 | CIM | E4 | Antrum | GC no cardias | 8.24 | CIM-GC | NO | F | 57 | H.G. M |
| A26 | CIM | E4 | Antrum | GC no cardias | 5.95 | CIM-GC | NO | F | 78 | H.G. M |
| A27 | CIM | E4 | Antrum | GC no cardias | 6.2 | CIM-GC | NO | M | 76 | H.G. M |
| A28 | CIM | E4 | Transitional | CIM | 10.6 | CIM-NoGC | NO | M | 67 | H.G. M |
| A29 | CIM | E4 | Antrum | CIM | 10.5 | CIM-NoGC | YES | M | 63 | H.G. M |
| **Code** | **Diagnosis at recruitment ^a^** | **Extension at recruitment ^b^** | **Anatomical localization** | **Diagnosis at the end of follow-up** | **Years of follow-up ^c^** | **Estatus ^d^** | ***H.pylori* infection** | **Sex ^e^** | **Age ^f^** | **Source ^g^** |
| A30 | CIM | E4 | Transitional | CIM | 12.31 | CIM-NoGC | NO | M | 74 | H.G. M |
| A31 | CIM | E4 | Antrum | CIM | 13 | CIM-NoGC | NO | M | 57 | Multicentre |
| A32 | CIM | E4 | Antrum | IIM | 14 | CIM-NoGC | YES | F | 54 | H.Soria |
| A33 | CIM | E4 | Antrum | IIM | 15 | CIM-NoGC | YES | M | 57 | H.Soria |
| A34 | CIM | E4 | Antrum | CIM | 18 | CIM-NoGC | NO | M | 57 | H.Soria |
| A35 | CIM | E4 | Antrum | CIM | 14 | CIM-NoGC | NO | M | 62 | H.Soria |
| A36 | CIM | E4 | Antrum | CIM | 15 | CIM-NoGC | NO | M | 62 | H.Soria |
| A37 | -------- | -------- | Antrum | -------- | -------- | Healthy | YES | F | 16 | H.Soria |
| A38 | -------- | -------- | Antrum | -------- | -------- | Healthy | NO | M | 42 | H.Soria |
| A39 | -------- | -------- | Antrum | -------- | -------- | Healthy | NO | M | 39 | H.Soria |
| A40 | -------- | -------- | Antrum | -------- | -------- | Healthy | NO | M | 41 | H.Soria |
| A41 | -------- | -------- | Antrum | -------- | -------- | Healthy | NO | F | 72 | H.Soria |
| A42 | -------- | -------- | Antrum | -------- | -------- | Healthy | NO | F | 86 | H.Soria |
| A43 | -------- | -------- | Antrum | -------- | -------- | Healthy | NO | F | 18 | H. Soria |
| A44 | -------- | -------- | Body | -------- | -------- | Healthy | NO | M | 27 | H. Soria |
| A45 | -------- | -------- | Antrum | -------- | -------- | Healthy | NO | M | 68 | H. Soria |
| A46 | -------- | -------- | Antrum | -------- | -------- | Healthy | NO | F | 31 | H. Soria |
| A47 | -------- | -------- | Antrum | -------- | -------- | Healthy | NO | F | 19 | H. Soria |
| A48 | -------- | -------- | Body | -------- | -------- | Healthy | NO | M | 73 | H. Soria |
| A49 | -------- | -------- | Body | -------- | -------- | Healthy | NO | F | 58 | H. Soria |
| A50 | -------- | -------- | Antrum | -------- | -------- | Healthy | NO | F | 11 | H. Soria |
| A51 | -------- | -------- | Body | -------- | -------- | Healthy | YES | M | 69 | H. Soria |

^a^ Histological subtypes of IM (Intestinal Metaplasia). ^b^ Extension of IM in the sample: E3>50%, E4>75%. ^c^ Time interval between recruitment and the end of follow-up. NA, not available. ^d^ CIM/IIM-GC: Sample of CIM (Complete Intestinal Metaplasia) or IIM (Incomplete Intestinal Metaplasia) at recruitment, progressing to GC at the end of follow up. CIM/IIM-Not GC: Samples of CIM or IIM at recruitment who do not progress to GC at follow-up. Healthy: no gastric inflamation, no gastric cancer precursor lesion (atrophy or intestinal metaplasia). ^e^ F: Female, M: Male. ^f^ Age at the time the sample was collected. ^g^  Study from which the samples were obtained, HGM means Gregorio Marañón Hospital.
