# Supplementary material for: Gene expression study and pathway analysis of histological subtypes of intestinal metaplasia that progress to gastric cancer
Source: PLoS One. 2017 Apr 25;12(4):e0176043. doi: 10.1371/journal.pone.0176043 (PMC5404762; doi:10.1371/journal.pone.0176043)
Supplement: S2 Table — (DOC) [file pone.0176043.s004.doc]

**S2 Table.** Significant genes from the expression microarray to be validated by qPCR.

| **Gene** | **Transcript number** | **Comparison Group** | **UPL probe** | **Amplicon size (bp)** | **Primer sequences** |
| --- | --- | --- | --- | --- | --- |
| *ACTB* | NM_001101.3 | Reference genes | 64 | 97 | ccaacgcgcagaagatga and ccagagggctacagggatag  ccagagggctacagggatag |
| *B2M* | NM_004048.2 | Reference genes | 42 | 86 | ttctggcctggaggctatc and tcaggaaatttgactttccattc |
| *G6PD* | NM_000402.4, NM_001042351.2 | Reference genes | 22 | 91 | ctgcagatgctgtgtctggt and tgcatttcaacaccttgacc |
| *GAPDH* | NM_002046.3 | Reference genes | 24 | 70 | cctcctcctaagatggtgtctg and gacgcatggctccaaatc |
| *RPL29* | NM_000992.2 | Reference genes | 53 | 81 | caggctcccaaagctacc and gcaccagtccttctgtcctc |
| *HLA-C* | NM_001243042.1, NM_002117.5 | IIM-GC | 32 | 116 | accgcgactcacattctcc and ggcgtgtgcaaatacctcat |
| *HLA-DQA1* | NM_002122.3 | IIM-GC | 68 | 101 | accaagggccattgtgaat and aatgcggccagagaatagtg |
| *HLA-DRB3* | NM_022555.3 | IIM-GC, CIM-GC | 41 | 113 | gggctgttcatctacttcagga and caaagctggggcagaagat |
| *HLA-DRB4* | NM_021983.4 | IIM-GC, CIM-GC | 41 | 98 | gggacagggctgttcatcta and ccttgaatgtggtcatctgc |
| *IK* | NM_006083.3 | IIM-GC | 11 | 103 | agctgacccagatcctttca and tcagcctcaggaggtttctt |
| *IL1R2* | NM_173343.1, NM_004633.3 | IIM-GC, CIM-GC | 72 | 89 | cacatagagaggccctaccc and ggcacttcaatgtagttctcattatt |
| *PPIA* | NM_021130.3 | IIM-GC | 48 | 97 | atgctggacccaacacaaat and tctttcactttgccaaacacc |
| *OLFM4* | NM_006418.4 | CIM-GC | 24 | 74 | atcaaaacacccctgtgctc and gctgatgttcaccacaccac |
| *APOB* | NM_000384.2 | IM-NoGC | 55 | 78 | gagcacttttctaaatggaacttctac and ctcagttttgaatatggtgagttttt |
| *CDX1* | NM_001804.2 | IM-NoGC | 70 | 76 | agcccctagcagtggatg and tgtccttggtcgcagtcttac |
| *CDX2* | NM_001265.3 | IM-NoGC | 34 | 82 | atcaccatcgcgaggaaag and tggcgttctgaaaccagatt |
| *CYP3A4* | NM_001202855.2, NM_017460.5 | IM-NoGC | 2 | 96 | gatggctctcatcccagactt and agtccatgtgaatgggttcc |
| *DMBT1* | NM_004406.2,NM_007329.2, NM_017579.2 | IM-NoGC | 21 | 77 | accaacttacgcgcattgac and gcacacctgtcacctccatt |
| *FABP1* | NM_001443.2 | IM-NoGC | 78 | 90 | tgatccaaaagcaattcagc and caccttccaactgaaccactg |
| *MUC12* | NM_001164462.1 | IM-NoGC | 72 | 68 | cctggaaaccttagcaccag and gacagagccattgttttccat |
| *MUC17* | NM_001040105.1 | IM-NoGC | 17 | 76 | ggggtgaacatcacaaagcta and gtgtgtacttggttcttaggaggac |
| *MUC3A* | ENST00000319509, ENST00000422757, ENST00000414964 | IM-NoGC | 18 | 102 | gtggagatcctgtccctgag and cacctgctcatactgcctctc |
| *SLC26A3* | NM_000111.2 | IM-NoGC | 2 | 74 | ccatcatgctgctgattgtc and agctgccaggagcgactt |
| *TMEM25* | NM_001144034.1, NM_001144035.1, NM_001144036.1, NM_001144037.1, NM_001144038.1, NM_032780.3 | IM-NoGC | 79 | 109 | gccctctgtcatccttaatgt and caccagggcaaacaggac |
