# Supplementary material for: Gene expression study and pathway analysis of histological subtypes of intestinal metaplasia that progress to gastric cancer
Source: PLoS One. 2017 Apr 25;12(4):e0176043. doi: 10.1371/journal.pone.0176043 (PMC5404762; doi:10.1371/journal.pone.0176043)
Supplement: S3 Table — (DOC) [file pone.0176043.s005.doc]

**S3 Table. Independent series of samples used in the validation by qPCR.**

| **Code** | **Diagnosis at recruitment ^a^** | **Extension at recruitment ^b^** | **Anatomical localization** | **Estatus ^c^** | ***H. pylori* infection** | **Sex** | **Age at recruitment** | **Source ^d^** |
| --- | --- | --- | --- | --- | --- | --- | --- | --- |
| A52 | B-G1 F-G1 | B-E2 F-E2 | Antrum | CIM-NoGC | NA | Male | 61 | Multicentre |
| A53 | B-G3 F-G4 | B-E1 F-E4 | Antrum | IIM-GC | NO | Female | 80 | Soria |
| A54 | B-G1 F-G1 | B-E2 F-E1 | Antrum | CIM-GC | YES | Male | 49 | Multicentre |
| A55 | B-G1 F-G1 | B-E1 F-E1 | Body | CIM-NoGC | YES | Female | 47 | Multicentre |
| A56 | B-G2 F-G2 | B-E4 F-E4 | Antrum | CIM-NoGC | YES | Female | 68 | Multicentre |
| A57 | B-G2 F-G2 | B-E2 F-E2 | Incisura | CIM-NoGC | NO | Male | 69 | Multicentre |
| A58 | B-G2 F-G2 | B-E2 F-E2 | Antrum | CIM-NoGC | NA | Male | 57 | Multicentre |
| A59 | B-G2 F-G2 | B-E3 F-E3 | Antrum | CIM-NoGC | YES | Female | 54 | Multicentre |
| A60 | B-G1 F-G1 | B-E2 F-E2 | Body | CIM-NoGC | NO | Female | 54 | Multicentre |
| A61 | B-G1 F-G1 | B-E1 F-E1 | Antrum | CIM-NoGC | NO | Male | 61 | Multicentre |
| A62 | B-G2 F-G2 | B-E4 F-E3 | Antrum | CIM-NoGC | NO | Female | 49 | Multicentre |
| A63 | B-G1 F-G1 | B-E1 F-E1 | Antrum | CIM-NoGC | YES | Male | 49 | Multicentre |
| A65 | B-G1 F-G1 | B-E1 F-E1 | Antrum | CIM-NoGC | YES | Female | 46 | Multicentre |
| A66 | B-G1 F-G1 | B-E1 F-E1 | Antrum | CIM-NoGC | YES | Male | 35 | Multicentre |
| A67 | B-G1 F-G1 | B-E2 F-E2 | Antrum | CIM-NoGC | NO | Male | 55 | Multicentre |
| A68 | B-G1 B-G1 | B-E2 F-E1 | Antrum | CIM-NoGC | YES | Female | 44 | Multicentre |
| A69 | B-G1 F-G1 | B-E1 F-E1 | Body | CIM-NoGC | NO | Male | 53 | Multicentre |
| A70 | B-G1 F-G1 | B-E2 F-E1 | Antrum | CIM-NoGC | NO | Male | 44 | Multicentre |
| A72 | B-G1 F-G3 | B-E1 F-E3 | Body | IIM-NoGC | NO | Female | 52 | Multicentre |
| A74 | B-G1 F-G1 | I -E1 F-E1 | Body | CIM-GC | YES | Male | 82 | Soria |
| A75 | B-G1 F-G1 | B-E3 F-E2 | Incisura | CIM-GC | NO | Female | 75 | Soria |
| A76 | B-G1 F-G1 | B-E1 F-E1 | Antrum | CIM-NoGC | NO | Male | 67 | Soria |
| A77 | B-G1 F-G1 | B-E2 F-E2 | Body | CIM-GC | NO | Male | 49 | Multicentre |
| A78 | B-G1 F-G1 | B-E2 F-E2 | Antrum | CIM-NoGC | YES | Female | 64 | Soria |
| A79 | B-G1 F-G1 | B-E1 F-E2 | Antrum | CIM-NoGC | NA | Female | 53 | Multicentre |
| A80 | B-G1 F-G2 | B-E3 F-E3 | Body | CIM-NoGC | NA | Female | 49 | Multicentre |
| **Code** | **Diagnostic at recruitment ^a^** | **Extension at recruitment ^b^** | **Anatomical localization** | **Estatus ^c^** | ***H. pylori* infection** | **Sex** | **Age at recruitment** | **Source ^d^** |
| A81 | B-G1 F-G1 | B-E3 F-E3 | Body | CIM-NoGC | NA | Male | 55 | Multicentre |
| A82 | B-G3 F-G3 | B-E3 F-E2 | Antrum | IIM-GC | NO | Female | 81 | La Princesa |
| A83 | B-G1 F-G1 | B-E1 F-E1 | Antrum | CIM-GC | NO | Male | 60 | La Princesa |
| A84 | B-G1 F-G1 | B-E1 F-E1 | Antrum | CIM-GC | NO | Female | 53 | La Princesa |
| A85 | B-G1 F-G1 | B-E1 F-E1 | Body | CIM-GC | YES | Male | 63 | Multicentre |
| A86 | B-G1 F-G1 | B-E1 F-E1 | Body | CIM-GC | YES | Male | 63 | Multicentre |
| A87 | B-G1 F-G1 | B-E3 F-E3 | Body | CIM-GC | NO | Male | 52 | Multicentre |
| A88 | B-G1 F-G1 | B-E1 F-E1 | Incisura | CIM-GC | YES | Male | 54 | Multicentre |
| A89 | B-G1 F-G1 | B-E3 F-E3 | Antrum | CIM-GC | NO | Female | 69 | Multicentre |
| A90 | B-G1 F-G1 | B-E1 F-E1 | Antrum | CIM-GC | NO | Female | 58 | Multicentre |
| A91 | B-G1 F-G1 | B-E3 F-E3 | Antrum | CIM-GC | NO | Male | 68 | Multicentre |
| A92 | B-G1 F-G1 | B-E2 F-E2 | Antrum | CIM-NoGC | YES | Male | 60 | Soria |
| A93 | B-G1 F-G1 | B-E1 F-E1 | Antrum | CIM-NoGC | YES | Female | 41 | Soria |
| A94 | B-G1 F-G1 | B-E1 F-E1 | Antrum | CIM-NoGC | NA | Female | 51 | Soria |
| A95 | B-G0 F-G0 | B-E0 F-E0 | Incisura | Sanos | NO | Male | 34 | Soria |
| A96 | B-G1 F-G1 | B-E2 F-E2 | Incisura | CIM-GC | NO | Female | 51 | Multicentre |
| A97 | B-G1 F-G1 | I -E1 F-E1 | Antrum | CIM-GC | YES | Male | 72 | Soria |
| A98 | B-G1 F-G1 | B-E2 F-E2 | Antrum | CIM-GC | YES | Male | 81 | Multicentre |
| A99 | B-G1 F-G1 | B-E4 F-E4 | Incisura | CIM-NoGC | YES | Male | 67 | Marañón |

^a^ Histological subtypes of IM in the hematoxylin-eosin stained FFPE slides obtained before (B) and final (F) to the FFPE cuts used for RNA extraction. ^b^ Extension of IM in the sample: 25%>E1>0%, 50%>E2>25%, 75%>E3>50%, 100%≥E4>75%. ^c^ CIM-GC, IIM-GC: Sample of CIM or IIM at recruitment progressing to GC at the end of follow up. CIM/IIM-Not GC are CIM or IIM samples that do not progress to GC from recruitment to the end of follow-up. ^d^ Hospital or project from which the samples were obtained.
