# Supplementary material for: Gene expression study and pathway analysis of histological subtypes of intestinal metaplasia that progress to gastric cancer
Source: PLoS One. 2017 Apr 25;12(4):e0176043. doi: 10.1371/journal.pone.0176043 (PMC5404762; doi:10.1371/journal.pone.0176043)
Supplement: S4 Table — (DOC) [file pone.0176043.s006.doc]

**S4 Table.** Differentially expressed genes in the IIM-GC when compared to IIM-NoGC

| **Gene Symbol** | **Gene name** | **Fold Change ^a^** | **Nominal p-value** | **Asociated with IM or GC ^b^** | **Sign in IIM-GC *vs* Healthy ^c^** | **Sign in IIM-NoGC *vs* Healthy ^d^** | **Candidates of major interest ^e^** |
| --- | --- | --- | --- | --- | --- | --- | --- |
| *HLA-DRB1* | major histocompatibility complex, class II, DR beta 1 | 0.260 | 0.014 | YES [1] | YES | NO | YES |
| *RNU2-2* | RNA, U2 small nuclear 2 | 0.297 | 0.008 | New molecule | YES | NO | YES |
| *MME* | membrane metallo-endopeptidase | 0.363 | 0.007 | New molecule | NO | YES | NO |
| *MGAM* | maltase-glucoamylase (alpha-glucosidase) | 0.374 | 0.0005 | YES [2] | NO | YES | NO |
| *IK* | IK cytokine, down-regulator of HLA II | 0.384 | 0.002 | YES [3] | YES | NO | YES |
| *TMEM25* | transmembrane protein 25 | 0.386 | 0.005 | New molecule | NO | YES | NO |
| *GSTA1* | glutathione S-transferase alpha 1 | 0.386 | 0.029 | YES [4] | NO | YES | NO |
| *PPIA* | Peptidylprolyl isomerase A (cyclophilin A) | 0.393 | 0.027 | YES [5] | YES | NO | YES |
| *KARS* | lysyl-tRNA synthetase | 0.396 | 0.032 | New molecule | NO | NO | YES |
| *RHOA* | ras homolog family member A | 0.398 | 0.0002 | YES [6] | NO | YES | NO |
| *CDKL3* | cyclin-dependent kinase-like 3 | 0.403 | 0.0001 | New molecule | YES | NO | YES |
| *TMEM236* | transmembrane protein 236 | 0.409 | 0.001 | New molecule | NO | YES | NO |
| *RBP2* | retinol binding protein 2, cellular | 0.411 | 0.021 | YES [7] | YES | YES | NO |
| *CP* | ceruloplasmin (ferroxidase) | 0.412 | 0.040 | YES [8] | YES | NO | YES |
| *GIP* | gastric inhibitory polypeptide | 0.433 | 0.017 | New molecule | YES | YES | NO |
| *XPNPEP2* | X-prolyl aminopeptidase (aminopeptidase P) 2, membrane-bound | 0.442 | 0.020 | New molecule | YES | YES | NO |
| *GNAS* | GNAS complex locus | 0.445 | 0.009 | YES [9] | NO | NO | YES |
| *SART3* | squamous cell carcinoma antigen recognized by T cells 3 | 0.448 | 0.0003 | YES [10] | NO | NO | YES |
| *RBBP7* | retinoblastoma binding protein 7 | 0,45 | 0.001 | New molecule | YES | NO | YES |
| *AQP10* | aquaporin 10 | 0.465 | 0.014 | New molecule | NO | YES | NO |
| *SLC13A2* | solute carrier family 13 (sodium-dependent dicarboxylate transporter), member 2 | 0.470 | 0.007 | New molecule | YES | YES | NO |
| *DNAJB7* | DnaJ (Hsp40) homolog, subfamily B, member 7 | 0.482 | 0.001 | New molecule | NO | YES | NO |
| *XDH* | xanthine dehydrogenase | 0.486 | 0.006 | New molecule | YES | YES | NO |
| *DTNB* | dystrobrevin, beta | 0.487 | 0.001 | New molecule | NO | NO | YES |
| *LCT* | lactase | 0.493 | 0.002 | New molecule | NO | NO | YES |
| *ATP1B3* | ATPase, Na+/K+ transporting, beta 3 polypeptide | 0.499 | 0.002 | New molecule | YES | YES | NO |
| *FAM133B* | family with sequence similarity 133, member B | 2.001 | 0.004 | New molecule | NO | NO | YES |
| *FAM133CP* | family with sequence similarity 133, member CP | 2.001 | 0.004 | New molecule | NO | NO | YES |
| **Gene Symbol** | **Gene name** | **Fold Change ^a^** | **Nominal p-value** | **Asociated with IM or GC ^b^** | **Sign in IIM-GC *vs* Healthy ^c^** | **Sign en IIM-NoGC *vs* Healthy ^d^** | **Candidates of major interest ^e^** |
| *FAM133DP* | family with sequence similarity 133, member DP | 2.001 | 0.004 | New molecule | NO | NO | YES |
| *MORF4L1* | mortality factor 4 like 1 | 2.006 | 0.016 | New molecule | NO | NO | YES |
| *CXCL14* | chemokine [C-X-C motif) ligand 14 | 2.007 | 0.028 | YES [11] | NO | NO | YES |
| *LAPTM5* | lysosomal protein transmembrane 5 | 2.011 | 0.010 | New molecule | NO | NO | YES |
| *NUCB2* | nucleobindin 2 | 2.013 | 0.003 | YES [12] | NO | YES | NO |
| *RGS16* | regulator of G-protein signaling 16 | 2.022 | 0.000004 | New molecule | NO | NO | YES |
| *HIST2H3A* | histone cluster 2, H3a | 2.024 | 0.048 | New molecule | NO | NO | YES |
| *HIST2H3C* | histone cluster 2, H3c | 2.024 | 0.048 | New molecule | NO | NO | YES |
| *HIST2H3D* | histone cluster 2, H3d | 2.024 | 0.048 | New molecule | NO | NO | YES |
| *MYOF* | myoferlin | 2.035 | 0.006 | New molecule | YES | NO | YES |
| *C1R* | complement component 1, r subcomponent | 2.048 | 0.005 | New molecule | NO | NO | YES |
| *CCT6A* | chaperonin containing TCP1, subunit 6A [zeta 1) | 2.053 | 0.002 | New molecule | NO | NO | YES |
| *ABCC5* | ATP-binding cassette, sub-family C [CFTR/MRP), member 5 | 2.056 | 0.013 | YES [13] | NO | YES | NO |
| *HLA-A* | major histocompatibility complex, class I, A | 2.059 | 0.029 | YES [14] | YES | NO | YES |
| *RPS6KA2* | ribosomal protein S6 kinase, 90kDa, polypeptide 2 | 2.059 | 0.001 | New molecule | NO | NO | YES |
| *EIF5B* | eukaryotic translation initiation factor 5B | 2.059 | 0.004 | New molecule | NO | NO | YES |
| *C1QBP* | complement component 1, q subcomponent binding protein | 2.076 | 0.002 | New molecule | YES | NO | YES |
| *CTSB* | cathepsin B | 2.078 | 0.001 | YES [15] | NO | NO | YES |
| *IGKC* | immunoglobulin kappa constant | 2.089 | 0.014 | YES [16] | YES | YES | NO |
| *UHMK1* | U2AF homology motif [UHM) kinase 1 | 2.092 | 0.012 | New molecule | NO | NO | YES |
| *NOL7* | nucleolar protein 7, 27kDa | 2.095 | 0.001 | New molecule | NO | NO | YES |
| *MEGF6* | multiple EGF-like-domains 6 | 2.104 | 0.0001 | New molecule | NO | YES | NO |
| *SMNDC1* | survival motor neuron domain containing 1 | 2.104 | 0.013 | New molecule | YES | NO | YES |
| *ZNF259* | zinc finger protein 259 | 2.110 | 0.022 | New molecule | YES | NO | YES |
| *CANX* | calnexin | 2.117 | 0.027 | New molecule | YES | NO | YES |
| *TOMM22* | translocase of outer mitochondrial membrane 22 homolog [yeast) | 2.120 | 0.012 | New molecule | YES | NO | YES |
| *CAV1* | caveolin 1, caveolae protein, 22kDa | 2.144 | 0.0005 | YES [17] | NO | NO | YES |
| *CCDC144A /CCDC144B/ CCDC144C* | coiled-coil domain containing 144A-C | 2.148 | 0.014 | New molecule | NO | NO | YES |
| *ANP32B* | acidic [leucine-rich) nuclear phosphoprotein 32 family, member B | 2.155 | 0.006 | New molecule | NO | NO | YES |
| *RHOA* | ras homolog family member A | 2.16 | 0.047 | YES [6] | NO | YES | NO |
| *AFAP1-AS1* | AFAP1 antisense RNA 1 [non-protein coding) | 2.161 | 0.001 | New molecule | YES | YES | NO |
| **Gene Symbol** | **Gene name** | **Fold Change ^a^** | **Nominal p-value** | **Asociated with IM or GC ^b^** | **Sign in IIM-GC *vs* Healthy ^c^** | **Sign en IIM-NoGC *vs* Healthy ^d^** | **Candidates of major interest ^e^** |
| *HLA-DQB1* | major histocompatibility complex, class II, DQ beta 1 | 2.17 | 0.001 | YES [18] | YES | NO | YES |
| *PI3* | peptidase inhibitor 3, skin-derived | 2.183 | 0.012 | New molecule | YES | YES | NO |
| *CD53* | CD53 molecule | 2.17 | 0.0001 | New molecule | YES | NO | YES |
| *NHP2* | NHP2 ribonucleoprotein homolog [yeast) | 2.187 | 0.002 | New molecule | NO | NO | YES |
| *C3* | complement component 3 | 2.193 | 0.020 | New molecule relacionada [Clin Chim Acta.](http://www.ncbi.nlm.nih.gov/pubmed/17067565) 2007 Feb;377[1-2]:119-26. | NO | NO | YES |
| *CTBP2* | C-terminal binding protein 2 | 2.204 | 0.007 | New molecule | NO | NO | YES |
| *CRISPLD2* | cysteine-rich secretory protein LCCL domain containing 2 | 2.208 | 0.003 | New molecule | NO | NO | YES |
| *EIF3D* | eukaryotic translation initiation factor 3, subunit D | 2.211 | 0.003 | New molecule | NO | NO | YES |
| *GNL3* | guanine nucleotide binding protein-like 3 [nucleolar) / small nucleolar RNA, C/D box 19B | 2.218 | 0.0003 | New molecule | NO | NO | YES |
| *SNORD19B* | small nucleolar RNA, C/D box 19B | 2.218 | 0.0003 | New molecule | NO | NO | YES |
| *CD24* | CD24 molecule | 2.249 | 0.008 | YES [19] | YES | NO | YES |
| *HLA-DRB4* | major histocompatibility complex, class II, DR beta 4 | 2.25 | 0.024 | YES [1] | NO | NO | YES |
| *ZNF146* | zinc finger protein 146 | 2.264 | 0.0002 | New molecule | YES | NO | YES |
| *PDCD5* | programmed cell death 5 | 2.266 | 0.001 | YES [20] | NO | NO | YES |
| *VSIG1* | V-set and immunoglobulin domain containing 1 | 2.28 | 0.012 | YES [16] | NO | YES | NO |
| *SLC1A4* | solute carrier family 1 [glutamate/neutral amino acid transporter), member 4 | 2.307 | 0.001 | New molecule | YES | NO | YES |
| *IL1R2* | interleukin 1 receptor, type II | 2.31 | 0.019 | YES [21] | NO | NO | YES |
| *LYZ* | lysozyme | 2.32 | 0.042 | YES [22] | NO | YES | NO |
| *FABP5* | fatty acid binding protein 5 [psoriaYESs-associated) | 2.369 | 0.003 | YES [23] | NO | YES | NO |
| *FABP5P3* | fatty acid binding protein 5 pseudogene 3 | 2.369 | 0.003 | YES [23] | NO | YES | NO |
| *RAN* | RAN, member RAS oncogene family | 2.41 | 0.004 | YES [24] | NO | NO | YES |
| *HSP90AA1* | heat shock protein 90kDa alpha [cytosolic), class A member 1 | 2.412 | 0.0001 | YES [25] | NO | NO | YES |
| *CLU* | clusterin | 2.469 | 0.016 | YES [26] | YES | YES | NO |
| *ATP5A1* | ATP synthase, H+ transporting, mitochondrial F1 complex, alpha subunit 1, cardiac muscle | 1.304 | 0.016 | New molecule | YES | NO | YES |
| *HSP90AB1* | heat shock protein 90kDa alpha [cytosolic), class B member 1 | 2.479 | 0.003 | YES [25] | NO | NO | YES |
| *ATP6V0E1* | ATPase, H+ transporting, lysosomal 9kDa, V0 subunit e1 | 2.488 | 0.005 | New molecule | NO | NO | YES |
| *GIF* | gastric intrinsic factor [vitamin B synthesis) | 2.519 | 0.049 | YES [27] | NO | YES | NO |
| *KARS* | lysyl-tRNA synthetase | 2.542 | 0.019 | New molecule | NO | NO | YES |
| *IGHG1* | immunoglobulin heavy constant gamma 1 in heavy constant gamma | 2.670 | 0.011 | YES [16] | YES | YES | NO |
| **Gene Symbol** | **Gene name** | **Fold Change^a^** | **Nominal p-value** | **Asociated with IM or GC^b^** | **Sign in IIM-GC *vs* Healthy^c^** | **Sign in IIM-NoGC *vs* Healthy^d^** | **Candidates of major interest^e^** |
| *IGHG2* | immunoglobulin heavy constant gamma | 2.670 | 0.011 | YES [16] | YES | YES | NO |
| *IGHG3* | immunoglobulin heavy constant gamma | 2.670 | 0.011 | YES [16] | YES | YES | NO |
| *IGHG4* | immunoglobulin heavy constant gamma | 2.670 | 0.011 | YES [16] | YES | YES | NO |
| *IGHM* | immunoglobulin heavy constant gamma | 2.670 | 0.011 | YES [16] | YES | YES | NO |
| *IGHV4-31* | immunoglobulin heavy constant gamma | 2.670 | 0.011 | YES [16] | YES | YES | NO |
| *BASP1* | brain abundant, membrane attached signal protein 1 | 2.704 | 0.005 | New molecule | NO | YES | NO |
| *BPIFB1* | BPI fold containing family B, member 1 | 2.866 | 0.009 | New molecule | NO | NO | YES |
| *DPCR1* | diffuse panbronchiolitis critical region 1 | 1.519 | 0.024 | YES [Gastroenterology.](http://www.ncbi.nlm.nih.gov/pubmed/?term=DPCR1+gastric+cancer) 2010 Jul;139[1]:213-25.e3. | NO | YES | NO |
| *HLA-DQA1* | major histocompatibility complex, class II, DQ alpha 1 | 3.182 | 0.024 | YES [1] | YES | NO | YES |
| *HLA-DRB1* | major histocompatibility complex, class II, DR beta complex | 3.368 | 0.048 | YES [1] | YES | NO | YES |
| *HLA-DRB3* | major histocompatibility complex, class II, DR beta complex | 3.368 | 0.048 | YES [1] | YES | NO | YES |
| *HLA-DRB5* | major histocompatibility complex, class II, DR beta complex | 3.368 | 0.048 | YES [1] | YES | NO | YES |
| *LTF* | lactotransferrin | 3.432 | 0.019 | YES [28] | NO | YES | NO |
| *GKN2* | gastrokine 2 | 3.499 | 0.025 | YES [29] | YES | YES | NO |
| *CXCL17* | chemokine [C-X-C motif) ligand 17 | 3.506 | 0.002 | New molecule | NO | YES | NO |
| *GKN1* | gastrokine 1 | 3.834 | 0.038 | YES [30] | YES | YES | NO |
| *HLA-C* | major histocompatibility complex, class I, C | 4.417 | 0.011 | YES [14] | NO | NO | YES |
| *PGC* | progastricsin [pepsinogen C) | 5.004 | 0.002 | YES [31] | YES | YES | NO |

a, Fold change is the average expression of IIM-GC/IIM-NoGC. Genes are increasingly ordered by this variable. b, Genes already associated with IM or GC by expression, genetic association, proteomic or functional studies. "New molecule" means that this is the first time that this gene is identified as differentially expressed in IM. "Similar molecule" means that another member of the gene family was previously associated with IM. c,d, Significant differentially expressed genes in these comparisons. e, YES means that they are potential causal genes of progression.

**References**

1. Magnusson PKE, Enroth H, Eriksson I, et al. Gastric cancer and human leukocyte antigen: distinct DQ and DR alleles are associated with development of gastric cancer and infection by Helicobacter pylori. *Cancer Res.* 2001;61(6):2684-9.

2. Yang S. Gene amplifications at chromosome 7 of the human gastric cancer genome. *Int.J.Mol.Med.* 2007;20:225-231.

3. Cao LX, Le Bousse-Kerdiles MC, Clay D, Oshevski S, Jasmin C, Krief P. Implication of a new molecule IK in CD34+ hematopoietic progenitor cell proliferation and differentiation. *Blood* 1997;89(10):3615-3623.

4. Nguyen T V, Janssen MJR, van Oijen MGH, et al. Genetic polymorphisms in GSTA1, GSTP1, GSTT1, and GSTM1 and gastric cancer risk in a Vietnamese population. *Oncol. Res.* 2010;18(7):349-55.

5. Bai Z, Ye Y, Liang B, et al. Proteomics-based identification of a group of apoptosis-related proteins and biomarkers in gastric cancer. *Int. J. Oncol.* 2011;38(2):375-83.

6. Liu J, Zhang Y, Xu R, et al. PI3K/Akt-dependent phosphorylation of GSK3β and activation of RhoA regulate Wnt5a-induced gastric cancer cell migration. *Cell. Signal.* 2013;25(2):447-56.

7. Li L, Wang L, Song P, et al. Critical role of histone demethylase RBP2 in human gastric cancer angiogenesis. *Mol. Cancer* 2014;13:81.

8. Aquino PF, Fischer JSG, Neves-Ferreira AGC, et al. Are gastric cancer resection margin proteomic profiles more similar to those from controls or tumors? *J. Proteome Res.* 2012;11(12):5836-42.

9. Ikuta K, Seno H, Chiba T. Molecular changes leading to gastric cancer: a suggestion from rare-type gastric tumors with GNAS mutations. *Gastroenterology* 2014;146(5):1417-8.

10. Niiya F, Nishizaka S, Matsunaga K, et al. Expression of SART3 tumor-rejection antigen in gastric cancers. *Japanese J. cancer Res.* 2000;91(3):337-42.

11. Xue X. Abnormal hypermethylation of promoter region downregulates chemokine CXC ligand 14 expression in gastric cancer. *Int. J. Oncol.* 2013;43(5):1487-94.

12. Kalnina Z, Silina K, Bruvere R, et al. Molecular characterisation and expression analysis of SEREX-defined antigen NUCB2 in gastric epithelium, gastritis and gastric cancer. *Eur. J. Histochem.* 2009;53(1):2.

13. Wu Q, Yang Z, Xia L, et al. Methylation of miR-129-5p CpG island modulates multi-drug resistance in gastric cancer by targeting ABC transporters. *Oncotarget* 2014;5(22):11552-63.

14. Ueda Y, Ishikawa K, Shiraishi N, Yokoyama S, Kitano S. Clinical significance of HLA class I heavy chain expression in patients with gastric cancer. *J. Surg. Oncol.* 2008;97(5):451-455.

15. Qian Z, Zhu G, Tang L, et al. Whole genome gene copy number profiling of gastric cancer identifies PAK1 and KRAS gene amplification as therapy targets. *Genes. Chromosomes Cancer* 2014;53(11):883-94.

16. Li S, Lu A-P, Zhang L, Li Y-D. Anti-Helicobacter pylori immunoglobulin G (IgG) and IgA antibody responses and the value of clinical presentations in diagnosis of H. pylori infection in patients with precancerous lesions. *World J. Gastroenterol.* 2003;9(4):755-758.

17. Nam KH, Lee BL, Park JH, et al. Caveolin 1 expression correlates with poor prognosis and focal adhesion kinase expression in gastric cancer. *Pathobiology* 2013;80(2):87-94.

18. Watanabe Y, Aoyama N, Sakai T, et al. HLA-DQB1 locus and gastric cancer in Helicobacter pylori infection. *J. Gastroenterol. Hepatol.* 2006;21(2):420-424.

19. Wang Y-C, Wang J-L, Kong X, et al. CD24 mediates gastric carcinogenesis and promotes gastric cancer progression via STAT3 activation. *Apoptosis* 2014;19(4):643-656.

20. Xu H-Y, Chen Z-W, Pan Y-M, Fan L, Guan J, Lu Y-Y. Transfection of PDCD5 Effect on the Biological Behavior of Tumor Cells and Sensitized Gastric Cancer Cells to Cisplatin-Induced Apoptosis. *Dig. Dis. Sci.* 2012;57(7):1847-1856.

21. Kamangar F, Cheng C, Abnet CC, Rabkin CS. Interleukin-1B polymorphisms and gastric cancer risk--a meta-analysis. *Cancer Epidemiol. Biomarkers Prev.* 2006;15(10):1920-1928.

22. Lee HJ, Nam KT, Park HS, et al. Gene Expression Profiling of Metaplastic Lineages Identifies CDH17 as a Prognostic Marker in Early Stage Gastric Cancer. *Gastroenterology* 2010;139(1):358-366.

23. Kim KR, Oh SY, Park UC, et al. Gene expression profiling using oligonucleotide microarray in atrophic gastritis and intestinal metaplasia. *Korean J. Gastroenterol.* 2007;49(4):209-24.

24. Xie Y, Wang Y, Zhao Y, Guo Z. Single-nucleotide polymorphisms of microRNA processing machinery genes are associated with risk for gastric cancer. *Onco. Targets. Ther.* 2015;8:567-571.

25. Wang J, Cui S, Zhang X, Wu Y, Tang H. High expression of heat shock protein 90 is associated with tumor aggressiveness and poor prognosis in patients with advanced gastric cancer. *PLoS One* 2013;8(4):e62876.

26. Humphries JM, Penno MAS, Weiland F, et al. Identification and validation of novel candidate protein biomarkers for the detection of human gastric cancer. *Biochim. Biophys. Acta - Proteins Proteomics* 2014;1844(5):1051-1058.

27. Wu W, Juan WC, Liang CRMY, Yeoh KG, So J, Chung MCM. S100A9, GIF and AAT as potential combinatorial biomarkers in gastric cancer diagnosis and prognosis. *PROTEOMICS - Clin. Appl.* 2012;6(3-4):152-162.

28. Terashima M, Maesawa C, Oyama K, et al. Gene expression profiles in human gastric cancer: expression of maspin correlates with lymph node metastasis. *Br. J. Cancer* 2005;92(6):1130-1136.

29. Dai J, Zhang N, Wang J, Chen M, Chen J. Gastrokine-2 is downregulated in gastric cancer and its restoration suppresses gastric tumorigenesis and cancer metastasis. *Tumor Biol.* 2014;35(5):4199-4207.

30. Nardone G, Martin G, Rocco A, et al. Molecular expression of Gastrokine 1 in normal mucosa and in Helicobacter pylori-related preneoplastic and neoplastic gastric lesions. *Cancer Biol. Ther.* 2008;7(12):1890-1895.

31. Ning PF, Liu HJ, Yuan Y. Dynamic expression of pepsinogen C in gastric cancer, precancerous lesions and Helicobacter pylori associated gastric diseases. *World J. Gastroenterol.* 2005;11(17):2545-2548.
