# Supplementary material for: Gene expression study and pathway analysis of histological subtypes of intestinal metaplasia that progress to gastric cancer
Source: PLoS One. 2017 Apr 25;12(4):e0176043. doi: 10.1371/journal.pone.0176043 (PMC5404762; doi:10.1371/journal.pone.0176043)
Supplement: S5 Table — (DOC) [file pone.0176043.s007.doc]

**S5 Table. Differentially expressed genes in CIM-GC when compared to CIM-NoGC**

| **Gene Symbol** | **Gene name** | **Fold Change ^a^** | **Nominal p-value** | **Asociated with IM or GC ^b^** | **Sign in CIM-GC *vs* Healthy ^c^** | **Sign en CIM-NoGC *vs* Healthy ^d^** | **Candidates of major interest ^e^** |
| --- | --- | --- | --- | --- | --- | --- | --- |
| *REG1B* | regenerating islet-derived 1 beta | 0.421 | 0.011 | YES [23] | NO | YES | NO |
| *GPR110* | G protein-coupled receptor 110 | 0.440 | 0.015 | New molecule | NO | YES | NO |
| *CEACAM20* | Carcinoembryonic antigen-related cell adhesion molecule 20 | 0.461 | 0.046 | Similar molecule [32] | YES | YES | NO |
| *ANAPC5* | anaphase promoting complex subunit 5 | 0.461 | 0.031 | New molecule | NO | NO | YES |
| *EPB41L3* | erythrocyte membrane protein band 4.1-like 3 | 0.474 | 0.014 | YES [33] | NO | YES | NO |
| *IL1R2* | interleukin 1 receptor, type II | 0.475 | 0.017 | YES [21] | YES | NO | YES |
| *CXCL17* | chemokine (C-X-C motif) ligand 17 | 0.484 | 0.038 | New molecule | YES | YES | NO |
| *IGHG1* | immunoglobulin heavy constant gamma 1 | 0.493 | 0.014 | YES [16] | YES | NO | YES |
| *IGHM* | immunoglobulin heavy constant mu | 0.493 | 0.014 | YES [16] | YES | NO | YES |
| *IGHV4-31* | immunoglobulin heavy variable 4-31 | 0.493 | 0.014 | YES [16] | YES | NO | YES |
| *PRSS1* | protease, serine, 1 (trypsin 1) | 0.495 | 0.033 | New molecule | NO | NO | YES |
| *HLA-DRB4* | major histocompatibility complex, class II, DR beta 4 | 2.049 | 0.022 | YES [1] | NO | NO | YES |
| *GP2* | glycoprotein 2 (zymogen granule membrane) | 2.063 | 0.018 | New molecule | YES | NO | YES |
| *HOXA13* | homeobox A13 | 2.076 | 0.028 | YES [34] | YES | NO | YES |
| *IGFBP5* | insulin-like growth factor binding protein 5 | 2.127 | 0.004 | New molecule | YES | NO | YES |
| *OLFM4* | olfactomedin 4 | 3.332 | 0.0002 | YES [35] | YES | NO | YES |
| *HLA-DRB1* | major histocompatibility complex, class II, DR beta 1 | 3.475 | 0.021 | YES [1] | YES | NO | YES |
| *HLA-DRB3* | major histocompatibility complex, class II, DR beta 3 | 3.475 | 0.021 | YES [1] | YES | NO | YES |
| *HLA-DRB5* | major histocompatibility complex, class II, DR beta 5 | 3.475 | 0.021 | YES [1] | YES | NO | YES |

a, Fold change is the average expression of IIM-GC/IIM-NoGC. Genes are increasingly ordered by this variable. b, Genes already associated with IM or GC by expression, genetic association, proteomic or functional studies. "New molecule" means that this is the first time that this gene is identified as differentially expressed in IM. "Similar molecule" means that another member of the gene family was previously associated with IM. c,d, Significant differentially expressed genes in these comparisons. e, YES means that they are potential causal genes of progression.

**References**

1. Magnusson PKE, Enroth H, Eriksson I, et al. Gastric cancer and human leukocyte antigen: distinct DQ and DR alleles are associated with development of gastric cancer and infection by Helicobacter pylori. *Cancer Res.* 2001;61(6):2684-9.

16. Li S, Lu A-P, Zhang L, Li Y-D. Anti-Helicobacter pylori immunoglobulin G (IgG) and IgA antibody responses and the value of clinical presentations in diagnosis of H. pylori infection in patients with precancerous lesions. *World J. Gastroenterol.* 2003;9(4):755-758.

21. Kamangar F, Cheng C, Abnet CC, Rabkin CS. Interleukin-1B polymorphisms and gastric cancer risk--a meta-analysis. *Cancer Epidemiol. Biomarkers Prev.* 2006;15(10):1920-1928.

23. Kim KR, Oh SY, Park UC, et al. Gene expression profiling using oligonucleotide microarray in atrophic gastritis and intestinal metaplasia. *Korean J. Gastroenterol.* 2007;49(4):209-24.

32. Shi J, Xu S, He P, Xi Z. Expression of carcinoembryonic antigen-related cell adhesion molecule 1(CEACAM1) and its correlation with angiogenesis in gastric cancer. *Pathol. - Res. Pract.* 2014;210(8):473-476.

33. Li X, Zhang Y, Zhang H, et al. miRNA-223 Promotes Gastric Cancer Invasion and Metastasis by Targeting Tumor Suppressor EPB41L3. *Mol. Cancer Res.* 2011;9(7):824-833.

34. Han Y, Tu W-W, Wen Y-G, et al. Identification and validation that up-expression of HOXA13 is a novel independent prognostic marker of a worse outcome in gastric cancer based on immunohistochemistry. *Med. Oncol.* 2013;30(2):564.

35. Jang BG, Lee BL, Kim WH. Olfactomedin-related proteins 4 (OLFM4) expression is involved in early gastric carcinogenesis and of prognostic significance in advanced gastric cancer. *Virchows Arch.* 2015;467(3):285-294.
