# Supplementary material for: Gene expression study and pathway analysis of histological subtypes of intestinal metaplasia that progress to gastric cancer
Source: PLoS One. 2017 Apr 25;12(4):e0176043. doi: 10.1371/journal.pone.0176043 (PMC5404762; doi:10.1371/journal.pone.0176043)
Supplement: S6 Table — (DOC) [file pone.0176043.s008.doc]

**S6 Table.** Differentially expressed genes in IIM-GC when compared to CIM-GC

| **Symbol** | **Gene name** | **Fold change ^a^** | **Nominal p-value** |
| --- | --- | --- | --- |
| *EBF1* | early B-cell factor 1 | 0,357 | 1,435E-06 |
| *RNU2-2* | RNA, U2 small nuclear 2 | 0,382 | 2,951E-02 |
| *KCNE2* | potassium voltage-gated channel, Isk-related family, member 2 | 2,037 | 2,784E-02 |
| *ATP5A1* | ATP synthase, H+ transporting, mitochondrial F1 complex, alpha subunit 1, cardiac muscle | 2,042 | 4,789E-02 |
| *ANXA13* | annexin A13 | 2,067 | 1,271E-02 |
| *SLC1A4* | solute carrier family 1 (glutamate/neutral amino acid transporter), member 4 | 2,133 | 1,736E-03 |
| *SMNDC1* | survival motor neuron domain containing 1 | 2,157 | 8,342E-03 |
| *IGHG1* | immunoglobulin heavy constant gamma 1 (G1m marker) | 2,438 | 1,715E-02 |
| *IGHG2* | immunoglobulin heavy constant gamma 1 (G2m marker) | 2,438 | 1,715E-02 |
| *IGHG3* | immunoglobulin heavy constant gamma 1 (G3m marker) | 2,438 | 1,715E-02 |
| *IGHG4* | immunoglobulin heavy constant gamma 1 (G4m marker) | 2,438 | 1,715E-02 |
| *IGHM* | immunoglobulin heavy constant mu | 2,438 | 1,715E-02 |
| *IGHV4-31* | immunoglobulin heavy variable 4-31 | 2,438 | 1,715E-02 |
| *IL1R2* | interleukin 1 receptor, type II | 2,563 | 7,000E-03 |
| *GIF* | gastric intrinsic factor (vitamin B synthesis) | 2,611 | 3,531E-02 |
| *CXCL17* | chemokine (C-X-C motif) ligand 17 | 2,758 | 9,867E-03 |
| *ZNF146* | zinc finger protein 146 | 2,779 | 3,256E-03 |
| *ATP4B* | ATPase, H+/K+ exchanging, beta polypeptide | 3,225 | 2,212E-02 |
| *PGC* | progastricsin (pepsinogen C) | 3,364 | 1,535E-02 |

^a^, Fold change is the average expression of IIM-GC/CIM-GC. Genes are increasingly ordered by this variable.
