# Supplementary material for: Gene expression study and pathway analysis of histological subtypes of intestinal metaplasia that progress to gastric cancer
Source: PLoS One. 2017 Apr 25;12(4):e0176043. doi: 10.1371/journal.pone.0176043 (PMC5404762; doi:10.1371/journal.pone.0176043)
Supplement: S7 Table — (DOC) [file pone.0176043.s009.doc]

**S7 Table.** Differentially expressed genes in IIM-NoGC when compared to CIM-NoGC

| **Differentially expressed genes for IIM-NoGC vs CIM-NoGC** | | | |
| --- | --- | --- | --- |
| **Symbol** | **Gene name** | **Fold change ^a^** | **Nominal p-value** |
| *GKN2* | gastrokine 2 | 0,302 | 1,845E-02 |
| *GKN1* | gastrokine 1 | 0,305 | 4,275E-02 |
| *BPIFB1* | BPI fold containing family B, member 1 | 0,355 | 5,060E-03 |
| *PGC* | progastricsin (pepsinogen C) | 0,378 | 3,607E-02 |
| *CXCL17* | chemokine (C-X-C motif) ligand 17 | 0,381 | 8,545E-03 |
| *IGHG1* | immunoglobulin heavy constant gamma 1 (G1m marker) | 0,401 | 9,177E-03 |
| *IGHG2* | immunoglobulin heavy constant gamma 1 (G2m marker) | 0,401 | 9,177E-03 |
| *IGHG3* | immunoglobulin heavy constant gamma 1 (G3m marker) | 0,401 | 9,177E-03 |
| *IGHG4* | immunoglobulin heavy constant gamma 1 (G4m marker) 2 (G2m marker) /// immunoglobulin heavy constant gamma 3 (G3m marker) /// immunoglobulin heavy constant gamma 4 (G4m marker) /// immunoglobulin heavy constant mu /// immunoglobulin heavy variable 4-31 | 0,401 | 9,177E-03 |
| *IGHM* | immunoglobulin heavy constant mu | 0,401 | 9,177E-03 |
| *IGHV4-31* | immunoglobulin heavy variable 4-31 | 0,401 | 9,177E-03 |
| *EBF1* | early B-cell factor 1 | 0,455 | 4,697E-05 |
| *TMPRSS15* | transmembrane protease, serine 15 | 2,072 | 3,838E-02 |
| *HOXA13* | homeobox A13 | 2,208 | 2,228E-02 |
| *NPY6R* | neuropeptide Y receptor Y6 (pseudogene) | 2,298 | 8,155E-04 |
| *TMEM25* | transmembrane protein 25 | 2,341 | 5,759E-03 |
| *SLC26A3* | solute carrier family 26, member 3 | 2,614 | 2,760E-02 |

^a^, Fold change is the average expression of IIM-NoGC/CIM-NoGC. Genes are increasingly ordered by this variable.
