# Supplementary material for: Gene expression study and pathway analysis of histological subtypes of intestinal metaplasia that progress to gastric cancer
Source: PLoS One. 2017 Apr 25;12(4):e0176043. doi: 10.1371/journal.pone.0176043 (PMC5404762; doi:10.1371/journal.pone.0176043)
Supplement: S8 Table — (DOC) [file pone.0176043.s010.doc]

**S8 Table.** Differentially expressed genes in the IM-NoGC when compared to healthy gastric mucosa

| **Gene symbol** | **Gene name** | **Fold Change** ^a^ | **Nominal p-value** | **Adjusted p-value ^b^** | **Previously associated with IM ^c^** |
| --- | --- | --- | --- | --- | --- |
| GKN1 | gastrokine 1 | 0.153 | 2.309E-04 | 1.129E-02 | YES [30] |
| PGC | progastricsin (pepsinogen C) | 0.169 | 2.012E-05 | 1.731E-03 | YES [36] |
| GAST | gastrin | 0.170 | 4.873E-06 | 5.537E-04 | YES [37] |
| SST | somatostatin | 0.183 | 1.815E-08 | 4.789E-06 | New molecule |
| GKN2 | gastrokine 2 | 0.208 | 4.150E-04 | 1.748E-02 | YES [29] |
| SLC5A5 | solute carrier family 5 (sodium iodide symporter), member 5 | 0.236 | 1.022E-04 | 6.144E-03 | YES [38] |
| GLUL | glutamate-ammonia ligase | 0.260 | 2.837E-09 | 9.728E-07 | New molecule |
| CLU | clusterin | 0.290 | 9.175E-06 | 9.217E-04 | YES [26] |
| SCGB2A1 | secretoglobin, family 2A, member 1 | 0.291 | 1.094E-07 | 2.319E-05 | New molecule |
| RGNEF | 190 kDa guanine nucleotide exchange factor | 0.304 | 3.121E-08 | 7.771E-06 | New molecule |
| FUT9 | fucosyltransferase 9 (alpha (1,3) fucosyltransferase) | 0.312 | 6.909E-07 | 1.090E-04 | Similar molecule [39] |
| ERO1LB | ERO1-like beta (S. cerevisiae) | 0.315 | 4.940E-09 | 1.554E-06 | New molecule |
| PSAPL1 | prosaposin-like 1 (gene/pseudogene) | 0.315 | 2.373E-05 | 1.952E-03 | New molecule |
| CXCL17 | chemokine (C-X-C motif) ligand 17 | 0.315 | 4.644E-04 | 1.898E-02 | YES [11] |
| MAL | mal, T-cell differentiation protein | 0.318 | 1.658E-04 | 8.809E-03 | New molecule |
| GIF | gastric intrinsic factor (vitamin B synthesis) | 0.324 | 1.644E-03 | 4.746E-02 | YES [27] |
| TFF2 | trefoil factor 2 | 0.328 | 1.716E-04 | 9.042E-03 | Yes [40] |
| ZNF662 | zinc finger protein 662 | 0.348 | 1.963E-07 | 3.836E-05 | New molecule |
| NKX6-2 | NK6 homeobox 2 | 0.357 | 7.947E-05 | 5.017E-03 | New molecule |
| VSIG1 | V-set and immunoglobulin domain containing 1 | 0.360 | 8.284E-05 | 5.207E-03 | YES [16] |
| FMOD | fibromodulin | 0.366 | 6.857E-06 | 7.258E-04 | New molecule |
| PCSK2 | proprotein convertase subtilisin/kexin type 2 | 0.375 | 4.615E-09 | 1.468E-06 | New molecule |
| DEPTOR | DEP domain containing MTOR-interacting protein | 0.379 | 2.496E-08 | 6.381E-06 | New molecule |
| GPRC5B | G protein-coupled receptor, family C, group 5, member B | 0.384 | 1.051E-07 | 2.254E-05 | New molecule |
| CSTA | cystatin A (stefin A) | 0.384 | 2.668E-04 | 1.241E-02 | New molecule |
| NRG4 | neuregulin 4 | 0.387 | 1.428E-06 | 1.995E-04 | New molecule |
| SOSTDC1 | sclerostin domain containing 1 | 0.387 | 8.559E-05 | 5.337E-03 | New molecule |
| **Gene symbol** | **Gene name** | **Fold Change** ^a^ | **Nominal p-value** | **Adjusted p-value ^b^** | **Previously associated with IM ^c^** |
| ALDH3A1 | aldehyde dehydrogenase 3 family, member A1 | 0.397 | 1.190E-04 | 6.949E-03 | New molecule |
| KCNE2 | potassium voltage-gated channel, Isk-related family, member 2 | 0.405 | 4.611E-04 | 1.890E-02 | New molecule |
| PDE4C | phosphodiesterase 4C, cAMP-specific | 0.407 | 2.787E-05 | 2.210E-03 | New molecule |
| RNASE1 | ribonuclease, RNase A family, 1 (pancreatic) | 0.407 | 1.017E-04 | 6.128E-03 | New molecule |
| C6 | complement component 6 | 0.408 | 7.431E-06 | 7.723E-04 | New molecule |
| APLP1 | amyloid beta (A4) precursor-like protein 1 | 0.412 | 9.674E-06 | 9.601E-04 | New molecule |
| SPINK1 | serine peptidase inhibitor, Kazal type 1 | 0.415 | 2.524E-05 | 2.050E-03 | New molecule |
| TESC | tescalcin | 0.421 | 8.419E-05 | 5.280E-03 | New molecule |
| TCN1 | transcobalamin I (vitamin B12 binding protein, R binder family) | 0.423 | 1.784E-04 | 9.286E-03 | New molecule |
| PSD3 | pleckstrin and Sec7 domain containing 3 | 0.423 | 1.876E-05 | 1.642E-03 | New molecule |
| MUC5AC | mucin 5AC, oligomeric mucus/gel-forming | 0.425 | 8.937E-04 | 3.072E-02 | Yes [41] |
| DGKD | diacylglycerol kinase, delta 130kDa | 0.431 | 2.786E-06 | 3.491E-04 | New molecule |
| FABP5 | fatty acid binding protein 5 (psoriasis-ssociated) | 0.432 | 1.554E-04 | 8.404E-03 | YES [42] |
| FABP5P3 | fatty acid binding protein 5 pseudogen 3 | 0.432 | 1.554E-04 | 8.404E-03 | Similar molecule [42] |
| FOXA2 | forkhead box A2 | 0.439 | 9.992E-07 | 1.502E-04 | New molecule |
| CA9 | carbonic anhydrase IX | 0.439 | 1.547E-05 | 1.403E-03 | YES [43] |
| REP15 | RAB15 effector protein | 0.439 | 6.520E-06 | 7.018E-04 | New molecule |
| B4GALNT3 | beta-1,4-N-acetyl-galactosaminyl transferase 3 | 0.443 | 2.448E-07 | 4.573E-05 | New molecule |
| SNORD116-14 | small nucleolar RNA, C/D box 116-14 | 0.443 | 1.378E-04 | 7.675E-03 | New molecule |
| SNORD116-15 | small nucleolar RNA, C/D box 116-15 | 0,443 | 2,448E-07 | 4,573E-05 | New molecule |
| SNORD116-17 | small nucleolar RNA, C/D box 116-17 | 0,443 | 2,448E-07 | 4,573E-05 | New molecule |
| SNORD116-19 | small nucleolar RNA, C/D box 116-19 | 0,443 | 2,448E-07 | 4,573E-05 | New molecule |
| SNORD116-20 | small nucleolar RNA, C/D box 116-20 | 0,443 | 2,448E-07 | 4,573E-05 | New molecule |
| SNORD116-21 | small nucleolar RNA, C/D box 116-21 | 0,443 | 2,448E-07 | 4,573E-05 | New molecule |
| SNRPN | small nuclear ribonucleoprotein polypeptide N | 0,443 | 2,448E-07 | 4,573E-05 | New molecule |
| CCKAR | cholecystokinin A receptor | 0.443 | 5.296E-05 | 3.663E-03 | New molecule |
| SLC29A1 | solute carrier family 29 (nucleoside transporters), member 1 | 0.447 | 7.732E-07 | 1.205E-04 | YES [38] |
| LIFR | leukemia inhibitory factor receptor alpha | 0.450 | 6.630E-06 | 7.083E-04 | New molecule |
| SOX21-AS1 | SOX21 antisense RNA 1 (non-protein coding) | 0.451 | 1.506E-05 | 1.375E-03 | New molecule |
| CHGB | chromogranin B (secretogranin 1) | 0.453 | 4.041E-05 | 2.957E-03 | New molecule |
| **Gene symbol** | **Gene name** | **Fold Change ^a^** | **Nominal p-value** | **Adjusted p-value ^b^** | **Previously associated with IM ^c^** |
| LEPREL1 | leprecan-like 1 | 0.454 | 7.796E-06 | 8.000E-04 | New molecule |
| TSPAN5 | tetraspanin 5 | 0.454 | 7.230E-07 | 1.134E-04 | New molecule |
| VSIG2 | V-set and immunoglobulin domain containing 2 | 0.455 | 1.792E-06 | 2.431E-04 | New molecule |
| RAB27A | RAB27A, member RAS oncogene family | 0.455 | 4.036E-05 | 2.957E-03 | New molecule |
| CAPN9 | calpain 9 | 0.458 | 3.048E-04 | 1.378E-02 | New molecule |
| NUCB2 | nucleobindin 2 | 0.458 | 1.819E-05 | 1.600E-03 | New molecule |
| DPT | dermatopontin | 0.461 | 1.006E-07 | 2.183E-05 | New molecule |
| SNORD116-22 | small nucleolar RNA, C/D box 116-22 | 0.464 | 3.306E-04 | 1.470E-02 | New molecule |
| FBP2 | fructose-1,6-bisphosphatase 2 | 0.475 | 6.546E-07 | 1.038E-04 | New molecule |
| GPR64 | G protein-coupled receptor 64 | 0.475 | 6.517E-06 | 7.018E-04 | New molecule |
| PTPRN2 | protein tyrosine phosphatase, receptor type, N polypeptide 2 | 0.475 | 3.177E-07 | 5.604E-05 | New molecule |
| MFSD4 | major facilitator superfamily domain containing 4 | 0.475 | 6.555E-05 | 4.314E-03 | New molecule |
| SPTSSB | serine palmitoyltransferase, small subunit B | 0.476 | 2.052E-04 | 1.037E-02 | New molecule |
| CLDN18 | claudin 18 | 0.478 | 9.232E-04 | 3.138E-02 | Similar molecule [42] |
| FAM101A | family with sequence similarity 101, member A | 0.478 | 3.970E-06 | 4.640E-04 | New molecule |
| COL27A1 | collagen, type XXVII, alpha 1 | 0.478 | 1.775E-04 | 9.263E-03 | YES [44] |
| MICALL1 | MICAL-like 1 | 0.478 | 5.210E-06 | 5.838E-04 | New molecule |
| IGFBP2 | insulin-like growth factor binding protein 2, 36kDa | 0.480 | 1.507E-06 | 2.090E-04 | New molecule |
| RMST | rhabdomyosarcoma 2 associated transcript (non-protein coding) | 0.480 | 3.203E-06 | 3.928E-04 | New molecule |
| CYSTM1 | cysteine-rich transmembrane module containing 1 | 0.480 | 1.099E-03 | 3.544E-02 | New molecule |
| GSTM3 | glutathione S-transferase mu 3 (brain) | 0.480 | 8.848E-05 | 5.477E-03 | New molecule |
| IMPDH1 | IMP (inosine 5'-monophosphate) dehydrogenase 1 | 0.481 | 6.895E-06 | 7.265E-04 | New molecule |
| FGA | fibrinogen alpha chain | 0.486 | 3.076E-06 | 3.821E-04 | New molecule |
| EPB41L1 | erythrocyte membrane protein band 4.1-like 1 | 0.487 | 5.508E-05 | 3.768E-03 | New molecule |
| PPFIBP1 | PTPRF interacting protein, binding protein 1 (liprin beta 1) | 0.488 | 3.137E-06 | 3.888E-04 | New molecule |
| TIMP3 | TIMP metallopeptidase inhibitor 3 | 0.489 | 8.041E-05 | 5.065E-03 | New molecule |
| AGSK1 | golgin subfamily A member 2-like | 0.492 | 2.688E-05 | 2.148E-03 | New molecule |
| LOC727849 | golgin A2 pseudogene | 0.492 | 2.688E-05 | 2.148E-03 | New molecule |
| RGMB | RGM domain family, member B | 0.493 | 1.062E-05 | 1.036E-03 | New molecule |
| PER3 | period homolog 3 (Drosophila) | 0.494 | 1.382E-04 | 7.690E-03 | New molecule |
| **Gene symbol** | **Gene name** | **Fold Change ^a^** | **Nominal p-value** | **Adjusted p-value ^b^** | **Previously associated with IM ^c^** |
| CUEDC1 | CUE domain containing 1 | 0.495 | 1.890E-09 | 7.074E-07 | New molecule |
| PDIA2 | protein disulfide isomerase family A, member 2 | 0.496 | 1.449E-05 | 1.334E-03 | New molecule |
| TFCP2L1 | transcription factor CP2-like 1 | 0.497 | 2.189E-06 | 2.904E-04 | New molecule |
| SYNE2 | spectrin repeat containing, nuclear envelope 2 | 0.497 | 1.623E-05 | 1.453E-03 | New molecule |
| TAGLN2 | transgelin 2 | 0.498 | 4.528E-05 | 3.233E-03 | New molecule |
| SNORD116-8 | small nucleolar RNA, C/D box 116-8 | 0.500 | 5.424E-04 | 2.121E-02 | New molecule |
| MECOM | MDS1 and EVI1 complex locus | 0.500 | 1.378E-05 | 1.289E-03 | New molecule |
| SLC4A4 | solute carrier family 4, sodium bicarbonate cotransporter, member 4 | 2.003 | 2.670E-06 | 3.398E-04 | Similar molecule [38] |
| DQX1 | DEAQ box RNA-dependent ATPase 1 | 2.005 | 5.849E-06 | 6.429E-04 | New molecule |
| MTCH2 | mitochondrial carrier 2 | 2.015 | 1.274E-05 | 1.213E-03 | New molecule |
| KBTBD11 | kelch repeat and BTB (POZ) domain containing 11 | 2.017 | 1.696E-05 | 1.503E-03 | New molecule |
| SULT1B1 | sulfotransferase family, cytosolic, 1B, member 1 | 2.017 | 3.470E-05 | 2.629E-03 | YES [39] |
| KIF13A | kinesin family member 13A | 2.019 | 4.478E-05 | 3.225E-03 | New molecule |
| CCND2 | cyclin D2 | 2.021 | 1.770E-08 | 4.715E-06 | New molecule |
| SLC28A1 | solute carrier family 28 (sodium-coupled nucleoside transporter), member 1 | 2.021 | 1.022E-04 | 6.144E-03 | YES [38] |
| LEAP2 | liver expressed antimicrobial peptide 2 | 2.022 | 6.472E-06 | 6.993E-04 | New molecule |
| ACOX2 | acyl-CoA oxidase 2, branched chain | 2.024 | 1.145E-04 | 6.739E-03 | New molecule |
| CFI | complement factor I | 2.024 | 1.678E-04 | 8.871E-03 | New molecule |
| MIR612 | microRNA 612 | 2.024 | 2.795E-04 | 1.287E-02 | New molecule |
| NEAT1 | nuclear paraspeckle assembly transcript 1 (non-protein coding) | 2.024 | 2.795E-04 | 1.287E-02 | New molecule |
| SLC9A3R1 | solute carrier family 9, subfamily A (NHE3, cation proton antiporter 3), member 3 regulator 1 | 2.024 | 2.179E-05 | 1.836E-03 | Similar molecule [38] |
| BHLHE41 | basic helix-loop-helix family, member e41 | 2.024 | 1.370E-04 | 7.644E-03 | New molecule |
| NAGS | N-acetylglutamate synthase | 2.028 | 2.431E-05 | 1.981E-03 | New molecule |
| STARD5 | StAR-related lipid transfer (START) domain containing 5 | 2.029 | 1.275E-05 | 1.213E-03 | New molecule |
| SLC27A2 | solute carrier family 27 (fatty acid transporter), member 2 | 2.037 | 6.406E-06 | 6.934E-04 | Similar molecule [38] |
| CES3 | carboxylesterase 3 | 2.039 | 1.695E-08 | 4.562E-06 | New molecule |
| HOXB7 | homeobox B7 | 2.042 | 2.470E-06 | 3.186E-04 | New molecule |
| CHST5 | carbohydrate (N-acetylglucosamine 6-O) sulfotransferase 5 | 2.044 | 2.266E-07 | 4.311E-05 | New molecule |
| CDH3 | cadherin 3, type 1, P-cadherin (placental) | 2.045 | 2.037E-05 | 1.748E-03 | New molecule |
| IGLC1 | immunoglobulin lambda constant 1 (Mcg marker) immunoglobulin lambda variable 1-44 | 2.049 | 5.773E-04 | 2.228E-02 | New molecule |
| **Gene symbol** | **Gene name** | **Fold Change ^a^** | **Nominal p-value** | **Adjusted p-value ^b^** | **Previously associated with IM ^c^** |
| CYAT1 | immunoglobulin lambda light chain-like | 2.049 | 5.773E-04 | 2.228E-02 | New molecule |
| PLEKHS1 | pleckstrin homology domain containing, family S member 1 | 2.049 | 3.275E-04 | 1.461E-02 | New molecule |
| MGAM | maltase-glucoamylase (alpha-glucosidase) | 2.050 | 4.555E-04 | 1.875E-02 | New molecule |
| HADHA | hydroxyacyl-CoA dehydrogenase/3-ketoacyl-CoA thiolase/enoyl-CoA hydratase (trifunctional protein), alpha subunit | 2.054 | 1.002E-05 | 9.864E-04 | New molecule |
| CD79A | CD79a molecule, immunoglobulin-associated alpha | 2.056 | 1.057E-03 | 3.449E-02 | New molecule |
| SIPA1L2 | signal-induced proliferation-associated 1 like 2 | 2.057 | 9.467E-07 | 1.434E-04 | New molecule |
| INPP5J | inositol polyphosphate-5-phosphatase J | 2.063 | 2.740E-06 | 3.449E-04 | New molecule |
| RASSF4 | Ras association (RalGDS/AF-6) domain family member 4 | 2.066 | 1.574E-07 | 3.173E-05 | New molecule |
| LGALS4 | lectin, galactoside-binding, soluble, 4 | 2.067 | 9.585E-05 | 5.870E-03 | New molecule |
| CHN2 | chimerin (chimaerin) 2 | 2.069 | 9.991E-06 | 9.847E-04 | New molecule |
| AIM1L | absent in melanoma 1-like | 2.071 | 1.709E-09 | 6.569E-07 | New molecule |
| ERVK-7 | endogenous retrovirus group K, member 7 | 2.071 | 5.729E-05 | 3.878E-03 | New molecule |
| FGFBP1 | fibroblast growth factor binding protein 1 | 2.074 | 4.077E-05 | 2.974E-03 | New molecule |
| SLC15A2 | solute carrier family 15 (H+/peptide transporter), member 2 | 2.076 | 3.788E-06 | 4.454E-04 | Similar molecule [38] |
| TPK1 | thiamin pyrophosphokinase 1 | 2.081 | 6.030E-06 | 6.590E-04 | New molecule |
| GLTPD2 | glycolipid transfer protein domain containing 2 | 2.082 | 2.038E-07 | 3.928E-05 | New molecule |
| FAM3D | family with sequence similarity 3, member D | 2.084 | 3.432E-06 | 4.137E-04 | New molecule |
| HSD17B11 | hydroxysteroid (17-beta) dehydrogenase 11 | 2.095 | 7.806E-05 | 4.972E-03 | New molecule |
| EVI2B | ecotropic viral integration site 2B | 2.096 | 6.878E-05 | 4.501E-03 | New molecule |
| PON2 | paraoxonase 2 | 2.098 | 2.556E-06 | 3.275E-04 | New molecule |
| HOXB13 | homeobox B13 | 2.099 | 2.508E-04 | 1.187E-02 | New molecule |
| UGT2B17 | UDP glucuronosyltransferase 2 family, polypeptide B17 | 2.099 | 1.443E-03 | 4.324E-02 | Similar molecule [39] |
| SLC25A37 | solute carrier family 25 (mitochondrial iron transporter), member 37 | 2.100 | 2.730E-06 | 3.444E-04 | Similar molecule [38] |
| SLC25A15 | solute carrier family 25 (mitochondrial carrier; ornithine transporter) member 15 | 2.100 | 2.178E-05 | 1.836E-03 | Similar molecule [38] |
| DGAT1 | diacylglycerol O-acyltransferase 1 | 2.102 | 3.156E-07 | 5.595E-05 | New molecule |
| GPT | glutamic-pyruvate transaminase (alanine aminotransferase) | 2.104 | 2.211E-06 | 2.917E-04 | New molecule |
| CASP10 | caspase 10, apoptosis-related cysteine peptidase | 2.111 | 1.504E-05 | 1.375E-03 | New molecule |
| CYP4F3 | cytochrome P450, family 4, subfamily F, polypeptide 3 | 2.113 | 2.312E-05 | 1.913E-03 | New molecule |
| LPGAT1 | lysophosphatidylglycerol acyltransferase 1 | 2.120 | 1.047E-06 | 1.545E-04 | New molecule |
| **Gene symbol** | **Gene name** | **Fold Change ^a^** | **Nominal p-value** | **Adjusted p-value ^b^** | **Previously associated with IM ^c^** |
| IGHA1 | immunoglobulin heavy constant alpha 1 | 2.121 | 9.189E-05 | 5.658E-03 | New molecule |
| CASC5 | cancer susceptibility candidate 5 | 2.121 | 1.671E-04 | 8.850E-03 | New molecule |
| MAOB | monoamine oxidase B | 2.121 | 1.607E-04 | 8.591E-03 | New molecule |
| LOC100132330 | mal, T-cell differentiation protein-like pseudogene | 2.124 | 3.979E-07 | 6.842E-05 | New molecule |
| CRYL1 | crystallin, lambda 1 | 2.125 | 2.998E-04 | 1.360E-02 | New molecule |
| MACC1 | metastasis associated in colon cancer 1 | 2.127 | 7.032E-06 | 7.348E-04 | New molecule |
| NPC1L1 | NPC1 (Niemann-Pick disease, type C1, gene)-like 1 | 2.128 | 1.359E-06 | 1.926E-04 | New molecule |
| SLAMF7 | SLAM family member 7 | 2.129 | 4.395E-04 | 1.826E-02 | New molecule |
| NAT2 | N-acetyltransferase 2 (arylamine N-acetyltransferase) | 2.130 | 2.634E-06 | 3.359E-04 | New molecule |
| HSD11B2 | hydroxysteroid (11-beta) dehydrogenase 2 | 2.131 | 8.303E-06 | 8.442E-04 | New molecule |
| EPPK1 | epiplakin 1 | 2.132 | 7.851E-05 | 4.995E-03 | New molecule |
| AATK | apoptosis-associated tyrosine kinase | 2.134 | 4.615E-07 | 7.680E-05 | New molecule |
| PLA2G12B | phospholipase A2, group XIIB | 2.137 | 3.147E-05 | 2.448E-03 | New molecule |
| MYB | v-myb myeloblastosis viral oncogene homolog (avian) | 2.139 | 6.618E-06 | 7.083E-04 | New molecule |
| ATP7B | ATPase, Cu++ transporting, beta polypeptide | 2.139 | 1.192E-06 | 1.720E-04 | New molecule |
| TSPAN3 | Tetraspanin 3 | 2.140 | 5.509E-04 | 2.146E-02 | New molecule |
| ACBD4 | acyl-CoA binding domain containing 4 | 2.141 | 8.868E-06 | 8.972E-04 | New molecule |
| TP53INP2 | tumor protein p53 inducible nuclear protein 2 | 2.141 | 1.869E-05 | 1.639E-03 | New molecule |
| ARL4A | ADP-ribosylation factor-like 4A | 2.142 | 5.842E-07 | 9.371E-05 | New molecule |
| SLC52A1 | solute carrier family 52, riboflavin transporter, member 1 | 2.143 | 6.031E-05 | 4.025E-03 | Similar molecule [38] |
| PLIN3 | perilipin 3 | 2.146 | 8.033E-05 | 5.065E-03 | New molecule |
| FAM3C | family with sequence similarity 3, member C | 2.154 | 1.300E-04 | 7.420E-03 | New molecule |
| ITPK1 | inositol-tetrakisphosphate 1-kinase | 2.167 | 5.557E-06 | 6.179E-04 | New molecule |
| FABP2 | fatty acid binding protein 2, intestinal | 2.167 | 5.087E-06 | 5.711E-04 | YES [42] |
| DERL3 | derlin 3 | 2.170 | 8.755E-06 | 8.873E-04 | New molecule |
| SLX1A-SULT1A3 | SLX1A-SULT1A3 readthrough | 2.180 | 4.797E-06 | 5.482E-04 | New molecule |
| SLX1B-SULT1A4 | SLX1B-SULT1A4 readthrough | 2,180 | 4,797E-06 | 5,482E-04 | New molecule |
| SULT1A2 | sulfotransferase family, cytosolic, 1A, phenol-preferring, member 2 | 2,180 | 4,797E-06 | 5,482E-04 | YES [39] |
| SULT1A3 | sulfotransferase family, cytosolic, 1A, phenol-preferring, member 3 | 2,180 | 4,797E-06 | 5,482E-04 | YES [39] |
|  |  |  |  |  |  |
| **Gene symbol** | **Gene name** | **Fold Change ^a^** | **Nominal p-value** | **Adjusted p-value ^b^** | **Previously associated with IM ^c^** |
| SULT1A4 | sulfotransferase family, cytosolic, 1A, phenol-preferring, member 4 | 2,180 | 4,797E-06 | 5,482E-04 | YES [39] |
| SULT1A1 | sulfotransferase family, cytosolic, 1A, phenol-preferring, member 1 | 2.185 | 3.305E-06 | 4.036E-04 | YES [39] |
| CCL24 | chemokine (C-C motif) ligand 24 | 2.188 | 8.839E-07 | 1.357E-04 | New molecule |
| S100A16 | S100 calcium binding protein A16 | 2.190 | 1.678E-05 | 1.492E-03 | New molecule |
| MAZ | MYC-associated zinc finger protein (purine-binding transcription factor) | 2.192 | 6.915E-06 | 7.265E-04 | New molecule |
| LIPA | lipase A, lysosomal acid, cholesterol esterase | 2.195 | 5.418E-05 | 3.725E-03 | New molecule |
| HMOX1 | heme oxygenase (decycling) 1 | 2.195 | 9.573E-06 | 9.517E-04 | New molecule |
| CKAP2 | Cytoskeleton associated protein 2 | 2.196 | 4.018E-04 | 1.706E-02 | New molecule |
| RNU5F-1 | RNA, U5F small nuclear 1 | 2.204 | 2.501E-06 | 3.219E-04 | New molecule |
| UGT2B15 | UDP glucuronosyltransferase 2 family, polypeptide B15 nosyltransferase 2 family, polypeptide B17 | 2.209 | 1.646E-03 | 4.749E-02 | Similar molecule [39] |
| PI3 | peptidase inhibitor 3, skin-derived | 2.211 | 7.569E-04 | 2.741E-02 | New molecule |
| FCGBP | Fc fragment of IgG binding protein | 2.223 | 3.169E-05 | 2.461E-03 | New molecule |
| PLS1 | plastin 1 | 2.223 | 9.741E-05 | 5.939E-03 | New molecule |
| SLC25A34 | solute carrier family 25, member 34 | 2.227 | 1.330E-07 | 2.759E-05 | Similar molecule [38] |
| HEBP1 | heme binding protein 1 | 2.232 | 2.742E-04 | 1.271E-02 | New molecule |
| PLIN2 | perilipin 2 | 2.235 | 3.686E-05 | 2.755E-03 | New molecule |
| NHSL1 | NHS-like 1 | 2.240 | 4.495E-05 | 3.227E-03 | New molecule |
| DGKQ | diacylglycerol kinase, theta 110kDa | 2.241 | 7.741E-07 | 1.205E-04 | New molecule |
| MLK7-AS1 | MLK7 antisense RNA 1 (non-protein coding) | 2.242 | 2.633E-05 | 2.114E-03 | New molecule |
| CLDN1 | claudin 1 | 2.244 | 6.807E-06 | 7.232E-04 | Similar molecule [42] |
| SERPINB5 | serpin peptidase inhibitor, clade B (ovalbumin), member 5 | 2.245 | 9.162E-04 | 3.125E-02 | New molecule |
| F3 | coagulation factor III (thromboplastin, tissue factor) | 2.248 | 5.410E-07 | 8.752E-05 | New molecule |
| PRODH | proline dehydrogenase (oxidase) 1 | 2.253 | 1.613E-05 | 1.446E-03 | New molecule |
| TRIM15 | tripartite motif containing 15 | 2.256 | 4.063E-07 | 6.902E-05 | New molecule |
| SLC19A3 | solute carrier family 19, member 3 | 2.264 | 3.971E-06 | 4.640E-04 | Similar molecule [38] |
| SLC30A4 | solute carrier family 30 (zinc transporter), member 4 | 2.275 | 1.938E-06 | 2.611E-04 | Similar molecule [38] |
| CASP1 | caspase 1, apoptosis-related cysteine peptidase | 2.276 | 1.433E-05 | 1.328E-03 | New molecule |
| CDKN1A | cyclin-dependent kinase inhibitor 1A (p21, Cip1) | 2.277 | 3.010E-05 | 2.361E-03 | New molecule |
| AGAP11 | ankyrin repeat and GTPase domain Arf GTPase activating protein 11 /// family with sequence similarity 25, member A /// family with sequence similarity 25, member B /// family with sequence similarity 25, member C /// family with sequence similarity 25, member G /// family with sequence similarity 25, member H pseudogene | 2.281 | 5.399E-07 | 8.752E-05 | New molecule |
| FAM25A | family with sequence similarity 25 member A | 2.281 | 5.399E-07 | 8.752E-05 | New molecule |
| **Gene symbol** | **Gene name** | **Fold Change ^a^** | **Nominal p-value** | **Adjusted p-value ^b^** | **Previously associated with IM ^c^** |
| FAM25B | family with sequence similarity 25 member B | 2.281 | 5.399E-07 | 8.752E-05 | New molecule |
| FAM25C | family with sequence similarity 25 member C | 2.281 | 5.399E-07 | 8.752E-05 | New molecule |
| FAM25G | family with sequence similarity 25 member G | 2.281 | 5.399E-07 | 8.752E-05 | New molecule |
| FAM25HP | family with sequence similarity 25 member HP | 2.281 | 5.399E-07 | 8.752E-05 | New molecule |
| IGKC | Immunoglobulin kappa constant | 2.283 | 2.403E-05 | 1.968E-03 | New molecule |
| LGALS3 | Lectin, galactoside-binding, soluble, 3 | 2.283 | 1.099E-06 | 1.599E-04 | New molecule |
| HTR1D | 5-hydroxytryptamine (serotonin) receptor 1D, G protein-coupled | 2.283 | 1.394E-06 | 1.956E-04 | New molecule |
| SEMA6A | sema domain, transmembrane domain (TM), and cytoplasmic domain, (semaphorin) 6A | 2.290 | 1.373E-06 | 1.932E-04 | New molecule |
| TPMT | thiopurine S-methyltransferase | 2.292 | 4.905E-06 | 5.562E-04 | New molecule |
| AGMAT | agmatine ureohydrolase (agmatinase) | 2.293 | 3.376E-08 | 8.368E-06 | New molecule |
| NPNT | nephronectin | 2.294 | 2.135E-06 | 2.844E-04 | New molecule |
| ACE | angiotensin I converting enzyme (peptidyl-dipeptidase A) 1 | 2.298 | 4.074E-06 | 4.752E-04 | YES [22] |
| ATP10B | ATPase, class V, type 10B | 2.313 | 2.438E-06 | 3.165E-04 | New molecule |
| YBX2 | Y box binding protein 2 | 2.320 | 1.149E-07 | 2.420E-05 | New molecule |
| IGLL5 | immunoglobulin lambda-like polypeptide 5 | 2.323 | 5.214E-04 | 2.062E-02 | New molecule |
| OVOL1 | ovo-like 1(Drosophila) | 2.334 | 6.615E-08 | 1.503E-05 | New molecule |
| QPRT | quinolinate phosphoribosyltransferase | 2.335 | 1.536E-08 | 4.207E-06 | New molecule |
| GCG | glucagon | 2.336 | 9.696E-04 | 3.252E-02 | New molecule |
| NPY6R | neuropeptide Y receptor Y6 (pseudogene) | 2.342 | 1.080E-04 | 6.430E-03 | New molecule |
| PDXDC2P | pyridoxal-dependent decarboxylase domain containing 2, pseudogene | 2.359 | 8.729E-04 | 3.013E-02 | New molecule |
| ABHD14A | ABHD14A readthrough | 2.363 | 1.265E-07 | 2.634E-05 | New molecule |
| ACY1 | ACY1 readthrough | 2.363 | 1.265E-07 | 2.634E-05 | New molecule |
| HNF4A | hepatocyte nuclear factor 4, alpha | 2.366 | 2.080E-07 | 3.984E-05 | YES [45] |
| CXCL1 | chemokine (C-X-C motif) ligand 1 (melanoma growth stimulating activity, alpha) | 2.368 | 7.747E-04 | 2.780E-02 | YES [11] |
| CEACAM7 | carcinoembryonic antigen-related cell adhesion molecule 7 | 2.369 | 1.053E-03 | 3.442E-02 | Similar molecule [32] |
| TRIM31 | tripartite motif containing 31 | 2.372 | 8.101E-06 | 8.298E-04 | New molecule |
| IL32 | interleukin 32 | 2.373 | 1.068E-07 | 2.283E-05 | New molecule |
| PDK1 | pyruvate dehydrogenase kinase, isozyme 1 | 2.382 | 2.249E-05 | 1.877E-03 | New molecule |
| IGHA1 | immunoglobulin heavy constant | 2.382 | 8.866E-04 | 3.055E-02 | New molecule |
| **Gene symbol** | **Gene name** | **Fold Change ^a^** | **Nominal p-value** | **Adjusted p-value ^b^** | **Previously associated with IM ^c^** |
| IGHD | immunoglobulin heavy constant delta | 2.382 | 8.866E-04 | 3.055E-02 | New molecule |
| IGHG3 | immunoglobulin heavy constant gamma 3 (G3m marker) | 2.382 | 8.866E-04 | 3.055E-02 | New molecule |
| LOC100653245 | ig heavy chain V-III region VH26-like | 2.382 | 8.866E-04 | 3.055E-02 | New molecule |
| IDH3A | isocitrate dehydrogenase 3 (NAD+) alpha | 2.383 | 1.450E-07 | 2.977E-05 | New molecule |
| LOC93432 | maltase-glucoamylase (alpha-glucosidase) pseudogene | 2.387 | 2.352E-07 | 4.430E-05 | New molecule |
| GPRC5A | G protein-coupled receptor, family C, group 5, member A | 2.389 | 5.091E-05 | 3.559E-03 | New molecule |
| NLRP6 | NLR family, pyrin domain containing 6 | 2.389 | 4.863E-07 | 7.957E-05 | New molecule |
| ANXA2 | annexin A2 | 2.391 | 1.596E-06 | 2.190E-04 | New molecule |
| LYN | v-yes-1 Yamaguchi sarcoma viral related oncogene homolog | 2.401 | 4.375E-07 | 7.326E-05 | New molecule |
| SLC39A4 | solute carrier family 39 (zinc transporter), member 4 | 2.412 | 5.428E-07 | 8.755E-05 | Similar molecule [38] |
| EPB41L3 | erythrocyte membrane protein band 4.1-like 3 | 2.413 | 1.346E-04 | 7.564E-03 | New molecule |
| OAT | ornithine aminotransferase | 2.415 | 2.549E-05 | 2.067E-03 | New molecule |
| GGT1 | gamma-glutamyltransferase 1 | 2.437 | 1.508E-05 | 1.375E-03 | New molecule |
| GGT2 | gamma-glutamyltransferase 2 | 2.437 | 1.508E-05 | 1.375E-03 | New molecule |
| GGT3P | gamma-glutamyltransferase 3 pseudogene | 2.437 | 1.508E-05 | 1.375E-03 | New molecule |
| GGT8P | gamma-glutamyltransferase 8 pseudogene | 2.437 | 1.508E-05 | 1.375E-03 | New molecule |
| GRIN2D | glutamate receptor, ionotropic, N-methyl D-aspartate 2D | 2.448 | 1.472E-07 | 3.000E-05 | New molecule |
| GPR128 | G protein-coupled receptor 128 | 2.449 | 1.052E-06 | 1.548E-04 | New molecule |
| PRSS3 | protease, serine, 3 | 2.451 | 2.786E-09 | 9.609E-07 | New molecule |
| OSTBETA | organic solute transporter beta | 2.455 | 7.908E-07 | 1.224E-04 | New molecule |
| GPD1 | glycerol-3-phosphate dehydrogenase 1 (soluble) | 2.462 | 1.249E-08 | 3.506E-06 | New molecule |
| IL2RG | interleukin 2 receptor, gamma | 2.464 | 3.271E-07 | 5.745E-05 | New molecule |
| B3GALT5 | UDP-Gal:betaGlcNAc beta 1,3-galactosyltransferase, polypeptide 5 | 2.466 | 3.289E-05 | 2.521E-03 | New molecule |
| ATP2C2 | ATPase, Ca++ transporting, type 2C, member 2 | 2.469 | 1.713E-07 | 3.394E-05 | New molecule |
| TBX3 | T-box 3 | 2.470 | 2.028E-09 | 7.492E-07 | New molecule |
| FUT6 | fucosyltransferase 6 (alpha (1,3) fucosyltransferase) | 2.475 | 4.809E-07 | 7.895E-05 | Similar molecule [39] |
| SNORD93 | small nucleolar RNA, C/D box 93 | 2.503 | 3.411E-05 | 2.594E-03 | New molecule |
| KHK | ketohexokinase (fructokinase) | 2.504 | 1.046E-07 | 2.252E-05 | New molecule |
| MYO1A | myosin IA | 2.507 | 8.798E-09 | 2.557E-06 | New molecule |
| **Gene symbol** | **Gene name** | **Fold Change a** | **Nominal p-value** | **Adjusted p-value b** | **Previously associated with IM c** |
| IGLC1 | immunoglobulin lambda constant 1 (Mcg marker) | 2.516 | 1.312E-04 | 7.452E-03 | New molecule |
| CLDN15 | claudin 15 | 2.518 | 1.214E-09 | 4.862E-07 | Similar molecule [42] |
| TNFRSF11A | tumor necrosis factor receptor superfamily, member 11a, NFKB activator | 2.521 | 3.587E-08 | 8.778E-06 | New molecule |
| ASS1 | argininosuccinate synthase 1 | 2.529 | 3.313E-06 | 4.036E-04 | New molecule |
| S100A10 | S100 calcium binding protein A10 | 2.535 | 3.844E-06 | 4.511E-04 | New molecule |
| MGAT4A | mannosyl (alpha-1,3-)-glycoprotein beta-1,4-N-acetylglucosaminyltransferase, isozyme A | 2.543 | 1.527E-07 | 3.102E-05 | New molecule |
| TRIM36 | tripartite motif containing 36 | 2.548 | 3.530E-07 | 6.125E-05 | New molecule |
| PAPSS2 | 3'-phosphoadenosine 5'-phosphosulfate synthase 2 | 2.554 | 1.391E-05 | 1.299E-03 | New molecule |
| CEACAM1 | carcinoembryonic antigen-related cell adhesion molecule 1 (biliary glycoprotein) | 2.556 | 3.193E-06 | 3.928E-04 | YES [32] |
| SLC2A5 | solute carrier family 2 (facilitated glucose/fructose transporter), member 5 | 2.559 | 2.263E-06 | 2.973E-04 | Similar molecule [38] |
| VIPR1 | vasoactive intestinal peptide receptor 1 | 2.559 | 4.148E-06 | 4.828E-04 | New molecule |
| CDHR5 | cadherin-related family member 5 | 2.562 | 5.887E-10 | 2.516E-07 | New molecule |
| DAK | dihydroxyacetone kinase 2 homolog (S. cerevisiae) | 2.563 | 3.375E-07 | 5.874E-05 | New molecule |
| SLC3A1 | solute carrier family 3 (cystine, dibasic and neutral amino acid transporters, activator of cystine, dibasic and neutral amino acid transport), member 1 | 2.566 | 1.574E-05 | 1.418E-03 | Similar molecule [38] |
| NR1H4 | nuclear receptor subfamily 1, group H, member 4 | 2.567 | 1.032E-06 | 1.532E-04 | New molecule |
| GSTM4 | glutathione S-transferase mu 4 | 2.577 | 1.021E-06 | 1.518E-04 | YES [46] |
| CDCA3 | cell division cycle associated 3 | 2.578 | 1.181E-07 | 2.477E-05 | New molecule |
| PANK3 | pantothenate kinase 3 | 2.588 | 9.419E-07 | 1.431E-04 | New molecule |
| TINAG | tubulointerstitial nephritis antigen | 2.603 | 4.020E-07 | 6.892E-05 | New molecule |
| SEMA6D | sema domain, transmembrane domain (TM), and cytoplasmic domain, (semaphorin) 6D | 2.625 | 2.383E-05 | 1.955E-03 | New molecule |
| CIDEC | cell death-inducing DFFA-like effector c | 2.632 | 1.544E-05 | 1.403E-03 | New molecule |
| CIDECP | cell death-inducing DFFA-like effector c pseudogene | 2.632 | 1.544E-05 | 1.403E-03 | New molecule |
| PLD1 | phospholipase D1, phosphatidylcholine-specific | 2.641 | 2.365E-06 | 3.095E-04 | New molecule |
| VDR | vitamin D (1,25- dihydroxyvitamin D3) receptor | 2.644 | 3.180E-11 | 1.979E-08 | New molecule |
| MLN | motilin | 2.666 | 4.584E-05 | 3.253E-03 | New molecule |
| LOC653562 | sodium- and chloride-dependent creatine transporter 1-like | 2,667 | 2,523E-09 | 9,088E-07 | New molecule |
| SLC6A10P | solute carrier family 6 (neurotransmitter transporter, creatine), member 10, pseudogene | 2,667 | 2,523E-09 | 9,088E-07 | Similar molecule [38] |
| GLS | glutaminase | 2.668 | 3.099E-07 | 5.511E-05 | New molecule |
| MYO15B | myosin XVB pseudogene | 2.671 | 2.312E-09 | 8.432E-07 | New molecule |
| TRIM40 | tripartite motif containing 40 | 2.677 | 1.287E-04 | 7.379E-03 | New molecule |
| **Gene symbol** | **Gene name** | **Fold Change ^a^** | **Nominal p-value** | **Adjusted p-value ^b^** | **Previously associated with IM ^c^** |
| PHGR1 | proline/histidine/glycine-rich 1 | 2.678 | 4.312E-06 | 4.978E-04 | New molecule |
| CTSZ | cathepsin Z | 2.683 | 1.445E-08 | 4.016E-06 | New molecule |
| ABCG5 | ATP-binding cassette, sub-family G (WHITE), member 5 | 2.684 | 1.719E-05 | 1.519E-03 | New molecule |
| PCK1 | phosphoenolpyruvate carboxykinase 1 (soluble) | 2.690 | 2.654E-05 | 2.128E-03 | New molecule |
| SLC30A10 | solute carrier family 30, member 10 | 2.703 | 1.080E-05 | 1.052E-03 | Similar molecule [38] |
| NAT1 | N-acetyltransferase 1 (arylamine N-acetyltransferase) | 2.708 | 1.288E-06 | 1.840E-04 | New molecule |
| TMEM45B | transmembrane protein 45B | 2.709 | 1.380E-09 | 5.450E-07 | New molecule |
| XDH | xanthine dehydrogenase | 2.713 | 1.692E-08 | 4.562E-06 | New molecule |
| ZNF488 | zinc finger protein 488 | 2.717 | 4.155E-08 | 9.956E-06 | New molecule |
| ATP1A1 | ATPase, Na+/K+ transporting, alpha 1 polypeptide | 2.723 | 9.003E-08 | 1.983E-05 | New molecule |
| CDKN2B | cyclin-dependent kinase inhibitor 2B (p15, inhibits CDK4) | 2.741 | 1.466E-07 | 2.999E-05 | New molecule |
| UGT2A3 | UDP glucuronosyltransferase 2 family, polypeptide A3 | 2.741 | 9.798E-06 | 9.690E-04 | Similar molecule [39] |
| CYBRD1 | cytochrome b reductase 1 | 2.758 | 9.420E-07 | 1.431E-04 | New molecule |
| AFAP1-AS1 | AFAP1 antisense RNA 1 (non-protein coding) | 2.760 | 9.976E-09 | 2.842E-06 | New molecule |
| TMEM25 | transmembrane protein 25 | 2.768 | 1.617E-04 | 8.622E-03 | New molecule |
| ESPN | espin | 2.771 | 2.363E-07 | 4.437E-05 | New molecule |
| SLC22A18AS | solute carrier family 22 (organic cation transporter), member 18 antisense | 2.779 | 1.378E-08 | 3.848E-06 | Similar molecule [38] |
| EPCAM | epithelial cell adhesion molecule | 2.784 | 9.272E-08 | 2.026E-05 | YES [47] |
| REG1B | regenerating islet-derived 1 beta | 2.804 | 3.425E-04 | 1.509E-02 | Similar molecule [22] |
| UGT1A1 | UDP glucuronosyltransferase 1 family, polypeptide A1 | 2.805 | 9.037E-10 | 3.696E-07 | Similar molecule [39] |
| UGT1A10 | UDP glucuronosyltransferase 1 family, polypeptide A10 | 2.805 | 9.037E-10 | 3.696E-07 | Similar molecule [39] |
| UGT1A3 | UDP glucuronosyltransferase 1 family, polypeptide A3 | 2.805 | 9.037E-10 | 3.696E-07 | Similar molecule [39] |
| UGT1A4 | UDP glucuronosyltransferase 1 family, polypeptide A4 | 2.805 | 9.037E-10 | 3.696E-07 | Similar molecule [39] |
| UGT1A5 | UDP glucuronosyltransferase 1 family, polypeptide A5 | 2.805 | 9.037E-10 | 3.696E-07 | Similar molecule [39] |
| UGT1A6 | UDP glucuronosyltransferase 1 family, polypeptide A6 | 2.805 | 9.037E-10 | 3.696E-07 | Similar molecule [39] |
| UGT1A7 | UDP glucuronosyltransferase 1 family, polypeptide A7 | 2.805 | 9.037E-10 | 3.696E-07 | Similar molecule [39] |
| UGT1A8 | UDP glucuronosyltransferase 1 family, polypeptide A8 | 2.805 | 9.037E-10 | 3.696E-07 | Similar molecule [39] |
| UGT1A9 | UDP glucuronosyltransferase 1 family, polypeptide A | 2.805 | 9.037E-10 | 3.696E-07 | Similar molecule [39] |
| BAIAP2L2 | BAI1-associated protein 2-like 2 | 2.811 | 3.476E-11 | 2.136E-08 | New molecule |
| PPP1R14D | protein phosphatase 1, regulatory (inhibitor) subunit 14D | 2.812 | 4.608E-09 | 1.468E-06 | New molecule |
| **Gene symbol** | **Gene name** | **Fold Change ^a^** | **Nominal p-value** | **Adjusted p-value ^b^** | **Previously associated with IM ^c^** |
| CDHR2 | cadherin-related family member 2 | 2.816 | 2.440E-08 | 6.265E-06 | New molecule |
| MTMR11 | myotubularin related protein 11 | 2.816 | 8.241E-08 | 1.842E-05 | New molecule |
| CALML4 | calmodulin-like 4 | 2.819 | 1.784E-06 | 2.427E-04 | New molecule |
| REG1A | regenerating islet-derived 1 alpha | 2.830 | 1.217E-03 | 3.818E-02 | Similar molecule [22] |
| MARVELD3 | MARVEL domain containing 3 | 2.839 | 8.592E-07 | 1.323E-04 | New molecule |
| AQP10 | aquaporin 10 | 2.845 | 1.149E-05 | 1.112E-03 | New molecule |
| IRF4 | interferon regulatory factor 4 | 2.854 | 7.685E-06 | 7.929E-04 | New molecule |
| SLC46A1 | solute carrier family 46 (folate transporter), member 1 | 2.865 | 3.353E-06 | 4.068E-04 | Similar molecule [38] |
| HOXB6 | homeobox B6 | 2.884 | 2.313E-07 | 4.386E-05 | New molecule |
| METTL7B | methyltransferase like 7B | 2.886 | 8.411E-10 | 3.465E-07 | New molecule |
| RNF186 | ring finger protein 186 | 2.908 | 4.646E-07 | 7.690E-05 | New molecule |
| ADH6 | alcohol dehydrogenase 6 (class V) | 2.921 | 3.543E-11 | 2.136E-08 | New molecule |
| USP2 | ubiquitin specific peptidase 2 | 2.945 | 2.368E-09 | 8.582E-07 | New molecule |
| VIL1 | villin 1 | 2.950 | 3.377E-09 | 1.144E-06 | YES [48] |
| CD55 | CD55 molecule, decay accelerating factor for complement (Cromer blood group) | 2.959 | 1.373E-06 | 1.932E-04 | YES [49] |
| ETHE1 | ethylmalonic encephalopathy 1 | 2.968 | 4.811E-07 | 7.895E-05 | New molecule |
| GLRX | glutaredoxin (thioltransferase) | 2.996 | 1.757E-07 | 3.458E-05 | New molecule |
| FAM211A | family with sequence similarity 211, member A | 3.010 | 5.976E-08 | 1.374E-05 | New molecule |
| SLC1A1 | solute carrier family 1 (neuronal/epithelial high affinity glutamate transporter, system Xag), member 1 | 3.022 | 1.832E-06 | 2.480E-04 | Similar molecule [38] |
| HNF4G | hepatocyte nuclear factor 4, gamma | 3.038 | 3.732E-08 | 9.056E-06 | New molecule |
| DEFA6 | defensin, alpha 6, Paneth cell-specific | 3.053 | 1.347E-03 | 4.118E-02 | New molecule |
| CEACAM18 | carcinoembryonic antigen-related cell adhesion molecule 18 | 3.098 | 4.879E-07 | 7.960E-05 | Similar molecule [32] |
| MME | membrane metallo-endopeptidase | 3.100 | 2.723E-06 | 3.443E-04 | New molecule |
| SLC39A5 | solute carrier family 39 (metal ion transporter), member 5 | 3.117 | 4.771E-10 | 2.118E-07 | Similar molecule [38] |
| CDX2 | caudal type homeobox 2 | 3.133 | 4.050E-11 | 2.343E-08 | YES [37] |
| MPP1 | membrane protein, palmitoylated 1, 55kDa | 3.158 | 2.433E-06 | 3.165E-04 | New molecule |
| PDZD3 | PDZ domain containing 3 | 3.158 | 7.432E-09 | 2.194E-06 | New molecule |
| APOBEC1 | apolipoprotein B mRNA editing enzyme, catalytic polypeptide 1 | 3.165 | 2.196E-06 | 2.904E-04 | YES [42] |
| TM6SF2 | transmembrane 6 superfamily member 2 | 3.175 | 1.678E-08 | 4.562E-06 | New molecule |
| KLK1 | kallikrein 1 | 3.179 | 1.316E-09 | 5.234E-07 | New molecule |
| **Gene symbol** | **Gene name** | **Fold Change ^a^** | **Nominal p-value** | **Adjusted p-value ^b^** | **Previously associated with IM ^c^** |
| PIGR | polymeric immunoglobulin receptor | 3.216 | 4.582E-08 | 1.089E-05 | YES [50] |
| CFTR | cystic fibrosis transmembrane conductance regulator (ATP-binding cassette sub-family C, member 7) | 3.225 | 8.028E-11 | 4.296E-08 | YES [51] |
| ACHE | acetylcholinesterase | 3.247 | 2.211E-10 | 1.064E-07 | New molecule |
| SLC6A8 | solute carrier family 6 (neurotransmitter transporter, creatine), member 8 | 3.275 | 2.439E-10 | 1.145E-07 | Similar molecule [38] |
| ACSL5 | acyl-CoA synthetase long-chain family member 5 | 3.276 | 7.154E-08 | 1.613E-05 | New molecule |
| ABP1 | amiloride binding protein 1 (amine oxidase (copper-containing)) | 3.279 | 2.645E-08 | 6.701E-06 | New molecule |
| GSTA1 | glutathione S-transferase alpha 1 | 3.285 | 2.794E-04 | 1.287E-02 | YES [4] |
| HOXA13 | homeobox A13 | 3.294 | 7.134E-05 | 4.632E-03 | New molecule |
| FLVCR2 | feline leukemia virus subgroup C cellular receptor family, member 2 | 3.302 | 4.898E-10 | 2.157E-07 | New molecule |
| LDHA | lactate dehydrogenase A | 3.311 | 1.449E-09 | 5.645E-07 | New molecule |
| TMEM150B | transmembrane protein 150B | 3.316 | 2.081E-11 | 1.370E-08 | New molecule |
| CCL14 | chemokine (C-C motif) ligand 14 | 3.361 | 1.738E-06 | 2.376E-04 | Similar molecule [11] |
| CCL15 | chemokine (C-C motif) ligand 15 | 3.361 | 1.738E-06 | 2.376E-04 | Similar molecule [11] |
| HHLA2 | HERV-H LTR-associating 2 | 3.365 | 7.827E-07 | 1.215E-04 | New molecule |
| TMEM139 | transmembrane protein 139 | 3.386 | 2.229E-10 | 1.064E-07 | New molecule |
| NT5E | 5'-nucleotidase, ecto (CD73) | 3.392 | 9.724E-08 | 2.117E-05 | New molecule |
| FAM84A | family with sequence similarity 84, member A | 3.397 | 1.298E-10 | 6.638E-08 | New molecule |
| BTNL8 | butyrophilin-like 8 | 3.400 | 5.063E-05 | 3.544E-03 | New molecule |
| ANXA13 | annexin A13 | 3.422 | 5.910E-07 | 9.428E-05 | New molecule |
| IGHA1 | immunoglobulin heavy constant alpha 1 | 3,433 | 3,884E-07 | 6,699E-05 | New molecule |
| IGHA2 | immunoglobulin heavy constant alpha 2 (A2m marker) | 3,433 | 3,884E-07 | 6,699E-05 | New molecule |
| IGHG1 | immunoglobulin heavy constant gamma 1 (G1m marker) | 3,433 | 3,884E-07 | 6,699E-05 | New molecule |
| IGHG2 | immunoglobulin heavy constant gamma 2 (G2m marker) | 3,433 | 3,884E-07 | 6,699E-05 | New molecule |
| IGHM | immunoglobulin heavy constant mu | 3,433 | 3,884E-07 | 6,699E-05 | New molecule |
| IGHV4-31 | immunoglobulin heavy variable 4-31 | 3,433 | 3,884E-07 | 6,699E-05 | New molecule |
| ABCG8 | ATP-binding cassette, sub-family G (WHITE), member 8 | 3.436 | 1.341E-07 | 2.771E-05 | New molecule |
| FOLH1 | folate hydrolase (prostate-specific membrane antigen) 1 | 3.451 | 4.451E-06 | 5.119E-04 | New molecule |
| FOLH1B | folate hydrolase (prostate-specific membrane antigen) 1B | 3.451 | 4.451E-06 | 5.119E-04 | New molecule |
| CDA | cytidine deaminase | 3.467 | 2.121E-06 | 2.832E-04 | New molecule |
| TTLL6 | tubulin tyrosine ligase-like family, member 6 | 3.495 | 4.751E-08 | 1.124E-05 | New molecule |
| **Gene symbol** | **Gene name** | **Fold Change ^a^** | **Nominal p-value** | **Adjusted p-value ^b^** | **Previously associated with IM ^c^** |
| MS4A10 | membrane-spanning 4-domains, subfamily A, member 10 | 3.509 | 3.773E-06 | 4.447E-04 | New molecule |
| BCMO1 | beta-carotene 15,15'-monooxygenase 1 | 3.675 | 1.039E-06 | 1.537E-04 | New molecule |
| MSLN | mesothelin | 3.709 | 4.884E-08 | 1.151E-05 | Similar molecule [38] |
| NR1I2 | nuclear receptor subfamily 1, group I, member 2 | 3.709 | 1.035E-09 | 4.201E-07 | New molecule |
| KRT20 | keratin 20 | 3.716 | 2.195E-06 | 2.904E-04 | New molecule |
| MYO7B | myosin VIIB | 3.721 | 1.364E-14 | 2.112E-11 | New molecule |
| SLC35G1 | solute carrier family 35, member G1 | 3.751 | 1.697E-08 | 4.562E-06 | Similar molecule [38] |
| FABP1 | fatty acid binding protein 1, liver | 3.796 | 5.563E-10 | 2.395E-07 | YES [42] |
| PEPD | peptidase D | 3.828 | 5.661E-09 | 1.724E-06 | New molecule |
| GCNT3 | glucosaminyl (N-acetyl) transferase 3, mucin type | 3.832 | 7.866E-11 | 4.250E-08 | New molecule |
| ATP1B3 | ATPase, Na+/K+ transporting, beta 3 polypeptide | 3.908 | 5.972E-10 | 2.533E-07 | New molecule |
| MOGAT3 | monoacylglycerol O-acyltransferase 3 | 3.932 | 4.216E-14 | 5.614E-11 | New molecule |
| GBA3 | glucosidase, beta, acid 3 (cytosolic) | 3.936 | 1.972E-07 | 3.841E-05 | New molecule |
| CLDN3 | claudin 3 | 3.976 | 5.816E-14 | 7.240E-11 | Similar molecule [42] |
| ALPI | alkaline phosphatase, intestinal | 4.018 | 9.756E-09 | 2.793E-06 | New molecule |
| DGKA | diacylglycerol kinase, alpha 80kDa | 4.072 | 1.591E-11 | 1.083E-08 | New molecule |
| CDX1 | caudal type homeobox 1 | 4.147 | 3.523E-09 | 1.187E-06 | YES [52] |
| ABCG2 | ATP-binding cassette, sub-family G (WHITE), member 2 | 4.165 | 2.634E-09 | 9.309E-07 | New molecule |
| REEP6 | receptor accessory protein 6 | 4.175 | 7.111E-11 | 3.916E-08 | New molecule |
| CREB3L3 | cAMP responsive element binding protein 3-like 3 | 4.186 | 4.220E-09 | 1.373E-06 | New molecule |
| SULT1E1 | sulfotransferase family 1E, estrogen-preferring, member 1 | 4.263 | 3.828E-09 | 1.282E-06 | YES [39] |
| CEACAM20 | carcinoembryonic antigen-related cell adhesion molecule 20 | 4.270 | 2.410E-06 | 3.144E-04 | Similar molecule [32] |
| SH3D21 | SH3 domain containing 21 | 4.276 | 1.314E-13 | 1.391E-10 | New molecule |
| SLC5A9 | solute carrier family 5 (sodium/glucose cotransporter), member 9 | 4.327 | 7.422E-09 | 2.194E-06 | Similar molecule [38] |
| HSD17B2 | hydroxysteroid (17-beta) dehydrogenase 2 | 4.328 | 6.133E-09 | 1.848E-06 | Similar molecule [38] |
| VNN1 | vanin 1 | 4.424 | 1.268E-11 | 9.076E-09 | New molecule |
| CLRN3 | clarin 3 | 4.433 | 2.422E-10 | 1.145E-07 | New molecule |
| CAMK2N1 | calcium/calmodulin-dependent protein kinase II inhibitor 1 | 4.446 | 2.560E-09 | 9.105E-07 | New molecule |
| PCK2 | phosphoenolpyruvate carboxykinase 2 (mitochondrial) | 4.467 | 1.498E-08 | 4.125E-06 | New molecule |
| OSTalpha | organic solute transporter alpha | 4.509 | 4.662E-09 | 1.475E-06 | New molecule |
| **Gene symbol** | **Gene name** | **Fold Change ^a^** | **Nominal p-value** | **Adjusted p-value ^b^** | **Previously associated with IM ^c^** |
| CCL25 | chemokine (C-C motif) ligand 25 | 4.608 | 4.333E-09 | 1.395E-06 | Similar molecule [11] |
| ACY3 | aspartoacylase (aminocyclase) 3 | 4.632 | 9.558E-11 | 5.021E-08 | New molecule |
| A1CF | APOBEC1 complementation factor | 4.727 | 1.226E-12 | 1.097E-09 | New molecule |
| SLC4A7 | solute carrier family 4, sodium bicarbonate cotransporter, member 7 | 4.837 | 1.245E-10 | 6.421E-08 | Similar molecule [38] |
| DHRS11 | dehydrogenase/reductase (SDR family) member 11 | 4.996 | 2.182E-13 | 2.231E-10 | New molecule |
| CIDEB | cell death-inducing DFFA-like effector b | 5.051 | 1.012E-11 | 7.428E-09 | New molecule |
| SERPINA1 | serpin peptidase inhibitor, clade A (alpha-1 antiproteinase, antitrypsin), member 1 | 5.069 | 2.542E-09 | 9.099E-07 | New molecule |
| EFNA2 | ephrin-A2 | 5.159 | 7.826E-16 | 2.037E-12 | New molecule |
| CES2 | carboxylesterase 2 | 5.256 | 4.391E-12 | 3.445E-09 | New molecule |
| DPP4 | dipeptidyl-peptidase 4 | 5.275 | 8.173E-09 | 2.388E-06 | New molecule |
| HEPH | hephaestin | 5.282 | 5.134E-11 | 2.940E-08 | New molecule |
| CLDN4 | claudin 4 | 5.283 | 7.801E-15 | 1.314E-11 | Similar molecule [42] |
| GK | glycerol kinase | 5.336 | 3.768E-11 | 2.224E-08 | New molecule |
| SLC15A1 | solute carrier family 15 (oligopeptide transporter), member 1 | 5.371 | 1.599E-06 | 2.190E-04 | Similar molecule [38] |
| GPA33 | glycoprotein A33 (transmembrane) | 5.415 | 2.103E-15 | 4.461E-12 | YES [47] |
| PRAP1 | proline-rich acidic protein 1 | 5.434 | 4.923E-14 | 6.407E-11 | New molecule |
| SPINK4 | serine peptidase inhibitor, Kazal type 4 | 5.492 | 4.420E-13 | 4.364E-10 | New molecule |
| MUC4 | mucin 4, cell surface associated | 5.549 | 1.014E-06 | 1.516E-04 | YES [41] |
| SLC46A3 | solute carrier family 46, member 3 | 5.553 | 1.458E-10 | 7.258E-08 | Similar molecule [38] |
| ABCC13 | ATP-binding cassette, sub-family C (CFTR/MRP), member 13, pseudogene | 5.701 | 1.562E-09 | 6.043E-07 | New molecule |
| TM4SF20 | transmembrane 4 L six family member 20 | 5.719 | 1.362E-10 | 6.901E-08 | New molecule |
| MUC13 | mucin 13, cell surface associated | 5.752 | 5.093E-13 | 4.861E-10 | YES [41] |
| LINC00483 | long intergenic non-protein coding RNA 483 | 5.760 | 1.942E-10 | 9.506E-08 | New molecule |
| RBP2 | retinol binding protein 2, cellular | 5.773 | 1.743E-08 | 4.663E-06 | New molecule |
| GDA | guanine deaminase | 5.803 | 1.746E-10 | 8.619E-08 | New molecule |
| ZG16 | zymogen granule protein 16 homolog (rat) | 5.854 | 2.689E-10 | 1.242E-07 | New molecule |
| XPNPEP2 | X-prolyl aminopeptidase (aminopeptidase P) 2, membrane-bound | 5.952 | 1.408E-09 | 5.524E-07 | New molecule |
| SLC7A9 | solute carrier family 7 (glycoprotein-associated amino acid transporter light chain, bo,+ system), member 9 | 6.007 | 7.421E-11 | 4.047E-08 | Similar molecule [38] |
| ITLN1 | intelectin 1 (galactofuranose binding) | 6.133 | 2.984E-11 | 1.899E-08 | New molecule |
| CEACAM6 | carcinoembryonic antigen-related cell adhesion molecule 6 (non-specific cross reacting antigen) | 6.233 | 2.075E-10 | 1.007E-07 | Similar molecule [32] |
| **Gene symbol** | **Gene name** | **Fold Change ^a^** | **Nominal p-value** | **Adjusted p-value ^b^** | **Previously associated with IM ^c^** |
| GIP | gastric inhibitory polypeptide | 6.249 | 4.603E-10 | 2.075E-07 | New molecule |
| LOC100287119 | MARVEL domain-containing protein 1-like | 6,780 | 2,022E-15 | 4,461E-12 | New molecule |
| MALL | mal, T-cell differentiation protein-like | 6.780 | 2.022E-15 | 4.461E-12 | New molecule |
| APOC3 | apolipoprotein C-III | 6.920 | 4.627E-07 | 7.680E-05 | YES [22] |
| RGS2 | regulator of G-protein signaling 2, 24kDa | 7.026 | 5.487E-11 | 3.080E-08 | New molecule |
| ABCC2 | ATP-binding cassette, sub-family C (CFTR/MRP), member 2 | 7.045 | 5.630E-08 | 1.311E-05 | New molecule |
| APOA1 | apolipoprotein A-I | 7.146 | 4.060E-07 | 6.902E-05 | YES [48] |
| SLC17A4 | solute carrier family 17 (sodium phosphate), member 4 | 7.240 | 3.905E-12 | 3.105E-09 | Similar molecule [38] |
| PLA2G2A | phospholipase A2, group IIA (platelets, synovial fluid) | 7.286 | 2.634E-07 | 4.850E-05 | New molecule |
| CYP3A4 | cytochrome P450, family 3, subfamily A, polypeptide 4 | 7.304 | 4.204E-08 | 1.003E-05 | New molecule |
| APOA4 | apolipoprotein A-IV | 7.331 | 2.059E-08 | 5.385E-06 | YES [48] |
| TM4SF4 | transmembrane 4 L six family member 4 | 7.731 | 2.433E-12 | 1.999E-09 | New molecule |
| LOC100653216 | MAM and LDL-receptor class A domain-containing protein C10orf112-like | 7.780 | 1.164E-11 | 8.440E-09 | New molecule |
| CHP2 | calcineurin-like EF hand protein 2 | 7.989 | 2.291E-14 | 3.330E-11 | New molecule |
| HKDC1 | hexokinase domain containing 1 | 8.030 | 7.256E-16 | 2.031E-12 | New molecule |
| SLC5A1 | solute carrier family 5 (sodium/glucose cotransporter), member 1 | 8.238 | 2.024E-09 | 7.492E-07 | Similar molecule [38] |
| ACE2 | angiotensin I converting enzyme (peptidyl-dipeptidase A) 2 | 8.375 | 1.419E-11 | 9.909E-09 | YES [22] |
| SLC13A2 | solute carrier family 13 (sodium-dependent dicarboxylate transporter), member 2 | 8.412 | 2.326E-14 | 3.330E-11 | Similar molecule [38] |
| SLC6A19 | solute carrier family 6 (neutral amino acid transporter), member 19 | 9.009 | 1.581E-15 | 3.773E-12 | Similar molecule [38] |
| TFF3 | trefoil factor 3 (intestinal) | 9.211 | 6.796E-13 | 6.380E-10 | YES [40] |
| MOGAT2 | monoacylglycerol O-acyltransferase 2 | 9.537 | 2.784E-15 | 5.496E-12 | New molecule |
| ONECUT2 | one cut homeobox 2 | 9.733 | 7.448E-16 | 2.031E-12 | New molecule |
| DEFA5 | defensin, alpha 5, Paneth cell-specific | 10.104 | 2.099E-07 | 4.007E-05 | New molecule |
| REG3A | regenerating islet-derived 3 alpha | 10.840 | 1.871E-06 | 2.527E-04 | Similar molecule [22] |
| BTNL3 | butyrophilin-like 3 | 10.880 | 3.134E-14 | 4.378E-11 | New molecule |
| TMPRSS15 | transmembrane protease, serine 15 | 12.868 | 1.339E-12 | 1.179E-09 | New molecule |
| MUC3A | mucin 3A, cell surface associated | 13.277 | 1.677E-16 | 6.001E-13 | YES [41] |
| MUC3B | mucin 3B | 13.277 | 1.677E-16 | 6.001E-13 | YES [41] |
| MEP1B | meprin A, beta | 13.314 | 5.796E-14 | 7.240E-11 | YES [23] |
| MUC17 | mucin 17, cell surface associated | 15.579 | 1.550E-15 | 3.773E-12 | YES [41] |
| **Gene symbol** | **Gene name** | **Fold Change ^a^** | **Nominal p-value** | **Adjusted p-value ^b^** | **Previously associated with IM ^c^** |
| MUC12 | mucin 12, cell surface associated | 16.382 | 4.336E-09 | 1.395E-06 | YES [41] |
| SLC26A3 | solute carrier family 26, member 3 | 16.877 | 3.539E-11 | 2.136E-08 | Similar molecule [38] |
| CDH17 | cadherin 17, LI cadherin (liver-intestine) | 16.950 | 1.731E-18 | 9.009E-15 | YES [22] |
| REG4 | regenerating islet-derived family, member 4 | 20.286 | 9.460E-14 | 1.022E-10 | YES [22] |
| CPS1 | carbamoyl-phosphate synthase 1, mitochondrial | 22.103 | 1.229E-19 | 1.005E-15 | YES [53] |
| MTTP | microsomal triglyceride transfer protein | 23.334 | 1.176E-14 | 1.871E-11 | YES [54] |
| MUC2 | mucin 2, oligomeric mucus/gel-forming | 24.748 | 5.216E-25 | 1.857E-20 | YES [41] |
| SI | sucrase-isomaltase (alpha-glucosidase) | 25.954 | 2.609E-16 | 8.788E-13 | YES [23] |
| OLFM4 | olfactomedin 4 | 31.586 | 2.242E-19 | 1.426E-15 | YES [35] |
| APOB | apolipoprotein B (including Ag(x) antigen) | 35.139 | 5.209E-15 | 9.479E-12 | YES [22] |
| CLCA1 | chloride channel accessory 1 | 36.602 | 1.360E-17 | 5.989E-14 | New molecule |
| ANPEP | alanyl (membrane) aminopeptidase | 42.976 | 3.479E-24 | 4.981E-20 | New molecule |
| ALDOB | aldolase B, fructose-bisphosphate | 49.964 | 1.428E-18 | 8.176E-15 | YES [42] |
| FABP1 | fatty acid binding protein 1, liver | 67.381 | 7.752E-25 | 1.857E-20 | YES [42] |
| PRDM10 | PR domain containing 10 | 67.381 | 7.752E-25 | 1.857E-20 | YES [42] |
| DMBT1 | deleted in malignant brain tumors 1 | 71.951 | 9.727E-25 | 1.857E-20 | YES [55] |

^a^, Fold Change is the average expression of IM-NoGC/Healthy gastric mucosa, genes are in increasing order by this variable. ^b^, p-value resulting from permutation test (FDR). ^c^, Genes associated with IM by expression studies, genetic association, proteomic or functional studies. "New molecule" means that this is the first time that this gene is identified as differentially expressed in the IM. "Similar molecule" means that another member of the gene family was previously associated with IM.

**References**

1. Magnusson PKE, Enroth H, Eriksson I, et al. Gastric cancer and human leukocyte antigen: distinct DQ and DR alleles are associated with development of gastric cancer and infection by Helicobacter pylori. *Cancer Res.* 2001;61(6):2684-9.

2. Yang S. Gene amplifications at chromosome 7 of the human gastric cancer genome. *Int.J.Mol.Med.* 2007;20:225-231.

3. Cao LX, Le Bousse-Kerdiles MC, Clay D, Oshevski S, Jasmin C, Krief P. Implication of a new molecule IK in CD34+ hematopoietic progenitor cell proliferation and differentiation. *Blood* 1997;89(10):3615-3623.

4. Nguyen T V, Janssen MJR, van Oijen MGH, et al. Genetic polymorphisms in GSTA1, GSTP1, GSTT1, and GSTM1 and gastric cancer risk in a Vietnamese population. *Oncol. Res.* 2010;18(7):349-55.

5. Bai Z, Ye Y, Liang B, et al. Proteomics-based identification of a group of apoptosis-related proteins and biomarkers in gastric cancer. *Int. J. Oncol.* 2011;38(2):375-83.

6. Liu J, Zhang Y, Xu R, et al. PI3K/Akt-dependent phosphorylation of GSK3β and activation of RhoA regulate Wnt5a-induced gastric cancer cell migration. *Cell. Signal.* 2013;25(2):447-56.

7. Li L, Wang L, Song P, et al. Critical role of histone demethylase RBP2 in human gastric cancer angiogenesis. *Mol. Cancer* 2014;13:81.

8. Aquino PF, Fischer JSG, Neves-Ferreira AGC, et al. Are gastric cancer resection margin proteomic profiles more similar to those from controls or tumors? *J. Proteome Res.* 2012;11(12):5836-42.

9. Ikuta K, Seno H, Chiba T. Molecular changes leading to gastric cancer: a suggestion from rare-type gastric tumors with GNAS mutations. *Gastroenterology* 2014;146(5):1417-8.

10. Niiya F, Nishizaka S, Matsunaga K, et al. Expression of SART3 tumor-rejection antigen in gastric cancers. *Japanese J. cancer Res.* 2000;91(3):337-42.

11. Xue X. Abnormal hypermethylation of promoter region downregulates chemokine CXC ligand 14 expression in gastric cancer. *Int. J. Oncol.* 2013;43(5):1487-94.

12. Kalnina Z, Silina K, Bruvere R, et al. Molecular characterisation and expression analysis of SEREX-defined antigen NUCB2 in gastric epithelium, gastritis and gastric cancer. *Eur. J. Histochem.* 2009;53(1):2.

13. Wu Q, Yang Z, Xia L, et al. Methylation of miR-129-5p CpG island modulates multi-drug resistance in gastric cancer by targeting ABC transporters. *Oncotarget* 2014;5(22):11552-63.

14. Ueda Y, Ishikawa K, Shiraishi N, Yokoyama S, Kitano S. Clinical significance of HLA class I heavy chain expression in patients with gastric cancer. *J. Surg. Oncol.* 2008;97(5):451-455.

15. Qian Z, Zhu G, Tang L, et al. Whole genome gene copy number profiling of gastric cancer identifies PAK1 and KRAS gene amplification as therapy targets. *Genes. Chromosomes Cancer* 2014;53(11):883-94.

16. Li S, Lu A-P, Zhang L, Li Y-D. Anti-Helicobacter pylori immunoglobulin G (IgG) and IgA antibody responses and the value of clinical presentations in diagnosis of H. pylori infection in patients with precancerous lesions. *World J. Gastroenterol.* 2003;9(4):755-758.

17. Nam KH, Lee BL, Park JH, et al. Caveolin 1 expression correlates with poor prognosis and focal adhesion kinase expression in gastric cancer. *Pathobiology* 2013;80(2):87-94.

18. Watanabe Y, Aoyama N, Sakai T, et al. HLA-DQB1 locus and gastric cancer in Helicobacter pylori infection. *J. Gastroenterol. Hepatol.* 2006;21(2):420-424.

19. Wang Y-C, Wang J-L, Kong X, et al. CD24 mediates gastric carcinogenesis and promotes gastric cancer progression via STAT3 activation. *Apoptosis* 2014;19(4):643-656.

20. Xu H-Y, Chen Z-W, Pan Y-M, Fan L, Guan J, Lu Y-Y. Transfection of PDCD5 Effect on the Biological Behavior of Tumor Cells and Sensitized Gastric Cancer Cells to Cisplatin-Induced Apoptosis. *Dig. Dis. Sci.* 2012;57(7):1847-1856.

21. Kamangar F, Cheng C, Abnet CC, Rabkin CS. Interleukin-1B polymorphisms and gastric cancer risk--a meta-analysis. *Cancer Epidemiol. Biomarkers Prev.* 2006;15(10):1920-1928.

22. Lee HJ, Nam KT, Park HS, et al. Gene Expression Profiling of Metaplastic Lineages Identifies CDH17 as a Prognostic Marker in Early Stage Gastric Cancer. *Gastroenterology* 2010;139(1):358-366.

23. Kim KR, Oh SY, Park UC, et al. Gene expression profiling using oligonucleotide microarray in atrophic gastritis and intestinal metaplasia. *Korean J. Gastroenterol.* 2007;49(4):209-24.

24. Xie Y, Wang Y, Zhao Y, Guo Z. Single-nucleotide polymorphisms of microRNA processing machinery genes are associated with risk for gastric cancer. *Onco. Targets. Ther.* 2015;8:567-571.

25. Wang J, Cui S, Zhang X, Wu Y, Tang H. High expression of heat shock protein 90 is associated with tumor aggressiveness and poor prognosis in patients with advanced gastric cancer. *PLoS One* 2013;8(4):e62876.

26. Humphries JM, Penno MAS, Weiland F, et al. Identification and validation of novel candidate protein biomarkers for the detection of human gastric cancer. *Biochim. Biophys. Acta - Proteins Proteomics* 2014;1844(5):1051-1058.

27. Wu W, Juan WC, Liang CRMY, Yeoh KG, So J, Chung MCM. S100A9, GIF and AAT as potential combinatorial biomarkers in gastric cancer diagnosis and prognosis. *PROTEOMICS - Clin. Appl.* 2012;6(3-4):152-162.

28. Terashima M, Maesawa C, Oyama K, et al. Gene expression profiles in human gastric cancer: expression of maspin correlates with lymph node metastasis. *Br. J. Cancer* 2005;92(6):1130-1136.

29. Dai J, Zhang N, Wang J, Chen M, Chen J. Gastrokine-2 is downregulated in gastric cancer and its restoration suppresses gastric tumorigenesis and cancer metastasis. *Tumor Biol.* 2014;35(5):4199-4207.

30. Nardone G, Martin G, Rocco A, et al. Molecular expression of Gastrokine 1 in normal mucosa and in Helicobacter pylori-related preneoplastic and neoplastic gastric lesions. *Cancer Biol. Ther.* 2008;7(12):1890-1895.

31. Ning PF, Liu HJ, Yuan Y. Dynamic expression of pepsinogen C in gastric cancer, precancerous lesions and Helicobacter pylori associated gastric diseases. *World J. Gastroenterol.* 2005;11(17):2545-2548.

32. Shi J, Xu S, He P, Xi Z. Expression of carcinoembryonic antigen-related cell adhesion molecule 1(CEACAM1) and its correlation with angiogenesis in gastric cancer. *Pathol. - Res. Pract.* 2014;210(8):473-476.

33. Li X, Zhang Y, Zhang H, et al. miRNA-223 Promotes Gastric Cancer Invasion and Metastasis by Targeting Tumor Suppressor EPB41L3. *Mol. Cancer Res.* 2011;9(7):824-833.

34. Han Y, Tu W-W, Wen Y-G, et al. Identification and validation that up-expression of HOXA13 is a novel independent prognostic marker of a worse outcome in gastric cancer based on immunohistochemistry. *Med. Oncol.* 2013;30(2):564.

35. Jang BG, Lee BL, Kim WH. Olfactomedin-related proteins 4 (OLFM4) expression is involved in early gastric carcinogenesis and of prognostic significance in advanced gastric cancer. *Virchows Arch.* 2015;467(3):285-294.

36. Shafaghi A, Mansour-Ghanaei F, Joukar F, et al. Serum Gastrin and the Pepsinogen I/II Ratio as Markers for Diagnosis of Premalignant Gastric Lesions. *Asian Pacific J. Cancer Prev.* 2013;14(6):3931-3936.

37. Hayakawa Y, Fox JG, Gonda T, Worthley DL, Muthupalani S, Wang TC. Mouse models of gastric cancer. *Cancers (Basel).* 2013;5(1):92-130.

38. Ng EKO, Leung CPH, Shin VY, et al. Quantitative Analysis and Diagnostic Significance of Methylated SLC19A3 DNA in the Plasma of Breast and Gastric Cancer Patients. *PLoS One* 2011;6(7):e22233.

39. Chandrasekaran E V, Xue J, Piskorz C, et al. Potential tumor markers for human gastric cancer: an elevation of glycan:sulfotransferases and a concomitant loss of α1,2-fucosyltransferase activities. *J. Cancer Res. Clin. Oncol.* 2007;133(9):599-611.

40. Kim BW, Kim KM, Lee BI, et al. Expression of trefoil peptides in the subtypes of intestinal metaplasia. *Peptides* 2004;25(5):779-783.

41. de Bolos C, Real FX, Lopez-Ferrer A. Regulation of mucin and glycoconjugate expression: from normal epithelium to gastric tumors. *Front. Biosci.* 2001;6:D1256-D1263.

42. Chen X. Variation in Gene Expression Patterns in Human Gastric Cancers. *Mol. Biol. Cell* 2003;14(8):3208-3215.

43. Tanaka H, Tsukamoto T, Mizoshita T, et al. Expression of small intestinal and colonic phenotypes in complete intestinal metaplasia of the human stomach. *Virchows Arch.* 2005;447(5):806-815.

44. Nie XC, Wang JP, Zhu W, et al. COL4A3 expression correlates with pathogenesis, pathologic behaviors, and prognosis of gastric carcinomas. *Hum. Pathol.* 2013;44(1):77-86.

45. Mesquita P, Raquel A, Nuno L, et al. Metaplasia--a transdifferentiation process that facilitates cancer development: the model of gastric intestinal metaplasia. *Crit. Rev. Oncog.* 2006;12(1-2):3-26.

46. Chen SY, Liu TY, Shun CT, et al. Modification effects of GSTM1, GSTT1 and CYP2E1 polymorphisms on associations between raw salted food and incomplete intestinal metaplasia in a high-risk area of stomach cancer. *Int. J. Cancer* 2004;108(4):606-612.

47. Joo M, Kim H, Kim MK, Yu HJ, Kim JP. Expression of Ep-CAM in intestinal metaplasia, gastric epithelial dysplasia and gastric adenocarcinoma. *J. Gastroenterol. Hepatol.* 2005;20(7):1039-1045.

48. Gomes LI, Esteves GH, Carvalho AF, et al. Expression Profile of Malignant and Nonmalignant Lesions of Esophagus and Stomach: Differential Activity of Functional Modules Related to Inflammation and Lipid Metabolism. *Cancer Res.* 2005;65(16):7127-7136.

49. Hensel F, Timmermann W, von Rahden BH, Rosenwald A, Brändlein S IB. Ten-year follow-up of a prospective trial for the targeted therapy of gastric cancer with the human monoclonal antibody PAT-SC1. *Oncol. Rep.* 2014;31(3):1059-1066.

50. Fristedt R, Gaber A, Hedner C, et al. Expression and prognostic significance of the polymeric immunoglobulin receptor in esophageal and gastric adenocarcinoma. *J. Transl. Med.* 2014;12:83.

51. Weis VG, Sousa JF, LaFleur BJ, et al. Heterogeneity in mouse spasmolytic polypeptide-expressing metaplasia lineages identifies markers of metaplastic progression. *Gut* 2013;62(9):1270-1279.

52. Boussioutas A, Li H, Liu J, et al. Distinctive patterns of gene expression in premalignant gastric mucosa and gastric cancer. *Cancer Res.* 2003;63(10):2569-77.

53. Liu TH, Li DC, Gu CF, Ye SF. Carbamyl phosphate synthetase I. A novel marker for gastric carcinoma. *Chin. Med. J. (Engl).* 1989;102(8):630-8.

54. Correa P, Piazuelo MB. The gastric precancerous cascade. *J. Dig. Dis.* 2012;13(1):2-9.

55. Sousa JF, Ham AJL, Whitwell C, et al. Proteomic profiling of paraffin-embedded samples identifies metaplasia-specific and early-stage gastric cancer biomarkers. *Am. J. Pathol.* 2012;181(5):1560-1572.
