# Supplementary material for: Gene expression study and pathway analysis of histological subtypes of intestinal metaplasia that progress to gastric cancer
Source: PLoS One. 2017 Apr 25;12(4):e0176043. doi: 10.1371/journal.pone.0176043 (PMC5404762; doi:10.1371/journal.pone.0176043)
Supplement: S9 Table — (DOC) [file pone.0176043.s011.doc]

**S9 Table.** GSEA analysis in IIM-GC from c2all.v5 and c3tft.v5 catalogs

| **Molecular processes** | **# of up-regulated gene sets ^a^** |
| --- | --- |
| Cell cycle and cell proliferation | 24 |
| Oncogenes | 20 |
| Tumor suppresors | 17 |
| Invasion and metastasis | 15 |
| Inflammation | 13 |
| Adipogenesis | 8 |
| Presentation and antigen processing | 7 |
| Apoptosis | 6 |
| Angiogenesis | 5 |
| Oxidative Phosphorylation | 5 |
| Cell adhesion | 4 |
| Proteasomal degradation | 4 |
| Insulin signalling | 3 |
| Response to genomic damage | 3 |
| Endocitosis or fagocitosis | 2 |
| Esophageal cancer | 2 |
| Unfolded protein response | 1 |
| Gastric cancer | 1 |
| Nonsense mediated decay | 1 |
| E2F family (c3tft.v5) | 19 |

^a^ Number of significant upregulated gene sets after categorization in molecular processes according to their function
