# Supplementary material for: Gene expression study and pathway analysis of histological subtypes of intestinal metaplasia that progress to gastric cancer
Source: PLoS One. 2017 Apr 25;12(4):e0176043. doi: 10.1371/journal.pone.0176043 (PMC5404762; doi:10.1371/journal.pone.0176043)
Supplement: S10 Table — (DOC) [file pone.0176043.s012.doc]

**S10 Table.** GSEA analysis in IM-NoGC from c2all.v5 and c3tft.v5 catalogs

| **Molecular processes** | **# of up-regulated gene sets ^a^** |
| --- | --- |
| Warburg effect | 20 |
| Lipid metabolism | 15 |
| Cell proliferation | 14 |
| Inflammation | 13 |
| Intestinal differentiation | 9 |
| Presentation and antigen processing | 8 |
| Apoptosis | 7 |
| Oncogenes | 7 |
| Response to genomic damage | 4 |
| Tumor suppresors | 4 |
| Angiogenesis | 3 |
| Gastric cancer | 3 |
| Invasion and metastasis | 3 |
| Xenobiotic metabolism | 3 |
| Aberrant protein glycosylation | 2 |
| Gastroesophageal reflux | 2 |
| *H.pylori* infection | 1 |
| Unfolded protein response | 1 |
| Oxidative Phosphorylation | 1 |
| HNF1/4 and GATA1/6 (c3tft.v5) | 12 |

^a^ Number of significant upregulated gene sets after categorization in molecular processes according to their function
