# Supplementary material for: Gene expression study and pathway analysis of histological subtypes of intestinal metaplasia that progress to gastric cancer
Source: PLoS One. 2017 Apr 25;12(4):e0176043. doi: 10.1371/journal.pone.0176043 (PMC5404762; doi:10.1371/journal.pone.0176043)
Supplement: S11 Table — (DOC) [file pone.0176043.s013.doc]

**S11 Table.** Over-expressed gene sets in IIM-GC with extreme values of *Rank at max* parameter

| **Gene sets** | **Molecular processes** | **ES ^a^** | **Nominal p-value ^b^** | **q-value FDR ^c^** | **Rank at max ^d^** |
| --- | --- | --- | --- | --- | --- |
| REACTOME_G_BETA_GAMMA_SIGNALLING_THROUGH_PI3KGAMMA | Cell cycle and cell proliferation | 0.593 | 0.007 | 0.024 | 895 |
| BERENJENO_TRANSFORMED_BY_RHOA_FOREVER_DN | Oncogenes | 0.627 | 0.000 | 0.002 | 1908 |
| YU_MYC_TARGETS_UP | Oncogenes | 0.512 | 0.003 | 0.016 | 2259 |
| SCHOEN_NFKB_SIGNALING | Inflammation | 0.517 | 0.002 | 0.027 | 2552 |
| PEDERSEN_METASTASIS_BY_ERBB2(her2)_ISOFORM_1 | Invasion and metastasis | 0.489 | 0.005 | 0.027 | 2804 |
| CROONQUIST_NRAS_SIGNALING_UP | Oncogenes | 0.589 | 0.000 | 0.003 | 2872 |
| SCIAN_CELL_CYCLE_TARGETS_OF_TP53_AND_TP73_DN | Tumor Supressors | 0.598 | 0.004 | 0.018 | 2879 |
| SARTIPY_NORMAL_AT_INSULIN_RESISTANCE_UP | Insulin regulated genes | 0.646 | 0.000 | 0.001 | 2898 |
| ASTIER_INTEGRIN_SIGNALING | Cell cycle and cell proliferation | 0.449 | 0.003 | 0.031 | 3343 |
| PEDERSEN_METASTASIS_BY_ERBB2_ISOFORM_4 | Invasion and metastasis | 0.419 | 0.000 | 0.023 | 3368 |
| REN_BOUND_BY_E2F | Cell cycle and cell proliferation | 0.571 | 0.000 | 0.001 | 3374 |
| ZHOU_CELL_CYCLE_GENES_IN_IR_RESPONSE_6HR | Response to genoCIM damage | 0.423 | 0.003 | 0.032 | 3429 |
| MARKEY_RB1_ACUTE_LOF_UP | Tumor Supressors | 0.369 | 0.000 | 0.037 | 3592 |
| MARZEC_IL2_SIGNALING_UP | Inflammation | 0.437 | 0.000 | 0.016 | 3796 |
| PID_INTEGRIN5_PATHWAY | Cell Adhesion | 0.631 | 0.003 | 0.018 | 3850 |
| LU_TUMOR_ANGIOGENESIS_UP | Angiogenesis | 0.564 | 0.002 | 0.034 | 3855 |
| PHONG_TNF_RESPONSE_VIA_P38_PARTIAL | Inflammation | 0.366 | 0.002 | 0.050 | 3878 |
| ZHOU_CELL_CYCLE_GENES_IN_IR_RESPONSE_24HR | Response to genoCIM damage | 0.429 | 0.000 | 0.014 | 3976 |
| WANG_TUMOR_INVASIVENESS_UP | Invasion and metastasis | 0.420 | 0.000 | 0.002 | 6767 |
| ST_INTEGRIN_SIGNALING_PATHWAY | Cell Adhesion | 0.398 | 0.006 | 0.048 | 6779 |
| GOTZMANN_EPITHELIAL_TO_MESENCHYMAL_TRANSITION_DN | Invasion and metastasis | 0.375 | 0.000 | 0.026 | 6789 |
| REACTOME_CELL_CYCLE_MITOTIC | Cell cycle and cell proliferation | 0.382 | 0.000 | 0.011 | 6864 |
| KEGG_CELL_CYCLE | Cell cycle and cell proliferation | 0.419 | 0.000 | 0.018 | 6872 |
| THEILGAARD_NEUTROPHIL_AT_SKIN_WOUND_DN | Inflammation | 0.379 | 0.000 | 0.019 | 6872 |
| WELCSH_BRCA1_TARGETS_UP | Tumor Supressors | 0.392 | 0.000 | 0.016 | 6907 |
| WELCSH_BRCA1_TARGETS_DN | Tumor Supressors | 0.424 | 0.000 | 0.014 | 6946 |
| CHANDRAN_METASTASIS_UP | Invasion and metastasis | 0.374 | 0.000 | 0.027 | 6995 |
| APRELIKOVA_BRCA1_TARGETS | Tumor Supressors | 0.501 | 0.000 | 0.016 | 7075 |
| **Genesets** | **Molecular processes** | **ES ^a^** | **Nominal p-value ^b^** | **q-value FDR ^c^** | **Rank at max ^d^** |
| KEGG_OXIDATIVE_PHOSPHORYLATION | Oxidative Phosphorylation | 0.476 | 0.000 | 0.003 | 7229 |
| ZHOU_TNF_SIGNALING_30MIN | Inflammation | 0.452 | 0.002 | 0.034 | 7334 |
| KEGG_LYSOSOME | Endocitosis or Fagocitosis | 0.404 | 0.000 | 0.022 | 7368 |
| REACTOME_NONSENSE_MEDIATED_DECAY_ENHANCED_BY_THE_EXON_JUNCTION_COMPLEX | Nonsense mediated decay | 0.455 | 0.000 | 0.009 | 7378 |
| REACTOME_RESPIRATORY_ELECTRON_TRANSPORT_ATP_SYNTHESIS_BY_CHEMIOSMOTIC_COUPLING_AND_HEAT_PRODUCTION_BY_UNCOUPLING_PROTEINS_ | Oxidative Phosphorylation | 0.511 | 0.000 | 0.002 | 7589 |
| REACTOME_TCA_CYCLE_AND_RESPIRATORY_ELECTRON_TRANSPORT | Oxidative Phosphorylation | 0.473 | 0.000 | 0.003 | 7589 |
| BURTON_ADIPOGENESIS_2 | Lipid Metabolism | 0.440 | 0.000 | 0.023 | 7604 |
| REACTOME_RESPIRATORY_ELECTRON_TRANSPORT | Oxidative Phosphorylation | 0.517 | 0.000 | 0.003 | 8038 |

a Enrichment score. b p-value of gene sets, unadjusted for multiple corrections. c q-value of gene sets, adjusted by multiple corrections (FDR). d Position in the ranking list at which the highest value of ES is obtained
