# Supplementary material for: Gene expression study and pathway analysis of histological subtypes of intestinal metaplasia that progress to gastric cancer
Source: PLoS One. 2017 Apr 25;12(4):e0176043. doi: 10.1371/journal.pone.0176043 (PMC5404762; doi:10.1371/journal.pone.0176043)
Supplement: S12 Table — (DOC) [file pone.0176043.s014.doc]

**S12 Table.** Gene sets composed by at least 3 leading edge genes up-regulated in the IIM-GC

| **Gene sets ^a^** | **Molecular processes ^b^** | **ES ^c^** | **Nominal p-value ^d^** | **q-value FDR ^e^** | **Over-expressed *leading edge*  genes in IIM-GC ^f^** | **Rank at max ^g^** |
| --- | --- | --- | --- | --- | --- | --- |
| IGLESIAS_E2F_TARGETS_UP | Cell cycle and cell proliferation | 0.418 | 0.000 | 0.012 | *CD53, HSP90AA1, MORF4L1, C3, RBBP7, LAPTM5* | 5813 |
| CHICAS_RB1_TARGETS_CONFLUENT | Tumor supressors | 0.361 | 0.000 | 0.013 | *CLU, ATP6V0E1, CAV1, EIF5B, MYOF, C1R* | 4907 |
| GOLDRATH_ANTIGEN_RESPONSE | Presentation and antigenic processing | 0.42 | 0.000 | 0.002 | *HLA-A, C3, CD24, RAN* | 6476 |
| LABBE_WNT3A_TARGETS_UP | Cell cycle and cell proliferation | 0.39 | 0.006 | 0.045 | *RBBP7, CAV1, FABP5, CD24* | 6333 |
| CHICAS_RB1_TARGETS_GROWING | Tumor supressors |  | 0.000 | 0.003 | *CLU, C1R, CAV1, FABP5* | 4697 |
| ROME_INSULIN_TARGETS_IN MUSCLE_UP | Insulin regulated genes | 0.417 | 0.000 | 0.002 | *HLA-C, KARS****,***  *CCT6A* | 6051 |
| BERENJENO_TRANSFORMED_BY_ RHOA_UP | Oncogenes | 0.428 | 0.000 | 0.001 | *CAV, CD24, C1R* | 4980 |
| REACTOME_CELL_CYCLE | Cell cycle and cell proliferation | 0.355 | 0.000 | 0.025 | *NHP2, HSP90AA1, RBBP7* | 5615 |
| SANA_TNF_SIGNALING_DN | Inflammation | 0.417 | 0.006 | 0.039 | *ATP5A1, CLU, MYOF* | 5458 |
| MENSSEN_MYC_TARGETS | Oncogenes | 0.615 | 0.000 | 0.001 | *HSP90AB1, C1QBP, HSP90AA1* | 5289 |
| HEDENFALK_BREAST_CANCER BRCA1_VS_BRCA2 | Tumor supressors | 0.424 | 0.000 | 0.008 | *PDCD5, CCT6A, FABP5*  *,* | 5652 |
| ALONSO_METASTASIS_UP | Invasion and metastasis | 0.477 | 0.000 | 0.001 | *NHP2, CAV1, RAN* | 5250 |
| WANG_ESOPHAGUS_CANCER_VS_NORMAL_UP | Esophageal cancer | 0.477 | 0.000 | 0.015 | *HLA-A, C1R, LAPTM5* | 5578 |

a Over-expressed gene sets in the IIM-GC. b Functional process represented by genesets. c Enrichment score. d p-value of gene sets, unadjusted for multiple corrections. e q-value of gene sets, adjusted by FDR multiple corrections test. f Leading edge genes over-expressed in the IIM-GC. g Position in the ranking list at which the highest value of ES is obtained.
