# Supplementary material for: Gene expression study and pathway analysis of histological subtypes of intestinal metaplasia that progress to gastric cancer
Source: PLoS One. 2017 Apr 25;12(4):e0176043. doi: 10.1371/journal.pone.0176043 (PMC5404762; doi:10.1371/journal.pone.0176043)
Supplement: S13 Table — (DOC) [file pone.0176043.s015.doc]

**S13Table.** Over-expressed genesets in IM-NoGC with extreme values of *Rank at max* parameter

| **Gene sets** | | | | **Molecular processes** | | | **ES ^a^** | **Nominal p-value ^b^** | **q-value FDR ^c^** | **Rank at max ^d^** |
| --- | --- | --- | --- | --- | --- | --- | --- | --- | --- | --- |
| REACTOME_CHYLOCIMRON_MEDIATED_LIPID_TRANSPORT | | | | Lipid Metabolism | | | 0.843 | 0.000 | 0.157 | 86 |
| REACTOME_LIPOPROTEIN_METABOLISM | | | | Lipid Metabolism | | | 0.748 | 0.000 | 0.123 | 86 |
| REACTOME_ABCA_TRANSPORTERS_IN_LIPID_HOMEOSTASIS | | | | Lipid Metabolism | | | 0.683 | 0.009 | 0.202 | 167 |
| REACTOME_TERMINATION_OF_O_GLYCAN_BIOSYNTHESIS | | | | Aberrant protein glycosylation | | | 0.716 | 0.002 | 0.144 | 210 |
| WANG_NEOPLASTIC_TRANSFORMATION_BY_CCND1_MYC | | | | Oncogenes | | | 0.570 | 0.002 | 0.191 | 284 |
| DAZARD_UV_RESPONSE_CLUSTER_G24 | | | | Response to genoCIM damage | | | 0.671 | 0.000 | 0.152 | 312 |
| LIU_CDX2_TARGETS_UP | | | | Intestinal differentiation | | | 0.738 | 0.000 | 0.193 | 385 |
| REACTOME_LIPID_DIGESTION_MOBILIZATION_AND_TRANSPORT | | | | Lipid Metabolism | | | 0.665 | 0.000 | 0.157 | 396 |
| WOTTON_RUNX_TARGETS_DN | | | | Tumor supressors | | | 0.624 | 0.008 | 0.201 | 437 |
| MOOTHA_GLUCONEOGENESIS | | | | Non tumoral Warburg Effect | | | 0.651 | 0.000 | 0.168 | 527 |
| MCMURRAY_TP53_HRAS_COOPERATION_RESPONSE_UP | | | | Tumor supressors | | | 0.533 | 0.022 | 0.241 | 633 |
| GENTILE_UV_HIGH_DOSE_UP | | | | Response to genoCIM damage | | | 0.605 | 0.021 | 0.170 | 661 |
| WANG_BARRETTS_ESOPHAGUS_UP | | | | Intestinal differentiation | | | 0.744 | 0.000 | 0.144 | 829 |
| PID_IL8CXCR1_PATHWAY | | | | Inflammation | | | 0.662 | 0.000 | 0.140 | 860 |
| BAUS_TFF2_TARGETS_UP | | | | Antigenic processing | | | 0.789 | 0.000 | 0.167 | 952 |
| PID_AMB2_NEUTROPHILS_PATHWAY | | | | Inflammation | | | 0.498 | 0.014 | 0.241 | 1044 |
| MURATA_VIRULENCE_OF_H_PILORI | | | | H.pylori infection | | | 0.669 | 0.002 | 0.137 | 1081 |
| KANNAN_TP53_TARGETS_UP | | | | Tumor supressors | | | 0.439 | 0.019 | 0.242 | 1165 |
| KEGG_ETHER_LIPID_METABOLISM | | | | Lipid Metabolism | | | 0.532 | 0.029 | 0.246 | 1240 |
| BROWN_MYELOID_CELL_DEVELOPMENT_UP | | | | Inflammation | | | 0.422 | 0.014 | 0.240 | 4270 |
| REACTOME_PHOSPHOLIPID_METABOLISM | | | | Lipid Metabolism | | | 0.425 | 0.002 | 0.161 | 4273 |
| GENTILE_UV_LOW_DOSE_UP | | | | Response to genoCIM damage | | | 0.682 | 0.000 | 0.138 | 4283 |
| AMIT_EGF_RESPONSE_120_HELA | | | | Cell Proliferation | | | 0.425 | 0.023 | 0.241 | 4300 |
| ZUCCHI_METASTASIS_DN | | | | Invasion and metastasis | | | 0.505 | 0.039 | 0.236 | 4466 |
| REACTOME_RIP_MEDIATED_NFKB_ACTIVATION_VIA_DAI | | | | Inflammation | | | 0.621 | 0.032 | 0.242 | 4586 |
| REACTOME_TRAF6_MEDIATED_NFKB_ACTIVATION | | | | Inflammation | | | 0.569 | 0.016 | 0.241 | 4586 |
|  |  |  |  | |  |  | | | | |
| **Gene sets** | | | | **Molecular processes** | | | **ES ^a^** | **Nominal p-value ^b^** | **q-value FDR ^c^** | **Rank at max ^d^** |
| KEGG_BIOSYNTHESIS_OF_UNSATURATED_FATTY_ACIDS | | | | Lipid Metabolism | | | 0.630 | 0.019 | 0.200 | 4792 |
| BIOCARTA_MITOCHONDRIA_PATHWAY | | | | Apoptosis | | | 0.682 | 0.002 | 0.167 | 4809 |
| KEGG_B_CELL_RECEPTOR_SIGNALING_PATHWAY | | | | Antigenic presentation and processing | | | 0.515 | 0.004 | 0.169 | 4873 |
| NEMETH_INFLAMMATORY_RESPONSE_LPS_UP | | | | Inflammation | | | 0.513 | 0.032 | 0.204 | 4873 |
| KEGG_APOPTOSIS | | | | Apoptosis | | | 0.492 | 0.000 | 0.138 | 4882 |
| PID_P53DOWNSTREAMPATHWAY | | | | Tumor supressors | | | 0.429 | 0.004 | 0.158 | 4936 |
| KEGG_T_CELL_RECEPTOR_SIGNALING_PATHWAY | | | | Antigenic presentation and processing | | | 0.436 | 0.004 | 0.159 | 5026 |
| REACTOME_INTRINSIC_PATHWAY_FOR_APOPTOSIS | | | | Apoptosis | | | 0.613 | 0.006 | 0.172 | 5120 |
| ACOSTA_PROLIFERATION_INDEPENDENT_MYC_TARGETS_DN | | | | Oncogenes | | | 0.395 | 0.020 | 0.199 | 5253 |
| PID_IL12_2PATHWAY | | | | Inflammation | | | 0.479 | 0.036 | 0.239 | 5464 |
| PID_PDGFRBPATHWAY | | | | Angiogenesis | | | 0.418 | 0.030 | 0.235 | 5748 |
| REACTOME_TRAF6_MEDIATED_IRF7_ACTIVATION | | | | Inflammation | | | 0.543 | 0.009 | 0.203 | 6196 |
| DANG_MYC_TARGETS_DN | | | | Oncogenes | | | 0.519 | 0.019 | 0.204 | 7011 |

a Enrichment score. b Nominal p-value of gene sets, unadjusted for multiple corrections. c q-value of gene sets, adjusted by FDR multiple corrections test. d Position in the ranking list at which the highest value of ES is obtained
