# Supplementary material for: Gene expression study and pathway analysis of histological subtypes of intestinal metaplasia that progress to gastric cancer
Source: PLoS One. 2017 Apr 25;12(4):e0176043. doi: 10.1371/journal.pone.0176043 (PMC5404762; doi:10.1371/journal.pone.0176043)
Supplement: S14 Table — (DOC) [file pone.0176043.s016.doc]

**S14 Table.** Gene sets composed by at least 3 leading edge genes over-expressed in the IM-NoGC

| **Gene sets ^a^** | **Molecular processes ^b^** | **ES ^c^** | **Nominal p-value ^d^** | **q-value FDR ^e^** | **Over-expressed leading edge genes in IIM-GC ^f^** | **Rank at max ^g^** |
| --- | --- | --- | --- | --- | --- | --- |
| BILD_HRAS_ONCOGENIC_SIGNATURE | Oncogenes | 0.392 | 0.032 | 0.242 | *TRIB1, ELOVL7, AGPAT9, ACOT7, FGFBP1, PI3, CXCL1, SLC25A37, SERPINB5, TBX3, LYN, CD55, NT5E, TMEM45B, GK* | 2504 |
| VECCHI_GASTRIC_CANCER_EARLY_ UP | Gastric Cancer | 0.508 | 0.033 | 0.208 | *ACAA2, LAPTM4B, RAD18, CDH3, EPPK1, CASC5, MACC1, OVOL1, TRIM31, CLDN3, CAMK2N1, CDH17* | 4205 |
| VECCHI_GASTRIC_CANCER_ ADVANCED_VS EARLY_DN | Gastric Cancer | 0.614 | 0.004 | 0.171 | *ATP10B, FAM3D, CLDN15, BTNL8, CFTR, REEP6, CLRN3, DHRS11, TM4SF20, GPA33, REG3A* | 2735 |
| KEGG_DRUG_METABOLISM_OTHER_ ENZYMES | Xenobiotic metabolism | 0.673 | 0.007 | 0.162 | *UGT2B7, NAT2, TPMT, UGT2A3, UGT1A6, UGT1A7, CDA, CES2, CYP3A4* | 1348 |
| OHGUCHI_LIVER_HNF4A_TARGETS_ DN | Intestinal differentiation | 0.605 | 0.000 | 0.129 | *CFI, NLRP6, SULT1B1, SLC30A10, SLC3A1, S100A10, ETHE1, HSD17B2, MTTP* | 1356 |
| BAUS_TFF2_TARGETS_UP | Presentation and antigenic processing | 0.789 | 0.000 | 0.167 | *IGHM, RBP2, DGKA, TFF3, ITLN1, ABGC2, ZG16, APOA4* | 952 |
| BROWN_MYELOID_CELL_ DEVELOPMENT_UP | Inflammation | 0.422 | 0.014 | 0.240 | *UGT2B17, HEBP1, CLDN1, CCND2, CEACAM1, XDH, GLRX, ANPEP* | 4270 |
| LABBE_TARGETS_OF_TGFB1_AND_ WNT3A_DN | Cell Proliferation | 0.450 | 0.006 | 0.191 | *TPK1, TFRC, LGALS4, SLC30A4, HNF4A, VIL1, VNN1, SERPINA1* | 2919 |
| ALCALA_APOPTOSIS | Apoptosis | 0.524 | 0.002 | 0.138 | *GPI, MTCH2,CASP1, HADHA, QPRT, ATP1A1* | 3103 |
| KEGG_ARGININE_AND_PROLINE_ METABOLISM | Non tumoral Warburg effect | 0.605 | 0.004 | 0.153 | *NAGS, MAOB, PRODH, AGMAT, OAT, ABP1* | 1415 |
| SERVITJA_ISLET_HNF1A_TARGETS_ DN | Intestinal differentiation | 0.515 | 0.002 | 0.153 | *NR1H4, CDHR2, RNF186, MTMR11, TM4SF4, ACE2* | 1707 |
| REACTOME_AMINO_ACID_AND_ OLIGOPEPTIDE_SLC_TRANSPORTERS | Non tumoral Warburg effect | 0.618 | 0.000 | 0.142 | *SLC15A2, SLC6A20, SLC1A1, SLC15A1, SLC7A9, SLC6A19* | 1480 |
| KEGG_ALANINE_ASPARTATE_AND_ GLUTAMATE_METABOLISM | Non tumoral Warburg effect | 0.563 | 0.018 | 0.242 | *GPT, ASS1, GLS, ACY3, CPS1* | 2144 |
| **Gene sets ^a^** | **Molecular processes ^b^** | **ES ^c^** | **Nominal p-value ^d^** | **q-value FDR ^e^** | **Over-expressed leading edge genes in IIM-GC ^f^** | **Rank at max ^f^** |
| REACTOME_METABOLISM_OF_ CARBOHYDRATES | Non tumoral Warburg effect | 0.379 | 0.002 | 0.234 | *CHST5, B3GNT7, CHST6, SLC2A5, SLC5A1* | 3526 |
| REACTOME_O_LINKED_ GLYCOSYLATION_OF_MUCINS | Aberrant protein glycosylation | 0.565 | 0.002 | 0.168 | *GCNT3, MUC4, MUC13, MUC12, MUC17* | 2594 |
| ACOSTA_PROLIFERATION_ INDEPENDENT MYC_TARGETS_DN | Oncogenes | 0.395 | 0.020 | 0.199 | *SLCO2B1, ARL4A, IRF4, MPP1* | 5253 |
| AUNG_GASTRIC_CANCER | Gastric Cancer | 0.566 | 0.000 | 0.147 | *DEFA6, DEFA5, REG4, OLFM4* | 2619 |
| KEGG_CHEMOKINE_SIGNALING_ PATHWAY | Inflammation | 0.389 | 0.027 | 0.241 | *PLCB3, CCL24, CCL15, CCL25* | 3524 |
| KEGG_CITRATE_CYCLE_TCA_CYCLE | Non tumoral Warburg effect | 0.643 | 0.045 | 0.239 | *SUCLG1, IDH3A, PCK1, PCK2* | 2846 |
| KEGG_GLYCOLYSIS_ GLUCONEOGENESIS | Non tumoral Warburg effect | 0.511 | 0.016 | 0.201 | *GALM, ADH6, LDHA, ALDOB* | 1249 |
| LIU_CDX2_TARGETS_UP | Intestinal differentiation | 0.738 | 0.000 | 0.193 | *KRT20, CDX1, HEPH, MUC2* | 385 |
| REACTOME_PHASE_II_CONJUGATION | Xenobiotic metabolism | 0.647 | 0.015 | 0.202 | *SULT1A1, NAT1, GSTA1, SULT1E1* | 2050 |
| TRANSPORT_OF_GLUCOSE_AND_OTHER_SUGARS_BILE_SALTS_AND_ORGANIC_ACIDS_METAL_IONS_AND_AMINE_COMPOUNDS | Non tumoral Warburg effect | 0.492 | 0.002 | 0.158 | *SLC22A18, SLC39A4, SLC39A5, SLC13A2* | 1446 |
| KEGG_ETHER_LIPID_METABOLISM | Lipid metabolism | 0.532 | 0.029 | 0.246 | *PLA2G12B, PLD1, PLA2G2A* | 1240 |
| KEGG_GLYCEROLIPID_METABOLIS | Lipid metabolism | 0.543 | 0.008 | 0.171 | *DGAT1, DGKQ, DAK* | 1926 |
| LUCAS_HNF4A_TARGETS_UP | Intestinal differentiation | 0.601 | 0.031 | 0.241 | *ATP7B, CALML4, CIDEB* | 1688 |

a Over-expressed gene sets in the IIM-GC. b Functional processes represented by gene sets. c Enrichment score. d p-value of gene sets, unadjusted for multiple corrections. e q-value of gene sets, adjusted by FDR multiple corrections test. f Leading edge genes over-expressed in the IIM-GC. g Position in the ranking list at which the highest value of ES is obtained
